# Feasibility, safety, and impact of the RTS,S/AS01_E_ malaria vaccine when implemented through national immunisation programmes: evaluation of cluster-randomised introduction of the vaccine in Ghana, Kenya, and Malawi

**DOI:** 10.1016/S0140-6736(24)00004-7

**Published:** 2024-04-27

**Authors:** Kwaku Poku Asante, Don P Mathanga, Paul Milligan, Samuel Akech, Abraham Oduro, Victor Mwapasa, Kerryn A Moore, Titus K Kwambai, Mary J Hamel, Thomas Gyan, Nelli Westercamp, Atupele Kapito-Tembo, Patricia Njuguna, Daniel Ansong, Simon Kariuki, Tisungane Mvalo, Paul Snell, David Schellenberg, Paul Welega, Lucas Otieno, Alfred Chimala, Edwin A Afari, Philip Bejon, Kenneth Maleta, Tsiri Agbenyega, Robert W Snow, Madaliso Zulu, Jobiba Chinkhumba, Aaron M Samuels, Malaria Vaccine Programme Evaluation Partners, Sulemana Watara Abubakari, Albert Akumani, Dennis Adu-Gyasi, Augustine Sarfo, Elezier Odei-Lartey, Francis Agbokey, Seeba Amenga-Etego, Stephany Gyaase, Patrick Buabeng, Elizabeth Awini, Justice Sylverken, Aaron Kampim, Kwadwo A Koram, Abraham Hodgson, Fred Newton Binka, Rafiq Nii Attoh Okine, Peter Ofori Tweneboah, Bella Ondiegi, Brian Seda, Dorcas Akach, Gordon Orwa, Isabella Nyang’au, Oscar Odunga, Francis Gumba, Nathanial Copeland, Cynthia Khazenzi, Eda Mumo, Monica Musa, Morris Ogero, Mike English, Adam Haji, Josephine Njoroge, Harrison Msuku, Vincent Samuel, Hillary Topazian Mariko, Jon Juliano, Lusungu Msumba, Randy George Mungwira, Boston Edward Zimba, Meghna Desai, Eliane Furrer, John Aponte, Pedro Alonso, Akpaka A Kalu, Jackson Sophianu Sillah

**Affiliations:** a Kintampo Health Research Centre, Research and Development Division, Ghana Health Service, Kintampo North Municipality, Ghana; b London School of Hygiene & Tropical Medicine, London, UK; c School of Public Health, Kamuzu University of Health Sciences, Blantyre, Malawi; d Malaria Alert Centre, Kamuzu University of Health Sciences, Blantyre, Malawi; e Kenya Medical Research Institute (KEMRI)–Wellcome Trust Research Programme, Nairobi, Kenya; f Navrongo Health Research Centre, Research and Development Division, Ghana Health Service, Accra, Ghana; g Malaria Branch, Division of Parasitic Diseases and Malaria, Center for Global Health, Centers for Disease Control and Prevention, Kisumu, Kenya; h Department of Immunizations, Vaccines, and Biologicals, WHO, Geneva, Switzerland; i Global Malaria Programme, WHO, Geneva, Switzerland; j Centre for Global Health Research, KEMRI, Kisumu, Kenya; k Murdoch Children's Research Institute, Infection and Immunity, New Vaccines, Parkville, VIC, Australia; l Agogo Malaria Research Centre, Agogo, Ghana; m Kwame Nkrumah University of Science and Technology, Kumasi, Ghana; n Malaria Branch, Division of Parasitic Diseases and Malaria, Center for Global Health, Centers for Disease Control and Prevention, Atlanta, GA, USA; o University of North Carolina Project-Malawi, Lilongwe, Malawi; p KEMRI–US Army Medical Research Directorate-Africa, Kisumu, Kenya; q School of Public Health, University of Ghana, Accra, Ghana; r Centre for Tropical Medicine and Global Health, Nuffield Department of Clinical Medicine, University of Oxford, Oxford, UK

## Abstract

**Background:**

The RTS,S/AS01_E_ malaria vaccine (RTS,S) was introduced by national immunisation programmes in Ghana, Kenya, and Malawi in 2019 in large-scale pilot schemes. We aimed to address questions about feasibility and impact, and to assess safety signals that had been observed in the phase 3 trial that included an excess of meningitis and cerebral malaria cases in RTS,S recipients, and the possibility of an excess of deaths among girls who received RTS,S than in controls, to inform decisions about wider use.

**Methods:**

In this prospective evaluation, 158 geographical clusters (66 districts in Ghana; 46 sub-counties in Kenya; and 46 groups of immunisation clinic catchment areas in Malawi) were randomly assigned to early or delayed introduction of RTS,S, with three doses to be administered between the ages of 5 months and 9 months and a fourth dose at the age of approximately 2 years. Primary outcomes of the evaluation, planned over 4 years, were mortality from all causes except injury (impact), hospital admission with severe malaria (impact), hospital admission with meningitis or cerebral malaria (safety), deaths in girls compared with boys (safety), and vaccination coverage (feasibility). Mortality was monitored in children aged 1–59 months throughout the pilot areas. Surveillance for meningitis and severe malaria was established in eight sentinel hospitals in Ghana, six in Kenya, and four in Malawi. Vaccine uptake was measured in surveys of children aged 12–23 months about 18 months after vaccine introduction. We estimated that sufficient data would have accrued after 24 months to evaluate each of the safety signals and the impact on severe malaria in a pooled analysis of the data from the three countries. We estimated incidence rate ratios (IRRs) by comparing the ratio of the number of events in children age-eligible to have received at least one dose of the vaccine (for safety outcomes), or age-eligible to have received three doses (for impact outcomes), to that in non-eligible age groups in implementation areas with the equivalent ratio in comparison areas. To establish whether there was evidence of a difference between girls and boys in the vaccine's impact on mortality, the female-to-male mortality ratio in age groups eligible to receive the vaccine (relative to the ratio in non-eligible children) was compared between implementation and comparison areas. Preliminary findings contributed to WHO's recommendation in 2021 for widespread use of RTS,S in areas of moderate-to-high malaria transmission.

**Findings:**

By April 30, 2021, 652 673 children had received at least one dose of RTS,S and 494 745 children had received three doses. Coverage of the first dose was 76% in Ghana, 79% in Kenya, and 73% in Malawi, and coverage of the third dose was 66% in Ghana, 62% in Kenya, and 62% in Malawi. 26 285 children aged 1–59 months were admitted to sentinel hospitals and 13 198 deaths were reported through mortality surveillance. Among children eligible to have received at least one dose of RTS,S, there was no evidence of an excess of meningitis or cerebral malaria cases in implementation areas compared with comparison areas (hospital admission with meningitis: IRR 0·63 [95% CI 0·22–1·79]; hospital admission with cerebral malaria: IRR 1·03 [95% CI 0·61–1·74]). The impact of RTS,S introduction on mortality was similar for girls and boys (relative mortality ratio 1·03 [95% CI 0·88–1·21]). Among children eligible for three vaccine doses, RTS,S introduction was associated with a 32% reduction (95% CI 5–51%) in hospital admission with severe malaria, and a 9% reduction (95% CI 0–18%) in all-cause mortality (excluding injury).

**Interpretation:**

In the first 2 years of implementation of RTS,S, the three primary doses were effectively deployed through national immunisation programmes. There was no evidence of the safety signals that had been observed in the phase 3 trial, and introduction of the vaccine was associated with substantial reductions in hospital admission with severe malaria. Evaluation continues to assess the impact of four doses of RTS,S.

**Funding:**

Gavi, the Vaccine Alliance; the Global Fund to Fight AIDS, Tuberculosis and Malaria; and Unitaid.

## Introduction

There were an estimated 247 million malaria cases and 619 000 malaria deaths worldwide in 2021; the vast majority occurred in young children in sub-Saharan Africa.^1^ WHO estimates of key indicators of global malaria burden have not improved since 2015, attributed in part to rising insecticide resistance, humanitarian crises, and other health system challenges.^1^ In areas with high malaria burden, transmission is so intense that children are still at appreciable risk even when using all recommended interventions.^2^ Therefore, additional malaria control tools are urgently needed.

In 2015, the European Medicines Agency reviewed data from a multicentre phase 3 trial of the RTS,S/AS01_E_ malaria vaccine (henceforth RTS,S)^3^ and earlier studies, and gave a positive scientific opinion under Article 58, for both age groups studied in the phase 3 trial: infants aged 6–14 weeks and children aged 5–17 months.^4^ WHO then considered the potential use of RTS,S and recommended large-scale pilot implementation of the four-dose regimen in children aged at least 5 months at the first dose, in settings with moderate-to-high malaria transmission. The pilot was designed to assess: the feasibility of implementing four doses of the vaccine in a schedule beyond regular contacts of the Expanded Program on Immunization (EPI); the vaccine's safety; and the impact of vaccine introduction on mortality and on the incidence of hospital admission with severe malaria.^5^ The vaccine was not recommended in the 6–14-week age group due to lower efficacy in this group than in the 5–17-month age group in the phase 3 trial.^6^ With respect to safety, the focus was on three signals seen in the phase 3 trial, none of which were temporally associated with vaccination: an excess of meningitis and cerebral malaria cases in recipients of RTS,S, and an interaction between sex and vaccine group with respect to effects on mortality (there appeared to be a deficit of deaths among girls in the control group, but the possibility of an excess of deaths among girls in the vaccine group could not be excluded).^7^

**Research in context**

**Evidence before this study**

We searched the PubMed database for phase 2 or 3 clinical trials and systematic reviews of the RTS,S/AS01_E_ vaccine published between Jan 1, 2009 and Oct 7, 2022, with no language restrictions and using the search terms ((“RTS,S/AS01”[all fields] AND (“safety”[all fields] OR “meningitis”[all fields] OR “cerebral malaria”[all fields] OR “feasibility”[all fields] OR “coverage”[all fields] OR “mortality”[all fields] OR “impact”[all fields] OR “cases-averted”[all fields] OR “deaths-averted”[all fields]))). We identified 25 publications describing data from 14 trials, including one large, multicentre, phase 3 trial. All the trials found that RTS,S/AS01_E_ had a satisfactory safety profile but, in the multicentre phase 3 trial, there were three safety signals—an excess of meningitis and cerebral malaria cases in RTS,S/AS01_E_ recipients, and, among girls, more deaths in those who received RTS,S/AS01_E_ than in controls. These safety signals were unexplained, had no temporal association with vaccination, and were not detected in a subsequent analysis of pooled phase 2 data. In the multicentre phase 3 trial, among children aged 5–17 months at the first dose who received three vaccine doses, the incidence of clinical malaria was reduced by 56% and the incidence of severe malaria was reduced by 47% over 1 year; among children who received a fourth dose (18 months after the third dose), the incidence of clinical malaria was reduced by 39% and the incidence of severe malaria was reduced by 32% over 4 years. It appeared that the fourth vaccine dose was necessary to reduce a child's overall risk of severe malaria over the 4 years of the trial, although there was uncertainty regarding this conclusion. For every 1000 children vaccinated with four doses, 1774 cases of malaria and 40 hospital admissions due to malaria were averted over about 4 years and models predicted the vaccine would be cost-effective, especially in high-transmission settings. Clinical trials of seasonal vaccination with RTS,S/AS01_E_ (three primary doses followed by two annual booster doses) showed, over 3 years, a vaccine efficacy of 63% against clinical malaria, 70% against hospital admission with severe malaria, and 73% against deaths from malaria, in children receiving seasonal malaria chemoprevention.

**Added value of this study**

RTS,S/AS01_E_ is the world's first licensed malaria vaccine. This is the first study to evaluate a malaria vaccine when introduced as part of national immunisation programmes. A robust evaluation of feasibility, safety, and impact was done using standardised methods of surveillance in implementation and comparison areas chosen by randomisation. Results after 2 years show that more than 70% of children aged 1 year had received a first dose of the vaccine, with no evidence of the safety signals that had been observed in the earlier phase 3 clinical trial. In the age groups of children who would have been eligible to have received three doses of the malaria vaccine, introduction of the vaccine was associated with a reduction in hospital admissions with severe malaria by 32%, and a reduction in child deaths of any cause (excluding injury) of 9%. The impact was realised in the context of moderate coverage and during the COVID-19 pandemic.

**Implications of all the available evidence**

Implementation of the RTS,S/AS01_E_ malaria vaccine into routine immunisation programmes can have a significant impact in reducing severe illness and deaths in young children caused by *Plasmodium falciparum* malaria. WHO recommended in 2021 that the vaccine should be provided for children in all regions with moderate-to-high malaria transmission. GAVI, The Vaccine Alliance, has added malaria vaccination to its portfolio, but supplies of the RTS,S/AS01_E_ vaccine are projected to fall far short of the number of doses needed. The evidence from our study and those of others show that efforts to increase malaria vaccine supply and widen its introduction should be accelerated as a matter of urgency.

Health authorities in Ghana, Kenya, and Malawi authorised the use of the vaccine in pilot areas in 2018, and the national EPI in each country did a phased introduction of the vaccine in pilot areas in April (Ghana and Malawi) and September (Kenya), 2019. The evaluation was planned over 4 years, with primary analysis of the safety signals and the initial impact on the incidence of hospital admission with severe malaria planned at 24 months, because it was anticipated that there would be sufficient data by that time to inform a recommendation for wider use of the vaccine.^6^

In October, 2021, WHO recommended that RTS,S should be used in all areas with moderate-to-high transmission to prevent *Plasmodium falciparum* malaria in young children.^8^ Here, we present the evaluation of feasibility, safety, and impact of RTS,S in Ghana, Kenya, and Malawi (with a focus on the primary three doses), which contributed to this recommendation, based on data accrued over 24 months up to April 30, 2021. Evaluation will continue for 46 months of follow-up in each country, providing information on uptake and impact of four doses and the overall impact on mortality.

## Methods

### Selection of evaluation areas

Participating countries were selected among applicant countries on the basis of having regions with moderate-to-high, year-round malaria transmission and good coverage of childhood vaccinations and core malaria interventions (appendix pp 7, 53–56).^9^ Surveillance for child mortality was strengthened throughout these pilot regions; hospital-based surveillance for severe malaria and other conditions was limited to part of each region served by selected sentinel hospitals, in which clinical and laboratory investigations were strengthened.

### Randomisation

Within the pilot region in each country, the EPI defined clusters, each with a population of about 100 000 and about 4000 births per year. 158 clusters were randomised (66 districts in Ghana, 46 sub-counties in western Kenya, and 46 groups of immunisation clinics and their associated catchment areas in Malawi). An independent statistician assisted the EPI to randomly assign clusters to introduce RTS,S in 2019 (implementing areas) or to delay introduction (comparator areas; appendix pp 7–8). Constrained randomisation in each country was used to balance implementing and comparator areas with respect to the number of infants surviving to 12 months, malaria parasite prevalence, EPI coverage (pentavalent vaccine dose 3 and measles vaccine dose 1), and number of hospitals, health centres, and dispensaries. Additional balance criteria within areas defined for hospital surveillance were the number of sentinel hospitals and access to hospital as measured by estimates of the number of patients admitted to hospitals from each cluster before vaccine introduction; however, in Kenya, these balance criteria resulted in overly restricted allocation options and were relaxed. A community representative in each country selected the final allocation at random from a list of approximately balanced randomisation options.

### Surveys to monitor vaccine uptake

Large representative household surveys were done in implementing areas of each country at baseline and after about 18 months to measure coverage of EPI vaccines, including RTS,S, and use of long-lasting insecticide-treated bednets (LLINs), ask about care-seeking for fever, and establish the prevalence of *P falciparum* malaria using a malaria rapid diagnostic test. Caregivers were asked about the child's vaccination history, and vaccination dates were recorded from the home-based vaccination record if available (appendix pp 8–16).

### Sentinel hospital surveillance

Sentinel referral hospitals were selected (eight in Ghana, six in Kenya, four in Malawi) that had a high volume of paediatric admissions and were able to collect and analyse cerebrospinal fluid (CSF) for diagnosis of meningitis and cerebral malaria. Laboratory capacity was strengthened and hospital staff supported to do clinical investigation using standardised case definitions for malaria, severe malaria, cerebral malaria, and meningitis (appendix pp 38–39) through training and supervision, quality assurance, and provision of lumbar puncture kits and laboratory reagents (appendix pp 17–22). Staff were trained to extract data on patient age, usual residence, vaccination status, clinical history and examination, laboratory results, and discharge diagnosis from patient case-notes for all admitted children aged 1–59 months. For children with suspected meningitis or cerebral malaria, lumbar puncture was performed and collected CSF was analysed locally to determine clinical care; an aliquot was sent for pathogen genotyping by PCR at reference laboratories.

### Mortality surveillance

Surveillance for mortality was established in the community throughout the implementation and comparison areas. When a child death was reported, an interviewer visited to confirm place of usual residence, sex, and age at death, and complete a verbal autopsy using the WHO verbal autopsy instrument to evaluate cause of death (appendix pp 23–26). RTS,S vaccine status was recorded from the child's vaccination book (home-based record) or by maternal recall, or both. Cause of death was ascertained using the InterVA software or, for deaths in a health facility in Malawi, from the death notification form.

### Vaccine introduction

Doses were scheduled at age 6, 7, 9, and 24 months in Ghana and Kenya, and age 5, 6, 7, and 22 months in Malawi (appendix p 57). At the time of RTS,S introduction, the first dose could be given up to and including the age of 7 months in Ghana, 11 months in Kenya, and 5 months in Malawi. The upper age limits for the first dose in Ghana and Malawi were later modified to 11 months. The vaccine was introduced on April 23, 2019 in Malawi, April 30, 2019 in Ghana, and Sept 13, 2019 in Kenya (appendix p 27).

### Statistical methods

#### Primary outcomes

Vaccination coverage was the primary outcome for feasibility. The primary outcomes for safety were hospital admission with meningitis or cerebral malaria and death due to any cause (excluding injury) compared between girls and boys. Mortality from all causes (except injury) and hospital admission with severe malaria were the primary outcomes for impact.

In patients with suspected meningitis, meningitis was classified as probable meningitis on the basis of macroscopic and microscopic examination of CSF or as confirmed meningitis on the basis of a positive CSF PCR result for bacterial or viral causes. For cases to be classified as cerebral malaria, they had to be *P falciparum*-positive (antigenaemia detected by rapid diagnostic test, or parasitaemia detected by microscopy if a rapid diagnostic test was not done) with impaired consciousness (Glasgow Coma Scale score <11, Blantyre coma score <3, or assessed as pain or unresponsive on Alert, Voice, Pain, Unresponsive score) in a patient that did not meet any meningitis definition (appendix pp 38–39). Severe malaria was defined as admission to hospital with a positive malaria test, with severe anaemia, respiratory distress, impaired consciousness (and not positive for meningitis), or convulsions (and not positive for meningitis; appendix pp 38–39). Although all cerebral malaria cases—and severe malaria cases with impaired consciousness or convulsions—also met the criteria for suspected meningitis, not all had lumbar puncture done; however, if lumbar puncture was done and CSF findings were consistent with probable or confirmed meningitis, those cases were excluded. Additional analyses were done for severe malaria and cerebral malaria, excluding cases that met the definition of suspected meningitis but for which meningitis status could not be established (eg, due to lumbar puncture not being done; appendix pp 38–39, 64–65). All events occurring from the first date of vaccine implementation in each country to April 30, 2021 in children living in the pilot areas (for mortality) or living in defined hospital surveillance areas (for hospital outcomes) were included.

#### Analysis methods

Coverage estimates were obtained using standard methods for surveys (appendix pp 30–31). Incidence rate ratios (IRRs) were estimated comparing the incidence between RTS,S implementation areas and comparison areas among age groups eligible to receive the first dose of the RTS,S vaccine for safety analyses, and among age groups eligible to have received a third dose for impact analyses (appendix pp 28–32). Stata, version 15, was used for the analyses. IRRs were estimated as a double ratio of event counts,^10^ comparing the ratio of events between eligible and non-eligible age groups in RTS,S implementation areas with that in comparison areas (appendix pp 28–34, 58). Eligibility was defined according to the child's age at admission or death (determined by date of birth or recalled age), the date of vaccine introduction, and the country-specific schedule (appendix p 38). Children just above the eligible age limit (by up to 2 months) were excluded. Pooled estimates of IRRs were obtained by weighting the logarithm of country estimates by the inverse of their variance (appendix pp 28–34). The female-to-male mortality ratio in vaccine-eligible age groups was compared between implementation and comparison areas similarly, using data in non-eligible boys and girls as auxiliary variables (appendix pp 28–32).

We estimated the number of events required for 90% power to detect a difference between implementation and comparison areas, for each safety outcome, if the safety signal occurred at the level observed in the phase 3 trial, after allowing for dilution of effects due to incomplete RTS,S coverage and contamination (appendix p 31). Subsequently, data from coverage surveys were used to calculate revised dilution factors (appendix p 32).^11^ With respect to mortality, the evaluation was similarly powered to detect a difference in impact of RTS,S between girls and boys, allowing for dilution. We also estimated the number of severe malaria admissions required for 90% power to detect a reduction if RTS,S effectiveness was similar to the efficacy in the phase 3 trial, allowing for dilution. We estimated that sufficient events of each safety outcome, and of severe malaria, would have accrued 24 months after the start of the evaluation and we thus planned primary analyses of these outcomes at this time. Analyses followed a predefined analysis plan^12^ and framework for policy making,^6^ which specified that a broader recommendation on RTS,S could be made if the safety signals were not replicated and the data on severe malaria and mortality were consistent with a benefit, recognising that primary analysis of the impact on mortality and assessment of the value of the fourth dose would be done at the end of the 4-year evaluation.

Consent of a parent or guardian was sought for data collection following approval by the institutional review boards of the evaluation partners’ institutions and WHO (appendix pp 35–36). Sentinel surveillance in Kenya used an established routine system and parental consent was only obtained for CSF storage.^13^ The evaluation is registered on ClinicalTrials.gov (NCT03806465) as an observational study. An independent data safety monitoring board and the Malaria Vaccine Implementation Programme Advisory Group provided oversight.

### Role of the funding source

The funders of the study had no role in study design, data collection, data analysis, data interpretation, or writing of the report.

## Results

In implementation areas surveyed before RTS,S was introduced, coverage of the third dose of the pentavalent vaccine (diphtheria–pertussis–tetanus + *Haemophilus influenzae* type B + hepatitis B) was 93% in Ghana, 92% in Kenya, and 88% in Malawi among children aged 12–23 months (table). Coverage of the first dose of measles-containing vaccine was 90% in Ghana, 85% in Kenya, and 85% in Malawi among children aged 12–23 months; the coverage of the second dose of measles-containing vaccine was 83% in Ghana, 48% in Kenya, and 63% in Malawi among children aged 24–35 months. Coverage of these vaccines in comparison areas was similar to that in implementation areas. Use of LLINs in implementation areas among children aged 5–48 months was 63% in Ghana, 86% in Kenya, and 90% in Malawi, whereas use of LLINs in comparison areas was 60% in Ghana, 89% in Kenya, and 92% in Malawi. The prevalence of *P falciparum* according to rapid diagnostic tests in children aged 5–48 months was 21% (Ghana), 26% (Kenya), and 28% (Malawi) in implementation areas and 20% (Ghana), 19% (Kenya), and 17% (Malawi) in comparison areas (table).

**Table:** Characteristics of the evaluation areas before RTS,S introduction

	**Ghana**		**Kenya**		**Malawi**	
	Implementation	Comparison	Implementation	Comparison	Implementation	Comparison
**All pilot evaluation areas**						
Number of clusters	33	33	23	23	23	23
Population of surviving infants*	128 624	133 702	126 698	125 747	107 728	113 997
Surviving infants per cluster, range*	912–7026	1202–8954	3736–8805	2702–10 739	2816–6931	3026–8112
Malaria prevalence (age 5–48 months)	21%	20%	26%	19%	28%	17%
LLIN use (age 5–48 months)	63%	60%	86%	89%	90%	92%
DTP dose 3 coverage (age 12–23 months)	93%	96%	92%	91%	88%	89%
MR dose 1 coverage (age 12–23 months)	90%	91%	85%	91%	85%	82%
MR dose 2 coverage (age 24–35 months)	83%	80%	48%	51%	63%	69%
**Hospital surveillance areas**						
Number of clusters	15	17	16	12	8	9
Population of surviving infants*	71 992	76 097	87 824	67 836	37 908	49 039
Surviving infants per cluster, range*	1379–7026	1202–8954	3736–8805	3487–10 739	2816–6931	3670–8112
Malaria prevalence (age 5–48 months)	15%	18%	27%	14%	16%	12%
LLIN use (age 5–48 months)	57%	56%	86%	89%	91%	92%
DTP dose 3 (age 12–23 months)	90%	95%	91%	93%	88%	88%
MR dose 1 (age 12–23 months)	88%	90%	84%	93%	85%	81%
MR dose 2 (age 24–35 months)	80%	78%	47%	55%	65%	72%

LLIN=long-lasting insecticide-treated nets. DTP=diphtheria, tetanus, and pertussis vaccine. MR=measles and rubella vaccine.

* Estimated number born per year who survive to age 12 months (2018 data supplied by the Expanded Programme on Immunization in each country; all other data presented were obtained from baseline surveys in each country). RTS,S=RTS,S/AS01_E_ malaria vaccine.

**Figure 1:**
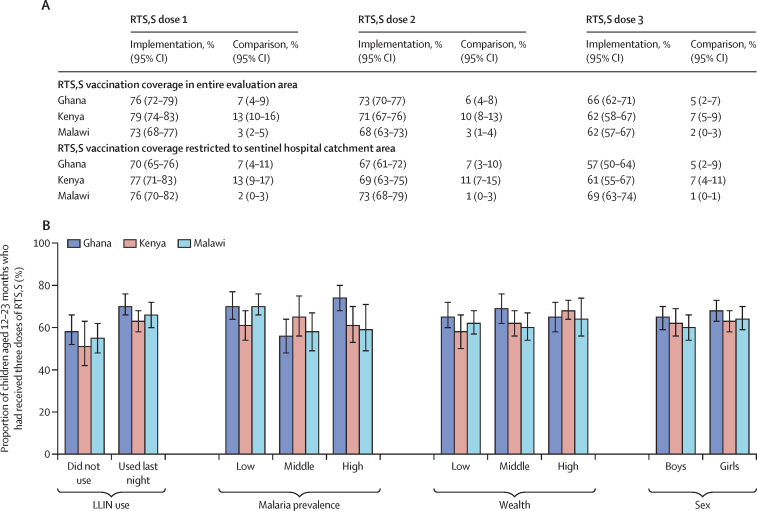
Uptake of the RTS,S vaccine (A) Coverage of the first, second, and third doses of RTS,S among children aged 12–23 months in surveys done about 18 months after introduction of RTS,S. (B) Uptake of the primary three doses of RTS,S, in relation to use of LLINs, malaria transmission intensity, socioeconomic status, and sex. Vertical bars indicate 95% CIs. The percentage that slept under an LLIN the night before the survey was 67% in Ghana, 93% in Kenya, and 68% in Malawi. RTS,S uptake was documented from a home-based record (in 92% of children in Ghana, 88% in Kenya, and 91% in Malawi) or from caregiver recall for those without a record. Malaria prevalence was categorised according to tertiles of malaria prevalence among children aged 5–48 months. Wealth was categorised according to tertiles of principal component scores based on household assets. The prevalence ratios (and 95% CIs) in the proportion of children who had received the third dose of RTS,S between categories were 1·20 (1·05–1·38) in Ghana, 1·23 (1·01–1·50) in Kenya, and 1·20 (1·04–1·37) in Malawi for LLIN users compared with non-users; 1·04 (0·92–1·18), 1·00 (0·84–1·20), and 0·84 (0·69–1·02) for higher malaria prevalence compared with lower; 1·00 (0·88–1·13), 1·18 (1·04–1·35), and 1·04 (0·90–1·19) for upper wealth ranking compared with lower wealth ranking; and 1·06 (0·97–1·15), 1·01 (0·90–1·13), and 1·07 (0·99–1·16) for girls compared with boys. LLIN=long-lasting insecticide-treated net. RTS,S=RTS,S/AS01_E_ malaria vaccine.

**Figure 2:**
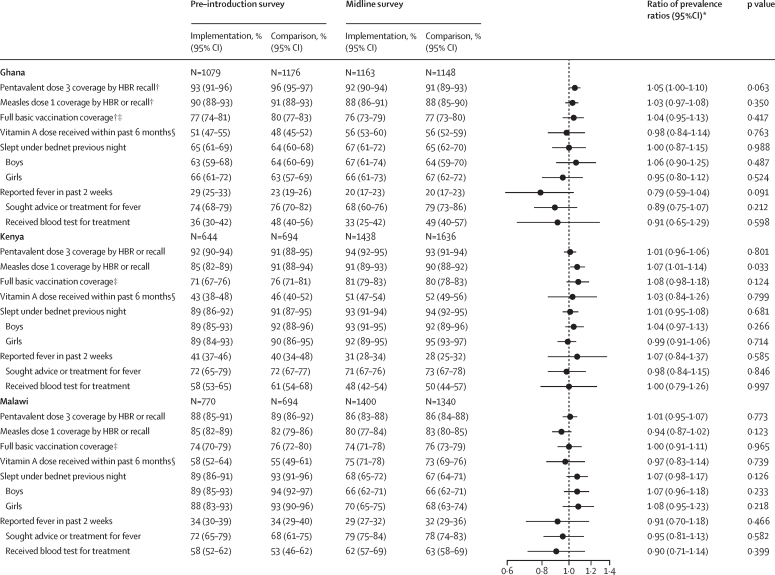
Vaccination and vitamin A coverage, LLIN use, and health-care-seeking behaviour by country, area, and survey Indicators are within children aged 12–23 months at the time of the survey, except for LLIN use, which is within children aged 5–48 months. To assess whether vaccine introduction was associated with any change in coverage of other vaccines, coverage of LLIN use, care-seeking for fever, or use of vitamin A, the ratio of the prevalence in the midline survey to that at baseline was compared between implementing and comparison areas. The relative ratio (ratio of the prevalence ratio in implementing areas to that in comparison areas) is shown in the forest plots. *Ratio of prevalence ratio of pre-introduction and midline surveys. Equivalent numbers restricted to the sentinel areas are available in the appendix (p 59). †Vaccination coverages in Ghana are from HBR only in the baseline survey because vaccination status by recall was not captured; midline coverages presented are from HBR or recall; however, for comparability, the prevalence ratios for midline versus baseline surveys in Ghana use coverage by HBR only. ‡Full basic vaccination coverage refers to BCG at birth, three doses of OPV (excluding birth dose), three doses of the pentavalent vaccine, and one dose of the measles vaccine; children who received IPV at 14 weeks in place of the third dose of OPV were considered to be fully vaccinated for polio; IPV status was only available from recall in Malawi, only available from HBR in Ghana, and not available in Kenya by either method. §Vitamin A coverage was determined from HBR only in Ghana, recall only in Malawi, and both HBR and recall in Kenya. HBR=home-based record. LLIN=long-lasting insecticide treated-net. RTS,S=RTS,S/AS01_E_ malaria vaccine. OPV=oral poliovirus vaccine. IPV=inactivated poliovirus vaccine.

By April 30, 2021, 652 673 children had received their first dose: 238 318 in Ghana, 187 857 in Kenya, and 226 498 in Malawi. 494 745 children had received their third dose (173 552 in Malawi, 200 398 in Ghana, and 120 795 in Kenya) and 79 523 children had received their fourth dose (35 209 in Ghana, 10 805 in Kenya, 33 509 in Malawi). About 18 months after introduction of the vaccine, among children aged 12–23 months surveyed in implementation areas in Ghana, 76% (95% CI 72–79%) had received their first dose of RTS,S and 66% (62–71%) had received the third dose; in Kenya, 79% (74–83%) had received the first dose and 62% (58–67%) had received the third dose, whereas in Malawi, 73% (68–77%) had received the first dose and 62% (57–67%) had received the third dose (figure 1A). In each country, vaccine uptake was similar in girls and boys and across wealth rankings, except in Kenya where children in the upper third for wealth were more likely to have received three doses of RTS,S than children in the lower third (figure 1B). In each country, children who slept under an LLIN were more likely to have received three doses of RTS,S than children who did not sleep under an LLIN (figure 1B). There was no evidence that introduction of RTS,S affected uptake of other vaccines, vitamin A, use of LLINs, or care-seeking for fever (figure 2). Similar results were observed in sentinel hospital surveillance areas (appendix p 59).

**Figure 3:**
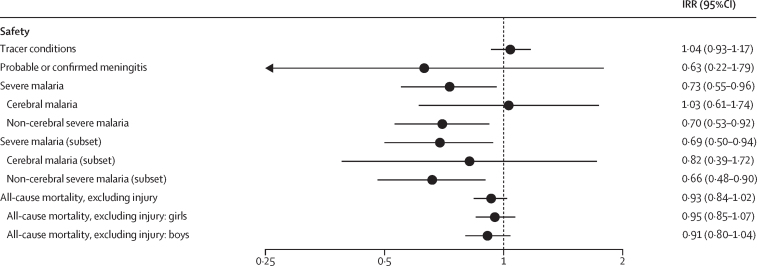
IRRs for safety outcomes in children eligible for at least one dose of RTS,S IRRs are presented for outcomes identified as safety signals in the phase 3 trial. Cerebral and severe malaria is shown for all cases and for the subset excluding cases that met the definition of suspected meningitis but for which meningitis status could not be determined (eg, due to a lumbar puncture not being done). Tracer conditions are those unlikely to be influenced by the RTS,S vaccine (admissions of any cause, excluding those with malaria, anaemia, or meningitis). IRR=incidence rate ratio. RTS,S=RTS,S/AS01_E_ malaria vaccine.

**Figure 4:**
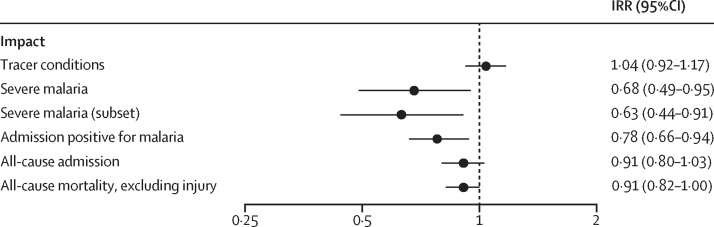
IRRs for impact outcomes in children eligible for three doses of RTS,S IRRs are presented comparing incidence between RTS,S implementation and comparison areas among children eligible to have received three doses of RTS,S. Severe malaria is shown for all cases and for the subset excluding cases that met the definition of suspected meningitis but for which meningitis status could not be determined (eg, due to a lumbar puncture not being done). Tracer conditions are those unlikely to be influenced by the RTS,S vaccine (admissions of any cause, excluding those with malaria, anaemia, or meningitis). IRR=incidence rate ratio. RTS,S=RTS,S/AS01_E_ malaria vaccine

31 072 children aged 1–59 months were admitted to sentinel hospitals from the date of RTS,S introduction up to April 30, 2021, of whom 29 356 resided in defined hospital surveillance areas, had parental consent, and had a date of birth or age documented to determine vaccine eligibility (appendix pp 60–61). Among patients with fever or history of fever (22 463 [81·4%] of 27 596), 21 036 (93·6%) had a malaria test result. 4964 patients from implementation areas were eligible to receive their first RTS,S dose (including 3570 who were eligible to have received three doses), 8231 were not eligible (because they were too young or too old when RTS,S was introduced), and 687 were excluded from analyses as they were just too old for RTS,S by a margin of up to 2 months. Corresponding numbers from comparison areas were 5239 (eligible for first dose), 3887 (eligible for three doses), 7851 (not eligible), and 624 (excluded; appendix pp 60–61). The IRR for conditions of any cause (excluding patients with malaria infection, anaemia, or meningitis) was 1·04 (95% CI 0·93–1·17) among children eligible for their first dose of RTS,S (figure 3), and 1·04 (0·92–1·17) among those eligible to have received three doses (figure 4), showing that implementation and comparison areas were similar with respect to admission with conditions that were unlikely to be affected by the malaria vaccine (appendix pp 63–65).

14 663 deaths in children aged 1–59 months were reported to April 30, 2021, with 14 660 ascertained as resident in Malaria Vaccine Pilot Evaluation areas at the time of death. Of the 5220 children in eligible age groups, 5035 (96·5%) had a verbal autopsy completed or facility death notification obtained. A cause of death (categorised as due to injury or other causes) was established for 4520 (89·8%) of 5035 (appendix p 62).

There were 4136 admissions (eligible and non-eligible age groups) with suspected meningitis. Lumbar punctures were done in 2570 (62·1%) of 4136, of which 2138 (83·2%) had a white cell count recorded and 2438 (94·9%) had the macroscopic appearance of the CSF recorded; PCR results were obtained for 2123 (82·6%) of 2570. Criteria for probable or confirmed meningitis were met in 132 patients, 72 from implementation areas (28 in age groups eligible to receive RTS,S-1) and 60 from comparison areas (25 in age groups eligible to receive RTS,S-1; appendix p 63). The IRR comparing incidence of probable or confirmed meningitis in eligible age groups between RTS,S and comparison areas was 0·63 (95% CI 0·22–1·79; figure 3). Reasons why lumbar punctures were not done and sensitivity analyses using imputed data for those without lumbar puncture (which showed similar results) are detailed in the appendix (p 42). PCR results identified vaccine-preventable bacteria (*H influenzae* type B or vaccine serotypes of *Streptococcus pneumoniae*) in eight (14·8%) of 54 samples from confirmed meningitis cases (appendix pp 44–45).

There were 1423 cases of severe malaria among children eligible to have received RTS,S-1: 568 from implementation areas, of which 52 had cerebral malaria, and 855 from comparison areas, of which 56 had cerebral malaria (appendix p 64). The IRR comparing incidence of cerebral malaria between implementation and comparison areas was 1·03 (95% CI 0·61–1·74; figure 3). When cases of unknown meningitis status were excluded, there were 59 cases (27 from implementation and 32 from comparison areas) and the IRR was 0·82 (0·39–1·72; figure 3).

There were 4748 deaths (excluding those due to injury) in children eligible for RTS,S-1: 2386 from implementation areas (1220 girls and 1166 boys) and 2362 from comparison areas (1265 girls and 1097 boys; appendix p 62). There were 8450 deaths in non-eligible age groups (2304 girls and 2115 boys from implementation areas and 2149 girls and 1882 boys from comparison areas). The mortality ratio associated with RTS,S introduction (both sexes combined) was 0·93 (95% CI 0·84–1·02), with no evidence of a difference between girls and boys. The mortality ratio was 0·95 (0·85–1·07) in girls and 0·91 (0·80–1·04) in boys (figure 3); the relative mortality ratio (girls:boys) was 1·03 (0·88–1·21, p=0·71; appendix pp 46–49).

Among children eligible for RTS,S-3, there was a 32% reduction in hospital admissions with severe malaria (427 in the intervention areas and 697 from comparison areas; IRR 0·68 [95% CI 0·49–0·95]). There was no evidence that impact on severe malaria differed between cerebral and other types of severe malaria (interaction test p=0·10). Within the severe malaria subset analysis (excluding cases also meeting the criteria for suspected meningitis but with unknown meningitis status), the number of cases was 333 in intervention areas and 557 in comparison areas (IRR 0·63 [44–0·91]), a 37% reduction (with no interaction by type of severe malaria, p=0·88). There were 3436 hospital admissions for any cause from implementation areas and 3767 from comparison areas (IRR 0·91 [0·80–1·03]; appendix p 65). The number of admissions with a positive malaria test was 1163 in the implementation area and 1652 in the comparison area (IRR 0·78 [0·66–0·94]). The number of deaths, excluding those due to injury, was 1589 in implementation areas and 1631 in comparison areas (IRR 0·91 [0·82–1·00]; figure 4). There was no evidence that the impact on mortality differed between girls and boys (interaction test p=0·81; appendix pp 46–51).

## Discussion

The comprehensive evaluation reported in this Article was planned to assess the impact of RTS,S, the world's first malaria vaccine, and resolve specific safety signals to inform decisions about wider scale-up of the vaccine as soon as was possible. By April, 2021, about 1·8 million doses of RTS,S had been administered through routine child immunisation clinics. 18 months after introduction, more than 70% of children aged 1 year had received their first dose and more than 60% had received three doses, with similar coverage across socioeconomic groups and in boys and girls. By 24 months after introduction in Malawi and Ghana, and by 19 months after introduction in Kenya (which started later than the other two countries), no evidence was found of the safety signals that had been observed in the phase 3 trial;^3^, ^7^ among the cohorts of children who could have received three doses of the vaccine, there was a reduction in hospital admissions with severe malaria by 32%. Total hospital admissions of any cause were 9% lower in implementation areas than in comparison areas, and deaths of all causes (excluding those due to injury) were also 9% lower. These values are consistent with the important contribution severe malaria makes to hospital admissions and deaths at home or in hospital in these populations.^1^ Findings from this study were reported to WHO between July and September, 2021,^11^ and constituted crucial evidence that informed WHO's recommendation on Oct 6, 2021, that the RTS,S vaccine should be widely implemented in sub-Saharan Africa and in other regions with moderate-to-high *P falciparum* malaria transmission^14^ to prevent malaria in young children. The evaluation will continue to assess the uptake and impact of the full, four-dose schedule of RTS,S, including impact on mortality over a period of 46 months since first introduction in each country.

For this evaluation, standardised surveillance methods were used, diagnostic procedures in hospital were strengthened, and community mortality surveillance strengthened throughout the pilot study regions. Health ministries chose implementation areas using randomisation to ensure robustness of the evaluation. Vaccine delivery was independently assessed through large household surveys.

The high coverage (>60%) of the primary three doses of the vaccine within 18 months of vaccine introduction is notable given the need for additional visits outside the usual EPI schedule and the challenges of maintaining vaccine delivery during the COVID-19 pandemic. This success reflects the commitment of health staff and positive caregiver perceptions of the vaccine as was noted in qualitative observations of caregiver cohorts reported elsewhere.^15^ Caregivers increasingly expressed positive attitudes and trust in RTS,S during the study period, having seen the health benefits of the vaccine in their children and their community. However, coverage of the first dose of RTS,S was lower than coverage of the first dose of measles vaccine, indicating missed opportunities to receive RTS,S. Our findings reflect the impact of the primary three doses, because few children had received the fourth dose by the time of our analysis.^16^, ^17^, ^18^, ^19^

We found no evidence that introduction of RTS,S was associated with an increase in cases of meningitis, cerebral malaria, or deaths in girls—safety signals that had been observed in the phase 3 trial. Our evaluation was powered to detect these signals if they had occurred during the pilot implementation, taking into account the fact that the effects would have been diluted by incomplete coverage and contamination.

For meningitis and the interaction of vaccine impact on mortality by sex, our estimates were not consistent with signals in the phase 3 trial, on the basis of a comparison of our estimates with those predicted if the signals observed in the phase 3 trial had occurred, using data on observed uptake of RTS,S to derive revised dilution factors (appendix pp 32–34).^11^ For cerebral malaria, there was more uncertainty and our estimates did not exclude the diluted effect.

The observed impact of RTS,S on hospital admission with severe malaria is consistent with the impact that would be expected on the basis of the efficacy observed in the phase 3 trial, given the level of uptake of the vaccine in implementation areas. In the phase 3 trial, efficacy against clinical malaria waned over time: 68% in the first 6 months following dose 3 and 55% during the first 12 months. Many of the children who would have received three doses and contributed person-time in our evaluation would have benefited during that time from the relatively high efficacy in the months following the third dose.

There was no evidence that RTS,S introduction influenced the use of LLINs by children, health-seeking behaviour for fever, or the uptake of other vaccines. Although the absence of a negative impact is reassuring, a positive impact of the specific health worker training (to look for missed vaccinations at every visit, to tell parents to return if children had a fever, and to reinforce the message that LLINs should be used every night) might have been anticipated. Coverage of RTS,S was higher among children using LLINs than among children not using LLINs but a substantial proportion of children not using LLINs did receive RTS,S, thereby increasing the proportion of children with access to some form of malaria prevention. The increase was especially marked in Ghana and Malawi where, in the midline surveys, LLIN use was lower than in Kenya.

Several factors could have diluted estimates of safety signals. However, the fact that the impact observed against severe malaria was consistent with the expected impact, and the consistent point estimates for other impact outcomes, argue against dilution effects having been substantially underestimated. Confounding, whereby malaria vaccine uptake is associated with underlying risks of malaria, meningitis, or mortality, could influence estimates of effects. However, we found no association between EPI coverage and malaria prevalence during baseline surveys. With respect to meningitis, although children who received the malaria vaccine were more likely to have previously received pneumococcal vaccine and *H influenzae* type B vaccine than children who did not receive the malaria vaccine, this was unlikely to have masked an effect of the malaria vaccine on meningitis risk because vaccine serotypes of *H influenzae* type B and pneumococcus were relatively uncommon when CSF samples were investigated by PCR. For cerebral malaria, confidence intervals were wide and there was more uncertainty due to the difficulty of excluding other causes of encephalopathy in the sentinel hospitals. Monitoring for this and other safety signals continues to the end of the evaluation.

Despite efforts to strengthen clinical investigation, diagnostic performance was imperfect and events could have been missed or misclassified.^20^ It is also likely that deaths have been under-reported through the mortality surveillance system. However, the analytical approach aimed to control for differences in completeness of investigation and detection, and for imbalance in access to hospital by using data on the same outcomes in non-eligible age groups in each cluster as an auxiliary variable. In addition, in the case of meningitis, the sensitivity analysis using imputed values suggested no bias due to missing lumbar puncture results. The analytic approach avoided reliance on person-time denominators, which can be difficult to estimate reliably (especially for hospital outcomes for which catchment populations and access to hospital might be poorly defined), and improved statistical efficiency compared with standard statistical methods for outcomes with highly variable counts among clusters.^10^

This evaluation shows that RTS,S can be effectively deployed through national immunisation programmes with no evidence of the safety signals that had been reported from the phase 3 trial, leading to substantial reductions in severe illness caused by malaria and mortality reductions. Although now recommended by WHO, supply of the RTS,S vaccine is forecast to fall far short of demand for the next 4–6 years.^21^ Our findings highlight the urgency to increase supplies of this or other safe and effective malaria vaccines, and to ensure resources to introduce and implement malaria vaccines effectively.

For more on the **European Medicines Agency on the vaccine** see https://www.ema.europa.eu/en/opinion-medicine-use-outside-EU/human/mosquirix

## Data sharing

Anonymised data will be made available through Harvard Dataverse. Requests for access will be reviewed by a data access committee.

## Declaration of interests
