# Supplementary material for: Feasibility, safety, and impact of the RTS,S/AS01E malaria vaccine when implemented through national immunisation programmes: evaluation of cluster-randomised introduction of the vaccine in Ghana, Kenya, and Malawi
Source: Lancet. 2024 Apr 27;403(10437):1660–70. doi: 10.1016/S0140-6736(24)00004-7 (PMC11718159; doi:10.1016/S0140-6736(24)00004-7)
Supplement: Supplementary appendix [file mmc1.pdf]

THE LANCET

Supplementary appendix

This appendix formed part of the original submission and has been peer reviewed. We post it as supplied by the authors.

Supplement to: Asante KP, Mathanga DP, Milligan P, et al. Feasibility, safety, and impact of the RTS,S/AS01E malaria vaccine when implemented through national immunisation programmes: evaluation of cluster-randomised introduction of the vaccine in Ghana, Kenya, and Malawi. *Lancet* 2024; published online April 4. https://doi.org/10.1016/S0140-6736(24)00004-7.

Supplementary materials document for manuscript entitled “Feasibility, safety, and impact of the RTS,S/AS01$_E$ malaria vaccine when implemented through national immunisation programmes: evaluation of cluster-randomized introduction of the vaccine in Ghana, Kenya and Malawi”.

Table of contents

1 Identification of countries and evaluation partners for the pilot introductions. 7
1.1 Country selection....7
1.2 Pilot area selection 7
1.3 Evaluation partner selection 7
2 Randomization 7
3 Details of methods 8
3.1 Household surveys 8
3.1.1 Ghana 9
3.1.2 Kenya 11
3.1.3 Malawi 15
3.2 Sentinel hospital surveillance....17
3.2.1 Ghana 17
3.2.2 Kenya 19
3.2.3 Malawi 20
3.2.4 Reference laboratory testing of CSF samples....22
3.3 Community Mortality Surveillance methods 23
3.3.1 Ghana 23
3.3.2 Kenya 24
3.3.3 Malawi 26
4 Vaccine introduction....27
5 Data management and statistical analysis....28
5.1 Data management....28
5.2 Statistical methods....28
5.2.1 Analysis approach....28
5.2.2 Power calculations 31
5.2.3 Comparison of rate ratios for safety outcomes with safety signals from the phase 3 trial: ...32
6 Ethical considerations....35
6.1 Consent....35
6.2 Implied consent for vaccine implementation 36
6.3 Role of the Sponsor....36
7 References....36
8 Supplementary tables....37
9 Supplementary figures 53

Table of tables

Table S1: Survey dates in each country......37
Table S2: Case definitions......38
Table S3: Data management processes......40
Table S5 Reasons why lumbar punctures were not done, amongst children with suspected meningitis......42
Table S6 Sensitivity analysis for meningitis......42
Table S7 Pathogen of confirmed meningitis cases......44
Table S8 IRR among children eligible for at least one dose of RTS,S/AS01: safety outcomes......46
Table S9 IRR among children eligible to have received three doses of RTS,S/AS01: safety outcomes......48
Table S10 IRR among children eligible for at least one dose of RTS,S/AS01: impact outcomes......50
Table S11 IRR among children eligible to have received three doses of RTS,S/AS01: impact outcomes......51
Table S12 Sensitivity analysis including cases from outside the pre-specified sentinel areas......52

List of figures

Figure S1A - C. Maps of evaluation areas....53
Figure S2 Vaccination schedule for RTSS and childhood immunization schedule....57
Figure S3 Parameters of age-eligible and age–ineligible populations by country. 58
Figure S4 Vaccine Coverage and childhood interventions by country, restricted to sentinel hospital area59
Figure S5 Hospital admissions flowchart....60
Figure S5 Hospital admissions flowchart. 60
Figure S6 Flowchart showing deaths included in the analyses. 62
Figure S7 Probable or Confirmed Meningitis Analytic Population from Hospital Admissions 63
Figure S8 Cerebral Malaria (CM) Analytic Population from Hospital Admissions 64
Figure S9 Severe Malaria Analytic Population from Hospital Admissions 65

Abbreviations

AVAREF	African Vaccine Regulatory Forum
BDH	Balaka District Hospital, Malawi
BPP	Basic Paediatric Protocol (BPP
CHAs	Community Health Assistants
CHO	Community Health Officers
CHS	Community Health Strategy
CHU	Community Health Unit
CHVs	Community health volunteers
CIN	Clinical Information **Network**
CKIs	Community Key Informants
CMAM	Community-Based Management of Acute Malnutrition
CRF	Case Report Forms
CSF	Cerebrospinal fluid
DHS	Demographic Health Surveys (DHS)
DPT	Diphtheria-pertussis-tetanus
DPT-HepB-Hib 3	Diphtheria-pertussis-tetanus-Hepatitis B-Haemophilus influenza-b dose 3)
DSMB	Data Safety Monitoring Board
DTP	Diptheria Tatanus Pertusis vaccine
DVACs	District Verbal Autopsy Coordinators
EA	Enumeration area
EPI	Expanded Programme on Immunization
EQA	External quality assessments
ETAT+	Emergency Triage Assessment and Treatment Plus
GCP	Good Clinical Practice
GHS	Ghana Health Service
GSS	Ghana Statistical Service
HBR	home-based record
ICF	informed consent form
ID	Identification
IEC	Information education and communication
IRB	Institutional review board
IRS	Indoor residual spraying
ITT	Intention to treat analysis
KEMRI	Kenya Medical Research Institute
KNBS	Kenyan National Bureau of Statistic
LCHV	Lead community health volunteers
LLIN	Long lasting Insecticide treated nets
LP	Lumbar puncture

LSHTM	London School of Hygiene & Tropical Medicine
MDH	Machinga District Hospital, Malawi
MICS	Multi-Indicator Cluster Surveys
MIS	Malaria Indicator Surveys
MR1	Measles-Rubella 1
MR2	Measles-Rubella 2
mRDT	mala rapid diagnostic test
MUAC	Mid upper arm circumference
MVIP	Malaria vaccine implementation programme
MVPE	Malaria Vaccine Pilot Evaluation
NDH	Ntchisi District Hospital, Malawi
NICD	National Institute for Communicable Diseases, South Africa
NRB	National Registration Bureau
ODK	Open data tool kit
P. falciparum	*Plasmodium falciparum*
PAR	Paediatric admission record
PCR	Polymerase chain reaction
PCV	Pneumococcal conjugate vaccines
PFPR	*Plasmodium falciparum* prevalence (PFPR)
RNA	Ribonucleic acid
RR	Rate ratio
RVACs	Regional verbal autopsy coordinator
SAS	Survey Analysis Package
SD	Standard deviations
UNICEF	United Nations Children's Fund
USSD	Unstructured Supplementary Service Data
VA	Verbal Autopsy
VH	Village heads
VRs	Village reporters
WHO	World Health Organisation
WHO ERC	WHO Ethic Review Committee
WHO PAG	WHO Program Advisory Group,

1 Identification of countries and evaluation partners for the pilot introductions.

1.1 Country selection

WHO initiated the country selection process by issuing a call for expressions of interest addressed to Ministries of Health in sub-Saharan Africa in December 2015. Of the 10 countries that responded, 3 were selected for the Programme based on pre-specified criteria. Key among these criteria was the desire to engage in the MVIP by national stakeholders – particularly the Ministry of Health – and well-functioning malaria and immunization programmes. Other criteria included: good coverage of recommended malaria control interventions and childhood vaccinations; moderate-to-high malaria transmission despite good implementation of WHO-recommended malaria interventions; a sufficient number of infants living in the malaria-transmission areas where the vaccine will be introduced; strong implementation research or evaluation experience in the country; and capacity to assess safety outcomes. Participation in the Phase 3 RTS,S/AS01 trial was an additional criterion considered during the country selection process. Visits to the three shortlisted countries took place at the end of 2016 and in Q1 of 2017 to discuss the proposed Programme in more detail and confirm countries' interest in taking part.

The Ministries of Health of Ghana, Kenya and Malawi were officially informed about their selection in February 2017. The names were made public on 24 April 2017.

1.2 Pilot area selection

In-country stakeholders in all three countries provided recommendations on the most suitable sub-national areas for the pilot based on pre-set criteria by WHO. This included identification of areas with malaria prevalence of 20% or higher to reduce the chances that continued progress in control rendered pilot areas low transmission settings during the course of the pilot. The selection process was finalized in line with local considerations by the Ministry of Healths.

1.3 Evaluation partner selection

WHO published a request for proposals in May 2017 to identify prospective partners to conduct the pilot evaluations in each country. In each country, a consortium of partners consisting of highly competent and experienced institutions with strong local connections were identified to conduct the household surveys, establish sentinel hospital surveillance and the community-based mortality surveillance. Agreements between WHO and the lead partner of each of the three consortia were signed in September/October 2018. Sentinel hospitals were selected through country-led processes. Based on the master protocol, which was approved by the WHO Ethics Review Committee in February 2018 (with an amendment approved in October 2018), country-specific protocols were developed and submitted for ethics review. Evaluation activities began in 2019, following ethical approvals for the protocols by all required ethics committees.

2 Randomization

The cluster randomized approach was used to achieve three objectives 1) to ensure that each area had an equal chance of receiving the vaccine, 2) to provide the best chance that characteristics of implementation and comparison areas were balanced at baseline, and 3) randomization provides the strongest basis to infer that differences between areas with and without vaccine could be attributed to the vaccine.

The units of randomisation were administrative areas (typically sub-counties or districts) and referred to as clusters (Figure S1A-C). To ensure that implementation and comparator clusters were similar at baseline a balanced, or constrained, randomization was conducted using the administrative data.

Factors considered in the balanced randomization included:

- The ratio of the total population of surviving under 1 year olds was set not to exceed 1.1 (or be below 1/1.1). Similarly, the ratio of the standard deviation of the populations was set not to exceed 1.1 (or fall below 1/1.1).

- Modelled *Plasmodium falciparum* prevalence (PFPR) by cluster. Clusters were stratified in each country into tertiles. The difference in the number of clusters in each tertile assigned to implemention versus comparison was set not to exceed two in each country
- The ratio between implementing and comparator clusters of the population vaccinated with DTP /pentavalent dose 1, DTP /pentavalent dose 3 and Measles vaccine dose 1 was set not to exceed 1.1 (or fall below 1/1.1) for each of the vaccines. The population vaccinated was calculated as the population in the cluster multiplied by the coverage of that vaccination (up to a maximum of 100%).
- The number of clusters with sentinel hospitals was set to be the same in implementation and in non-implementation areas. The ratio of the total number of hospital admissions in implementation and non-implementation was set not be above 1.1 (or below 1/1.1). For each sentinel hospital the ratio of hospital admissions from implementation areas vs non-implementation was set not be above 3.5 (or below 1/3.5). "Sentinel areas" were defined by those clusters feeding into the sentinel hospitals.
- The ratio of the total number of each type of health facility (hospitals, health centres and dispensaries) between implementation and non-implementation areas was set not to be above 1.1 (or below 1/1.1)
- In Kenya and Malawi, the countries requested an additional criterion to ensure further administrative level representation. This meant that in each county or district respectively, there should be at least one cluster in one group and one in the other group, (i.e within a county or district, not all clusters should be in the same group). This criterion was not requested nor applied in Ghana as the administrative level representation was based on the region and this criterion was already met.

With support of WHO, a computer programme was developed to provide a long list of acceptable permutations for cluster allocation, with each assigned a unique, sequential number. Once the list of options was produced for each country, a linkage analysis was performed to check that an adequate set of balanced options was accurate. This included checking that balance criteria were not overly constraining and, for example, forcing that some clusters were always - or never - allocated together. Once this was confirmed the list of balanced options was provided to the country so that one option could be selected. In each country, pieces of paper, each with the number of one of the allocation options, were folded and placed in a container. One of the pieces of paper was pulled out of the container by the designated individual at the country's stakeholder event.

3 Details of methods

3.1 Household surveys

Primary feasibility objectives

- To estimate the proportion of children aged 12–23 months who had received three doses of RTS,S/ASO1 by 12 months of age (midline survey), and the proportion of children aged 27-38 months who had received their fourth dose of RTS,S/AS01 by 27 months of age (endline survey)

Secondary feasibility objectives

- To estimate the coverage of recommended EPI vaccines in children from areas implementing RTS,S/AS01 and in children from areas not implementing RTS,S/AS01.

- To estimate the proportions of children receiving each individual dose (the first, second, third, fourth, as appropriate) for each recommended vaccine

- To estimate the coverage and utilization of ITN/LLIN, IRS and any other recommended malaria prevention and control measures, in children from areas implementing RTS,S/AS01 and in children from areas not implementing RTS,S/AS01.
- To document patterns of health-seeking behaviour for febrile children among children from areas implementing RTS,S/AS01 and in children from areas not implementing RTS,S/AS01.
- To assess if the introduction of additional contacts between 5-9 months of age alters drop-out rates for routine vaccinations and changes the number of fully vaccinated children.
- To assess whether the introduction of RTS,S/AS01 is associated with a change in the coverage of other key childhood interventions, including anti-helminth administration (deworming) and Vitamin A supplementation.
- To investigate the agreement between malaria vaccine status by caregiver recall, from the home-based record, and from immunization registers, in order to understand the validity of RTS,S/AS01 status from different sources.
- To assess changes in malnutrition as measured by MUAC score following the introduction of RTS,S/AS01.

Table S1 describes the dates for each survey in each country

3.1.1 Ghana

The household surveys took place in a total of 66 clusters in the study area. At the baseline survey, four enumeration areas (EAs) from each cluster were then randomly selected by the Ghana Statistical Service (GHS) to represent a cluster in the MVPE. The midline survey was conducted in the same EAs used for the baseline survey.

In each sampled EA, an experienced supervisor visited the EA and subdivided the EA into 4 segments using maps provided by the GSS. Each segment was allocated a number, and the supervisor randomly selected one segment using a computer program on the tablets for data collection. All households within a selected segment of an EA were visited to identify and interview all eligible children. The households were then marked to prevent multiple visits to the same house after the interview had been conducted. The marking was also used for quality control visit checks by study supervisors and monitors.

In each household, mothers/caregivers of eligible children aged 5-48 months were interviewed. At midline, we placed more emphasis on reaching out to children aged 12-23 months. In the absence of the mother, an adult member of the household who could provide the required information was interviewed. If a caregiver or an eligible child was absent in a household at the time of the visit, the interviewer made revisits to the household. If a caregiver or eligible child was still absent after three planned revisits, the data collector took note of the household and reported to the supervisor. Furthermore, if the mother was absent and there was no any adult member of the household who could provide the required information, the interviewer moved to the next household.

Questionnaire

A survey questionnaire was used to collect information on the background characteristics of the participants aged 5-48 months, their households, the vaccines received and characteristics of the household's dwelling

unit, such as source of water, type of toilet facilities, materials used for the floor of the dwelling unit, and ownership of various durable goods. The questionnaire covered the following topics:

- Background characteristics of household, respondent and eligible children
- Vaccination card status, and the dates the children received RTS, S vaccines and other routine EPI Vaccines
- Coverage of Malaria Control Measures (ITN/LLIN, IRS)
- Utilization of Malaria Control Measures (ITN/LLIN)
- Household socio-economic status (Household assets)
- Patterns of Health seeking behaviors for febrile children
- Access to Health facilities/Services
- Other childhood interventions (deworming and Vitamin A supplementation)

Community sensitization

Prior to the start of data collection for each survey, community members and opinion leaders such as assembly members were sensitized on the survey activities. Letters were also written to the regional health directorates and the districts assemblies of the districts in the pilot implementation areas to notify them of the survey and solicit their support for the conduct of the survey. The Ghana Health Service through the Expanded Programme on Immunization (EPI) led the community sensitization efforts.

Training of field staff

Field staff for each survey were trained prior to data collection over a five day period. Due to the coronavirus pandemic, the field staff were divided into two groups for the data collection training to ensure social distancing. The training was facilitated by researchers from the consortium and the Ghana Statistical Service. The training included presentations on various topics by the researchers.

- Overview of the Malaria Vaccine Pilot Evaluation (MVPE)
- Overview and purpose of the feasibility module
- Study design and sampling procedures
- Roles and responsibilities of interviewers and field supervisors
- Vaccination data collection using maternal recall

The training course also included instructions on interviewing techniques and field procedures, a detailed review of questionnaire content, instruction on how to administer the paper and electronic questionnaires. There were also mock interviews between trainees to gain the required skills in asking questions. The interviewers were trained on how to administer and obtain informed consent with several role plays between them on obtaining informed consent. All the interviewers and supervisors were trained in malaria testing using rapid diagnostic tests (RDTs) and how to take blood samples and placed on a paper (blood spot) for secondary testing. They were also taught how to measure the Mid-Upper arm circumference (MUAC).

Pretesting of the survey questionnaire were done at each survey in communities near the training venue which were not included in the sampled EAs for the midline survey. There were debriefing sessions after the field practice sessions which were used to provide clarifications to trainees and made modifications to the questionnaires based on lessons learned during the conduct of the pretest of the data collection tools in the field. After the training, 84 participants were selected to serve as interviewers and 21 as supervisors. Selection of supervisors was based on their experience leading survey teams and their performance during the training.

Field Organization

Data collection was carried out by 21 field teams, and each team consisted of one supervisor, four interviewers and one driver. Each team was assigned a number of EAs in a region. Researchers from the consortium and some senior staff from GSS coordinated and monitored fieldwork activities. The project

principal investigator was responsible for the overall implementation of the survey, and supported by the feasibility team. Staff of the GSS provided technical assistance in the conduct of the survey.

To facilitate communication and logistics supply among the field teams, WhatsApp groups were created for the entire survey team, the coordinators and each of the 3 regional teams. Additionally, coordinators were also in constant communication with individual data collectors and field supervisors via phone. Survey coordinators worked with a central coordinating team at KHRC to facilitate, supervise and coordinate smooth implementation of the survey. The IT team provided support and clarification on issues related to the data capturing system. Each region had a coordinator who was the immediate contact person for the field team if there were problems to be resolved or issues to be clarified. As part of supervision and monitoring, the researchers and coordinators made visits to the field teams to observe interviewers at work or confirm that the interviews had been done.

3.1.2 Kenya

The household surveys took place in a total of 46 sub-counties (MVPE clusters) in the study area. The baseline survey aimed to describe baseline characteristic of the MVPE areas and population, and targeted households with children 5–48 months of age. The midline survey aimed to estimate the coverage of RTS,S/AS01 doses 1 through 3 and targeted households with children 12–23 months of age. The survey methodology remained consistent throughout the feasibility evaluation, with any differences between the baseline and midline surveys described below. All surveys were carried out in the same 184 enumeration areas (four EAs per MVPE cluster).

Sampling

For the baseline survey, we used a two-stage cluster design: four enumeration areas (EA) were selected by the Kenya National Bureau of Statistics (KNBS) according to probability proportional to the estimated population size in each sub-county; 25 households were targeted to be randomly selected from each EA (without replacement), resulting in a targeted 100 households per sub-county with a known, non-zero probability of selection. After selection of the EAs in each sub-county, mapping teams (study team leads) were trained on enumeration procedures and carried out mapping ahead of the survey, first by identifying the boundaries of the EA based on the household census maps received from the KNBS office, and then by enumerating of all households within the EA boundaries. At the time of mapping, teams identified households within each EA that have eligible children residing in them. All households within an EA with eligible children constituted the sampling frame, and from this sampling frame 35 households were randomly selected, with an aim to complete interviews in approximately 25 of them. All consenting mothers/caretakers of children aged five months to 48 months living in the household were eligible for participation. Information was collected for each eligible child.

For the midline survey, we carried out the enumeration as described above, with households enumerated in all 184 EAs. However, all households with children 12–23 months of age (not a sub-sample) participated in the survey, essentially resulting in the census of households with 12–23 month olds.

Questionnaires

The quantitative variables in the feasibility analysis were drawn from the standard questionnaires used in Demographic Health Surveys (DHS), Malaria Indicator Surveys (MIS), and Multi-Indicator Cluster Surveys (MICS), with addition of any variables relevant to the RTS,S/AS01 vaccine or MVPE overall. Harmonizing the survey tools with those developed and tested for DHS, MIS and MICS enhances the comparability and interpretation of the results across the surveys, while building on the previous and well-established research. To enable electronic data entry on tablet computers, the software programming (ODK) and data processing took place well ahead of training and field activities to allow for testing of the data

entry tools. Survey questionnaires were translated into Kiswahili, Dholuo, Maragoli and Bukusu languages that, in addition to English, are widely spoken in western Kenya.

Household information domain

The household information domain includes the geographic coordinates of the household, household census, demographic data, and socio-economic status that will be used for sample description and stratified analyses. Malaria control measures (ITN, IRS) are also a part of this domain and will provide malaria intervention coverage estimates over time and with introduction of the RTS,S/AS01 vaccine.

Mother/caretaker domain

The mother/caretaker domain includes demographic descriptors of mothers/caretakers, birth history, and childhood mortality questions.

Child health domain

The child health domain contains ITN use, immunization history (by immunization card and/or maternal/caretaker recall), other childhood public health interventions (deworming and vitamin A supplementation), health seeking behaviors for fever and other symptoms, hospital admissions, malaria treatment, and a nutritional assessment by measuring mid-upper arm circumference. Immunization history was assessed by asking mothers to show the Mother and Child Health Booklet or health card used for recording childhood immunizations for each child of eligible age. If the booklet or the card is available, dates for all immunizations were recorded and a photo of the card was taken. If a vaccination was not recorded in the booklet or card, the interviewer asked the mother to recall whether the vaccination was given. If the booklet or card is not available, the mother/caretaker was asked to recall each vaccination and number of doses given. We also asked mothers to recall vaccinations even if the health card was available for the assessment of the verbal recall by the caregiver to enable the comparison between the written record and the recall and, therefore, allow for the validation of the verbal recall in this specific population.
Malnutrition was assessed through a mid-upper arm circumference (MUAC). Children that are found to be malnourished were given a referral to the nearest health facility for nutrition assessment and care, per Kenya National Guidelines for Integrated Management of Acute Malnutrition (e.g., essential medical treatment such as antibiotics, vitamin A, iron, and folic acid supplementation, and special nutrition products; http://nak.or.ke/wpcontent/uploads/2017/12/Kenya-MoH-IMAM-Guideline-June-2009.pdf).

Vaccine acceptability domain

The vaccine acceptability domain includes assessment of perception of malaria importance as a health issues, conditional RTS,S/AS01 vaccine acceptability at baseline and over time, as well as barriers and facilitators of the vaccine uptake. Questionnaires were adapted from the Knowledge, Attitudes, and Practices (KAP) surveys previously implemented for cholera, measles, flu and other vaccines in Kenya and other countries, with the inclusion of questions based on the Health Utilization Study (qualitative). The acceptability assessment provides an indication of population readiness for the vaccine at baseline. Monitoring acceptability over time in the comparison areas will provide a reflection of the vaccine perceptions and the success of the implementation. Information on barriers and facilitators will provide programmatically-relevant information at the community level with trends over time, in conjunction with the more in-depth qualitative research (Health Utilization Study) carried out under a different protocol.

Malaria testing

Finger prick blood samples were collected in the baseline and midline household surveys to enable detection of *Plasmodium spp. infection* using RDTs. This provided the baseline malaria prevalence across the implementation and comparison areas. Where the results of the malaria RDT were positive, antimalarial treatment was offered in accordance with the national guidelines and national IRB requirements.

Informed consent

Before participation in the feasibility surveys, all potential participants were provided with information about the survey as contained in the informed consent form. We clearly communicated that participation in the study is voluntary, and that a participant may withdraw at any time. Participants who could not provide written informed consent, were consented orally in the presence of an independent witness who signed the consent form, in addition to finger print of the participant. Consent was obtained directly from all mothers/caretakers participating in the study for the household and mother/caretaker modules of the questionnaire, and additionally for each of the child of eligible age for the child modules of the questionnaire and for malaria testing, treatment, and referral for severe malaria treatment (as needed). Mothers who are <18 years old, per Kenya national guidelines for HIV counselling and testing, are considered "mature minors" and are able to consent for participation for themselves and their children. All consent forms will be translated into Dholuo, Maragoli, Bukusu, and Kiswahili and back translated into English to ensure accuracy. Confidentiality was maintained throughout the research process and information will not be shared with non-study staff.

All data and other information are maintained confidentially to the greatest extent possible. The names and house numbers will be kept strictly confidential and not appear on any reports or associated with the results in anyway or available to anyone except the survey and other staff involved.

Community sensitization

Before the baseline and midline surveys, we carried out extensive community sensitization activities. In addition to the sensitization meetings harmonized with other MVPE components and MoH/EPI/MVIP programs, we met with the county and sub-county administrative and health branches leadership to introduce the surveys and outline the timelines and activities. After the enumeration areas where the survey took place (4 per sub-county) were selected by the Kenyan National Bureau of Statistics, we met with the chiefs, assistant chiefs, village elders, community health volunteers, and other local leadership from each selected enumeration area to inform them about the survey objectives, methods, activities and dates and use other community communication venues (e.g., chief's barazas) to provide information directly to the community members. We contacted the local officials again before the start of enumeration and data collection activities in each enumeration area.

Training of field staff

The baseline survey closely followed the methodology employed by other routinely conducted surveys in Kenya (i.e., DHS, MIS, MICS), in consultation with the KNBS. Training of team supervisors, interviewers, monitors, quality assurance staff, and reserve staff took place over two weeks, in line with the DHS curriculum. At the end of the training, the questionnaires were be piloted in the field and the translations will be reviewed based on the field experience.

- Overview of the Malaria Vaccine Pilot Evaluation (MVPE)
- Overview of the feasibility evaluation
- Study design and sampling procedures
- Roles and responsibilities of interviewers and field supervisors
- Vaccination data collection using maternal recall
- Interviewing techniques and field work procedures
- Safety of field work
- Research ethics and consenting (including practice)
- Study questionnaires and tablet proficiency (including practice)
- Geospatial orientation and use of GPS, satellite imagery, and Google Earth for enumeration and household location
- MUAC training
- Laboratory training on administration of malaria RDTs

Field-testing of enumeration and the survey questionnaires was carried out in communities that were not included in the sampled EAs for the surveys, with a debriefing to answer questions or address different situations following the field testing.

Training for the baseline survey was done concurrently for the entire field team. However, the midline survey took place during the COVID-19 pandemic and adjustments had to be made to minimize the COVID-19 risk to staff during the training. As a result, the training was carried out in in a staggered manner, with the teams from the same hub cohorted for the training and for all subsequent field work. In this scenario, each hub (4 teams) trained separately, with the second hub starting the training once the first hub has moved out to do field testing and data collection, and the third hub following the departure of the second hub. While this training structure resulted in a delayed start (by 5 days and 10 days, for the second and third hub, respectively) of data collection and strained the human resources (trainers and on-site supervision), it was deemed as affective in both preparation for the survey data collection and keeping the study staff safe.

Survey administration

Prior to the survey, participating villages / enumeration areas were visited and local administration was notified about the start of the data collection. All consenting primary caretakers/mothers of children of eligible age were interviewed. An interview was conducted for each eligible child, thus some mothers were asked/ interviewed more than once.

Data collection work took place in July through October 2019 (13 weeks) for the baseline survey and May through June 2021 (8 weeks) for the midline survey. Twelve teams, each responsible for ~4 sub-counties (~15 EAs) and comprised of a team lead and community interviewers (trained in data collection, malaria testing, treatment and referral) were recruited and allocated according to language competency across the pilot implementation area in western Kenya. Details of the assigned EAs and sampled households were provided by the field teams and members of the mapping team, as well as a village guide, which enabled the teams to properly identify the households. A minimum of two additional callbacks were made where eligible respondents are absent. Each team spent three days in each EA, with the remaining time allocated to tracing the households unavailable during the initial visit. Three field supervisors and three data monitors were based at the regional hubs covering northern, central, and southern regions. They carried out daily visits to the EAs to monitor the field activities and provide oversight. Evaluation leadership (i.e., field coordinator, study coordinator, and study investigators) provided regular supervision as well by visiting the data collection teams in the field.

Data management and analysis

Data were captured electronically by using tablet computers. Entered questionnaires were checked for missing data and inconsistencies by the data monitor upon completion, then encrypted data were transferred to a cloud server, with local back-up. Data monitors based at the regional hubs carried out the second level of data quality control by performing additional checks for anomalies and missing data, timely correction, and submitting the high quality data into the central database.

All analyses were weighted and adjusted for intra-cluster correlation. We calculated the weights based on the population of each EA, non-response adjustment, and probability of selection of the houses participating in the survey. Descriptive statistical and spatial analyses using survey analysis package (SAS v9.14) estimated the immunization coverage, barriers and facilitators, health seeking behaviors, hospitalizations, and other indicators of interest. The outcomes specified in primary and secondary objectives were compared between the implementation and control areas and over time.

In both surveys, the caregivers were asked to provide a recall on immunizations for their children regardless of whether the immunization card (or home-based record [HBR]) was available. Caregiver recall was ascertained using two questions: 1) has the child received the vaccine? 2) if yes, how many doses? Doses

from HBR were as recorded in the vaccination books; doses for recall were extrapolated based on the number of doses recalled (e.g., any child with ≥1 dose is "yes" for dose 1; any child with ≥2 doses is "yes" for dose 2, etc.). The analysis evaluating the agreement between the caregiver recall and home-based records was carried out on a subset of children for whom both HBR and recall was provided. Percent agreement, Brennan-Prediger, and Gwet's AC1 coefficients were used to calculate the agreement between HBR and caregiver recall. Agreement is considered reasonably good if >0.70. Analyses were stratified by intervention and comparator areas.

Quality control

We utilized several quality control mechanisms embedded in the household surveys and routinely implemented as part of DHS. Three members of the field team are specifically responsible for on-going quality control: team leads, data monitors, and field supervisors. Team leads observe interviewer performance with the aim of improving and maintaining quality of the collected data. This is done by regular interview observation, verifying the consent forms, and continuous review of issues with the interviewers. Field supervisors oversee the overall quality of data collection, assist field monitors with end of the day verifications, and conduct periodic re-interviews for spot-checking the information for selected households. Data monitors check data for completeness, and address any issues revealed through the automated daily monitoring report. Questionnaires were geo-coded and time-stamped, allowing for an additional dimension of quality control of field activities. Data quality control was implemented through validation rules in data entry software, as well as quality assurance staff who are responsible for checking data for missing values, outliers, anomalies, and inconsistencies, and communicated back with the field supervisor for timely corrections. Daily reports were generated to assess the quality of the submitted data, looking at the key missing variables, duration of the interviews, duplicate IDs, and other quality control indicators to both inform the field supervisors and the team leads about improvements that need to be made in data collection practices and to rectify any data issues in a timely manner.

3.1.3 Malawi

The household surveys were conducted in nine (9) districts across Malawi which were partitioned into 46 clusters of at least 100,000 people per cluster with approximately 4,000 children aged 5-48 months. The districts were selected based on the intensity of malaria transmission and the burden of malaria disease. In all these districts, malaria transmission is intense (P. falciparum parasite prevalence > 20%) and year-round, peaking during the rainy season (November through May). Different enumeration areas were randomly selected for the baseline and midline surveys.

Sampling

A two-stage cluster design sampling was used to select 100 and 140 households in each cluster in the baseline and midline survey respectively. Forty-six (46) clusters, each with a population of at least 100,000 people and an estimated population of 4,000 children aged 5-48 months, were created in the nine districts. In each cluster, four (4) enumeration areas (EAs) were selected based on probability proportional to estimated population size (PPS). By definition, an EA is a census track with an average population of 1200 people. The number of households in EAs, however, ranges from 235 to 1000 households.

A total of 184 EAs were selected and a household listing activity was conducted by trained local enumerators in all the selected EAs based on maps provided by the National Statistics Office (NSO). Using a tablet-based tool, all households with at least one eligible child aged between 5 months and 48 months served as the sampling frame within that EA for the second stage. In the week before the baseline survey, a fixed number of 35 households with children aged 5-48 months were randomly selected in each EA and data collected from the first 25 responding households. In the midline survey, a fixed number of 45 households per EA were randomly selected from the created household listing of households with an eligible child aged 5-48 months old. To ensure that each household has an equal probability of selection,

prior to the survey a statistician used a simple random sampling to select 45 households from the sampling frame in each EA. The field survey team collected data from the first 35 households that we present in each of the selected EAs. After interviewing the first 35 household, no further interviews we done in the EA. The survey team re-visited the households twice if the household owners were not present the first time. In both surveys, all children aged 5-48 months in the household were enrolled and their caregivers interviewed.

Questionnaire

A questionnaire, adapted from the standard Malawi Demographic and Health Survey (DHS) questionnaires and malaria indicator surveys (MIS), was used to collect information on household demographics and socio-economic characteristics, basic demographics for the child caregivers of eligible children, malaria control interventions such as ownership and use of malaria interventions (e.g. LLINs, IRS), child health information (dates for vaccinations, dates and types of other non-vaccine public health child interventions such as Vitamin A supplementation and deworming and recent illnesses such as fever) and health seeking behaviors as well as treatments received. Information on vaccinations received for each child was also verbally obtained from the child's caregiver. A photograph of the child's health passport was taken and linked with the child's information to facilitate validation of vaccinations received and dates. Where no child's health passport was available, the vaccination information was only obtained from the child's caregiver.

Measurements

Malaria testing: Finger prick blood samples were collected in the baseline household surveys to enable detection of Plasmodium spp. infection using RDTs (Bioline ®). This provided the baseline malaria prevalence across the implementation and comparison areas. Where the results of the malaria RDT were positive, antimalarial treatment was offered in accordance with the national guidelines and national IRB requirements.

Anthropometry: In both surveys, mid-upper arm circumference (MUAC) of each enrolled child was measured using MUAC tapes. Children that were found to be malnourished based on MUAC measurement were given a referral note to the nearest health facility for further nutrition assessment and care, as per Malawi Guidelines for Community-Based Management of Acute Malnutrition (CMAM).

Community sensitization

The MVPE team worked with district health officers to plan and conduct the sensitization meetings with traditional leaders and the community to explain the purpose and procedures of the survey as well as to obtain verbal community consent. Once permission was granted, household listing and field survey teams staff started working in the community to list households, map the households and conduct the survey.

Training of survey team, piloting and field work

All household survey team members received a five-days training on: study protocol, questionnaire content, field procedures and use of electronic data collection tools, participants' consenting and interviewing which included role play interviews and consenting between participants in the classroom, EA mapping, household listing and data management including quality assurance, and quality SOPs. During the training, survey team participants were evaluated through pre and post-tests, quizzes and observations during training and field practice. The training was delivered through lectures (power point presentations), demonstrations of MUAC measurements using MUAC tapes.

The selection of field supervisors was based on their previous experience in leading survey teams and their performance during the pre-post evaluation tests as well as primary training. Field supervisors received an additional one day of training on performance of supervisory activities which included backing-up the data, assigning households, troubleshooting common problems encountered in the field, identifying and addressing common errors in data collection, uploading collected data to central repository, communication

with field team members and central office team, re-charging the tablets and batteries, conduct of data quality control activities and field supervision.

Following the training, a pilot survey was held in Blantyre district. Blantyre district is not one of the districts implementing the MVPE. A full day field practice provided the field team with more hands-on practice before the real fieldwork. Nearby EAs were purposively selected so that each field team had a chance to go and practice in areas such as EA mapping, household listing, administration of the questionnaire, data back-up, uploading and synchronizing data with the central repository and collection of blood samples. After the field pilot exercise, a debriefing session was conducted with the survey field teams. Modifications to the questionnaires were made based on lessons from the training exercise.

Data management and analysis

All electronic data collected during the surveys were uploaded to the central server in Blantyre, where the data were stored and cleaned. Coverage of vaccines and malaria interventions in the malaria vaccine intervention and control clusters were estimated using descriptive statistical analyses. Weighted proportions and corresponding 95% confidence intervals or means with standard deviations (SDs) were estimated for the different indicators to take into account the clustered nature of the survey data. Specifically, descriptive statistics were used to describe the key indicators such as vaccine coverage and timeliness of vaccinations such as for diphtheria-pertussis-tetanus-Hepatitis B-Haemophilus influenza-b dose 3 (DPT-HepB-Hib 3) as well as measles-Rubella 1 (MR1) and MR2. Comparisons of the key indicators between malaria vaccine implementation and control areas were based on the confidence intervals, and these were considered statistically significant if they did not overlap.

3.2 Sentinel hospital surveillance

3.2.1 Ghana

Across the previous Brong Ahafo (now Bono, Bono East and Ahafo regions), Central and Volta (now Volta and Oti), a series of up to 8 sentinel hospitals serving both implementation and comparison clusters were identified, split between areas implementing and comparison areas to provide hospital-based data for safety and cause-specific impact assessments. The hospitals were purposively selected based on their ability to conduct LPs, geographical location and through consensus with the Ghana Health Service.

Enhancing clinical capacity of sentinel hospitals and quality systems

The selected hospitals within the study area did not have all the capacities required for this study. WHO provided support to improve capacities such that the selected hospitals were fully operational before RTS,S/AS01 introduction. The deficiencies' identified during hospital selection were discussed with the clinical Institutional Care Directorate which provided support to the identified hospitals. This includes ensuring provision of basic laboratory tools such as rapid diagnostic tests, and kits for CSF assessments in collaboration with WHO. In addition, the laboratories were enrolled into an external quality assessments system for malaria microscopy which is coordinated by the Ghana Health Service Clinical Laboratory Division in collaboration with Kintampo Health Research Centre. Clinical coordinators (nurses or physician assistants) are located in each sentinel hospital to identify, consent and extract relevant patient data to meet the study endpoints definitions (Table S2). The coordinators were supervised by clinical monitors (paediatricians and laboratory scientists) and the sentinel hospital focal persons. The clinical monitors provide daily support to the clinical coordinators through phone calls and WhatsApp messages without any patient data. Face to face onsite visits are made to each participating hospital on monthly basis the clinical monitors and laboratory scientists.

To develop capacity and ensure standardised data collection, a clinical and laboratory training module was developed in collaboration with WHO. In addition, a job aid was developed to guide clinicians in the selected hospitals in their case evaluation based on the clinical algorithms developed by WHO for this program and reporting. The training was led by Prof. Daniel Ansong (Professor of Child Health, School of Medical Sciences, Kwame Nkrumah University of Science & Technology and Deputy Director, Research and Development Unit, Komfo Anokye Teaching Hospital) and selected paediatricians and Laboratory scientists. The training is repeated on yearly basis to maintain the capacity of existing personnel and standardise the capacity of new personnel.

Clinical quality systems in sentinel hospitals

The quality of clinical data were assessed for completeness and compliance to the clinical algorithms on daily basis by the clinical coordinator, pediatricians and laboratory scientists.

Identification of participants

All eligible children (1-59 months) admitted to sentinel hospitals whose parents or caregivers give consent are enrolled into the evaluation by the sentinel hospital clinical coordinator. The clinical coordinator records the routine demographic data such as age, date of admission, sex, normal place of residence, current place of residence if this is different, laboratory results, diagnoses, treatment and outcome of admissions (dead, alive, referred). At discharge, the final diagnoses are recorded per the ICD 10 codes as done within the Ghana Health Service and by the National Health Insurance Scheme. In addition, project specific data such as date of birth, vaccinations history are collected. A photograph of the health card may be taken and stored with the child's CRF to facilitate validation of vaccinations and dates. Where no health card is available the information is solicited from the caregiver through verbal recall (no dates) and documented as such. When vaccination information is collected through maternal recall, the caregiver/mother is asked about each vaccine (per country-specific EPI guidelines) and the number of doses, with detailed prompts characterizing the vaccines to enhance the quality of the recall (e.g., describing oral polio vaccine as bitter drops; etc.). In a subset of cases, parents are asked additional questions to validate maternal recall. Where a vaccine record or maternal recall do not provide information on the vaccine status, a review of the national vaccine register is done to ascertain the vaccination status of the child. Children are managed according to national treatment guidelines.

Identifying cases with indication for Lumbar puncture (LP): An algorithm of symptoms was developed to identify potential patients who require LPs among all patients in the age group of interest. LPs are performed according the national guidelines. Where LPs are not done when indicated as per the algorithm, the clinical coordinator recorded why not done.

Consent for sample storage: As part of the study, participants have the option to decide during the consenting processes if they would agree to have their samples stored for future research in meningitis and malaria. The option to withdraw their consent without giving any reason at any time for sample storage is explained to participants.

Obtaining informed consent

All eligible children (1-59 months) admitted to sentinel hospitals whose parents or care-givers gave consent were enrolled into the safety evaluation by the sentinel hospital clinical coordinator. A log of all persons approached is maintained to document the proportion of consent obtained and approved. If consent is refused parents or care-givers are informed that there would be no negative consequences for their child and they will still receive the usual standard of care at the sentinel hospital or any other hospital. The Ghana Health Service Ethical Review Committee encourages the use of one informed consent form (ICF) per participant and hence a joint ICF was used to document consent for the study related activities.

3.2.2 Kenya

The safety evaluation consists of surveillance at sentinel hospitals and started after ethical approval before introduction of the vaccine. Sentinel surveillance will continue for 12 months after 4th dose (total duration of surveillance is 46 months after start of vaccinations and 49 from start of surveillance). All children aged 1-59 months admitted to the hospitals contribute data for the safety surveillance. Hospital-based surveillance (embedded in the CIN network[1]) systematically documents all admissions to the pediatric ward in order to capture information on the safety profile of RTS,S/AS01. The assessment is done using a standard pediatric admission record (PAR) and children with suspected meningitis and cerebral malaria who require lumbar puncture and CSF sample storage are identified at the time of admission assessment.

Kenya's CIN network, to which all 6 sentinel hospitals belong, serves three purposes, among others: a) improve documentation and use of routine inpatient data, and b) to improve uptake and monitor compliance of recommended inpatient care through regular audit and feedback to hospital teams on various processes of care for common illnesses and 24 hospitals are currently involved in Kenya. The collaboration promotes use of Kenyan Government inpatient pediatric guidelines, referred to as Basic Paediatric Protocol (BPP), which is a locally adapted version of WHO pocket-book guidelines for common illnesses. The BPP booklets are provided to the hospitals by the research team, and management (clinical assessment, investigations, and treatment) of common illnesses are expected to be performed in accordance with clinical algorithms in the booklet by the hospitals' clinical teams. Job aides detailing clinical management algorithms are also printed and supplied to the hospitals. Clinical staff working in pediatric areas receive yearly Emergency Triage Assessment and Treatment Plus (ETAT+) training, which include clinical algorithms outlined in the Basic Paediatric Protocol (BPP). Refresher trainings are also facilitated locally by the pediatrician when necessary.

CIN collaboration has successfully developed and implemented three standard medical forms as part of the hospitals' medical records system: a paediatric admission record (PAR) for standardised documentation of admission patient history, admission clinical assessment, and investigations done at admission; a treatment form for documentation of immediate treatment for admitted patients ; and a discharge form for documenting patient's final disposition (dead, alive, referred, absconded), discharge treatments, clinical status at discharge, and follow-up plan. The hospitals have a trained data clerk employed by the project who abstracts data from medical records immediately a patient is discharged. CIN feedback reports on process measures for care for common illnesses are prepared and sent to each hospital every three months. During sentinel surveillance, weekly and monthly feedback reports on compliance with MVPE clinical algorithm were sent to the study clinician and the hospital pediatrician. Auto-generated R scripts are used to prepare the feedback reports. Other CIN activities include network meetings involving key hospital personnel (paediatricians, nurse-in-charge of paediatrics, laboratory in-charge, and health record offices) and telephone discussions with paediatricians to promote completeness of documentation and use of standard medical forms. The clinicians, employed by the hospitals, are in charge of the children's care and are responsible for initiating treatment according to standard practice and national guidelines. The CIN system was adapted to support the sentinel evaluation. This involved recruitment of a dedicated study clinician (non-physician clinician/clinical officer), supporting the hospitals with supplies to support consistent investigation of meningitis, malaria, and severe malaria. The study clinician also acted as a local facilitator to support compliance with surveillance algorithm and was responsible for consenting.

Informed consent for the collection of sentinel hospital surveillance data

Data required for evaluation are part of routine data collected during medical care and are part of routine medical notes by the CIN protocol, which is approved by KEMRI SERU. This include use of de-identified before storage and use therefore patients are not consented individually. CIN protocol has been approved by the KEMRI SERU, which has approved consent waiver for use of data. However, individual consent was taken short term storage, shipment, and molecular testing at the reference laboratory. A separate individual consent was also taken for long-term storage of CSF samples. A protocol amendment was

obtained in February 2021 to allow for waiver of individual consent for short-term storage and testing at the reference laboratory for participants where a CSF sample was taken but the patient died before consenting could be done. Consent waiver was requested to avoid causing emotional distress to families in such few cases where death occurs before consenting for CSF storage was done. Any future use of the samples, not currently described in the protocol, will require independent ethics approval by KEMRI SERU.

When lumbar punctures are performed in children with suspected meningitis or impaired consciousness, the child's caregiver is approached by the study clinician (clinical officer) after the lumbar puncture procedure is done for consent for laboratory personnel to separate an aliquot of CSF (0.5ml) to be frozen at -20ºC and stored at the in-country laboratories in Kisumu (-80°C) for shipping and molecular testing for pathogens at a reference laboratory. Performance of lumbar puncture does not require study research consenting because it is done only when clinically indicated in accordance with national guidelines (BPP) to guide clinical care. Clinicians performing lumbar punctures use local hospital procedures for obtaining permission for routine clinical procedures and these involve explaining any clinical procedure or treatment to the patient or parent/caregiver. Parents/caregiver can refuse to have lumbar puncture procedure, like any other clinical procedure, and is such cases the clinician follow local procedures for documenting a refusal of a routine clinical procedure/treatment.

3.2.3 Malawi

Although the vaccine is distributed across 9 districts, only 4 district hospitals serve as sentinel hospitals for the safety surveillance module. These hospitals are Balaka District Hospital (BDH), Machinga District Hospital (MDH), Mchinji District Hospital (MCDH) and Ntchisi District Hospital (NDH). Balaka and Machinga district hospitals are located in the southern region of Malawi, serving a population of 450,000 and 500,000 respectively. Mchinji and Ntchisi district hospitals are in the central region of Malawi and have a population of 630,000 and 320,000 respectively. The pediatric admissions to these hospitals range from 2500 – 5000 children annually.

Data were collected from children aged 1 to 59 months hospitalized (over 24 hours) in the children`s wards of the sentinel hospitals with a medical illness. Patients were enrolled consecutively on hospitalisation. Relevant demographic, clinical and vaccination data was captured on Case Report Forms (CRFs) from both the implementation and comparison regions. RTSS vaccination sand vaccination status of all Expanded Program for Immunisation (EPI) was also recorded for all admissions.

Capacity Building of the sentinel site hospitals

The selected hospitals within the study area did not have all the capacities required for this study. WHO provided support to improve capacities such that the selected hospitals were fully operational before RTS,S/AS01 introduction. The following activities were undertaken to improve the ability to collect all the necessary information:

Staffing: On commencement of data collection in April and May 2019, each sentinel hospital had team comprising of 2 nurses, 1 administrator and 1 district manager. Since April 2020, 1 MVPE clinical officer was hired for each sentinel hospital, primarily to optimize the adherence to the clinical algorithms and improve lumbar puncture rates for the sentinel hospitals. Locum clinical officers were also put in place to be available for the times the MVPE clinical officers needed to go on leave.

Malaria Rapid Diagnostic tests: On commencement of the data collection, the diagnosis of malaria was based on either a blood film for malaria parasites, a blood rapid diagnostic test (MRDT) or both. Following recommendations from the WHO Program Advisory Group, from March 2021 a system was implemented to use mRDTs routinely for the diagnosis of malaria for children hospitalized in the sentinel hospitals under the surveillance data collection.

Haemoglobin measurements: Over the first half of the data collection, the team relied on the haemoglobin measurements using the sentinel hospital laboratory (full blood count) and ward HemoCue® machines . Challenges were noted in the capacity of having a haemoglobin results available at optimal rates due to resource limitations of the sentinel hospitals. Following this, HemoCue® machines were obtained by the MVPE team for use in the hospital surveillance when indicated per clinical algorithm. These hemocue machines for haemoglobin measurement were introduced to be used at all, times when it was not possible to measure haemoglobin in the hospitals due to resource limitations. This HemoCue ® system support was implemented in in April 2021.

Laboratory support: All laboratories in the sentinel sites were fitted with solar backup to mitigate frequent power blackouts. The MVPE also provided reagents to support in the districts in diagnosis of malaria and meningitis. CSF samples are shipped to reference laboratories outside Malawi to provide preliminary diagnosis of meningitis. The MVPE hospital surveillance microbiologist supported the sentinel hospitals in their laboratories. This included bi-annual external quality assessments (EQA), training support on the gaps noted during these visits and laboratory reagent support (for CSF analysis)

Training: Regular training of health workers on in inpatient management algorithms to assess case definitions in a standardised way, was provided to health workers quarterly by the MVPE experts. Other than the quarterly meetings, the following trainings have also been delivered:

- An initial central protocol training for all investigators and hospital surveillance staff (University of Malawi College of Medicine and University of North Carolina Project Malawi) was performed in March 2019. This was followed by a protocol and standard operating procedure (SOP) training in each sentinel hospital. These sentinel hospital specific trainings included the Ministry of Health staff per hospital, prior to commencement of data collection over April and May 2019.
- Annual protocol and SOP trainings are scheduled for the MVPE hospital surveillance staff. Bi-annual trainings are scheduled with the sentinel hospital clinical staff (on clinical algorithms and laboratory microbiology processes including EQA).
- Monthly meetings occur between the MVPE district supervisors, the MVPE sentinel hospital key personnel, laboratory manager/representative and hospital leadership on the monthly metrics for hospital admissions, lumbar puncture rates, laboratory results and other parameters that are key to the MVPE hospital surveillance and how these may be improved.

The surveillance continued to allow for 12 months follow up after the children vaccinated during the first year of vaccine introduction have received dose 4. This will provide 12 months follow-up after dose 4 assuming that the first dose of RTS,S/AS01 is given by around 5 months of age, the third dose is delivered by age 9 months and the fourth dose is given by age 27 months. A 12 month follow up period after dose 4 brings children to 39 months of age. However, admitted children up to 59 months of age will be included in the evaluation for practical simplicity (data collection up to under the age of 5 years), to enable documentation of critical events in children who are vaccinated near the beginning of the programme, possible extended data analysis and also because events in children too young or old to receive RTS,S/AS01 provide useful information about underlying rates in the same cluster. Areas of interest on data collection will be occurrence of malaria and meningitis.

Quality assurance procedures: The open data tool kit (ODK) used for electronic data entry has inbuilt logic checks that query if data entered seems incorrect (e.g. in the case a date of birth translated to a child who is in an ineligible age band at that time). The ODK logic also prompts for a lumbar puncture when eligible criteria per clinical symptoms or examination findings entered. On a weekly basis over Malawi sentinel hospital surveillance teleconferences, the hospital surveillance teams discuss data reports (raised by the data management team) to discuss trends, challenges and possible solutions for correction and the prevention of

the recurrence of the same errors. These teleconferences are attended by all sentinel hospital staff, the principal investigator and the program management team.

3.2.4 Reference laboratory testing of CSF samples

CSF samples from the sentinel hospitals were analysed in two reference laboratories; Medical Research Council (MRC), Gambia (for samples from Ghana) and the National Institute for Communicable Diseases (NICD), South Africa (for samples from Kenya and Malawi).

PCR assays for Haemophilus influenzae, Neisseria meningitidis, and Streptococcus pneumoniae detection:
MRC Gambia: Monoplex real-time PCR assay targeting hpd (H. influenzae), sodC (N. meningitidis), and lytA (S. pneumoniae) genes was performed on all CSF samples. PCR positive samples for H. influenzae and N. meningitidis were then subjected to monoplex real-time PCR serotyping. For samples that are positive for S. pneumoniae, genomic DNA was extracted from the CSF samples followed by serotyping using multiplex real-time PCR.

NICD: Pathogen detection was done using multiplex real-time PCR assay targeting hpd (H. influenzae), ctrA (N. meningitidis), and lytA (S. pneumoniae) genes(1) and an ABI 7500 Fast real-time PCR instrument (Applied Biosystems). Real-time PCR serotyping was performed on all samples that tested PCR positive.(2)

PCR assays for Enteroviruses detection
MRC Gambia: RNA was first extracted from the CSF samples and the samples were subsequently subjected to reverse transcriptase multiplex real-time PCR with an intrinsic control which tests assay efficiency.
NICD: PCR assay for Enterovirus detection was performed according to specifications using the commercial kit RealStar® Enterovirus RT-PCR Kit 1.0 (Altona Diagnostics, Hamburg, Germany).

3.3 Community Mortality Surveillance methods

3.3.1 Ghana

Children contributing to the impact evaluation comprised vaccinated and unvaccinated children living in the RTS,S pilot introduction areas aged 1 to 59 months. The surveillance period was planned for a period of 46 months to provide 12 months of surveillance data after children vaccinated during the first year of the programme receive their fourth vaccine dose, assuming that the first dose of RTS,S/AS01 is given as soon as possible after 6 months of age, second dose at 7 months, that the third dose is given by 9 months of age, and the fourth dose is given by age 24 months. A 12-month surveillance period after dose 4 brings children to 39 months of age.

Data were collected in children aged up to 59 months to enable documentation of any critical events that might occur in the longer term after vaccination in children who are vaccinated at the beginning of the programme. Vaccination with RTS,S vaccine was expected to continue beyond the evaluation period, unless data showed a negative change in the benefit risk profile of the vaccine or the EPI programme decides to no longer implement the vaccine. To facilitate operational activities and minimise the risk of excluding relevant events due to inaccuracies in initial reporting of age in young children in these settings, information was collected on children reported to have died between 1 to 59 months. The data for those too young or old for RTS,S/AS01 provides important information about underlying rates of outcomes in the same cluster.

The Community Mortality Surveillance commenced on the 1st May 2019, a day after implementation of the RTSS Vaccine was launched. The surveillance is being implemented in three regions at the time: Volta region, Central region and former Brong Ahafo region. A total of 66 clusters (districts) from the three regions contribute to the evaluation. At the community level, the assistance of community leaders, community members and Community Health Officers (CHO) was sought to notify Community Key Informants (CKIs) about any death in a child under 5 years of age. A wider age group than that for vaccine during the pilot period is included in the surveillance. This minimises the risk of missing an event due to errors in the dates, and the data for children too young or too old to receive RTS,S/AS01 provide important information about background rate of mortality in the same cluster. When the CKI is notified s/he visits the family as soon as possible to confirm the event and complete a paper CRF (Fact of Death notification). CKIs report the deaths to District Verbal Autopsy Coordinators (DVACs) by submitting completed paper CRFs or by notification via phone call. CHOs in the Community-based Health and Planning Services (CHPS) zones assist CKIs to report deaths to the DVACs especially CKIs who have challenges reaching the DVAC. Each DVAC uses a Samsung galaxy tablet (installed with REDCap) to capture data electronically for the MVPE. As a strategy to ascertain completeness of death reporting, DVACs also pick deaths at Sentinel Hospitals. These deaths are followed up to the community level to confirm whether they have already been picked by CKIs or not. If it is confirmed that the death is not already picked by the CKI and is eligible, then the DVAC obtains consent and conducts the verbal autopsy. The purpose of picking deaths from the health facility is to compare with deaths picked at the community level to ensure completeness of reporting. Each Sentinel Hospital has a Mortality Register where all eligible deaths are recorded. For each eligible death captured in the Mortality Register, a Health Facility paper CRF is completed for onward entry into the MVPE database. The Health Facility CRF is designed differently from the CRF the CKI use so as to differentiate between the two. When the DVAC is notified on any death 1 to 59 months, they enter information of the diseased in a death notification form in an MVPE database built on REDCap. The REDCap contains three main interphases: death notification interphases, VA interphase and pictures/reports interphase. After entering information of the diseased child in the death notification interphase on REDCap, the DVAC then follows up to the household (of the diseased) to obtain informed consent and conduct a Verbal Autopsy. At the household, it is the parent/caregiver of the diseased child

that grants the consent and Verbal Autopsy interview. The Verbal Autopsy is conducted electronically using the WHO 2016 verbal autopsy instrument built on REDCap. Verbal Autopsies are conducted on all deaths 1 to 59 months.

The Community Mortality Surveillance also has Regional Verbal Autopsy Coordinators (RVACs) who supervise the DVACs. As part of quality control, the RVACs carry out supportive supervision by observing the DVACs conduct some interviews. Through this, feedback is given to enhance the quality of interviews and consenting. Also, the RVACs conduct quality control re-interviews on randomly generated Verbal Autopsies each month. A project manager oversees implementation of the Community Mortality Surveillance while the Co-PI for the impact module provides oversight direction.

All persons assigned on the project have been trained accordingly. CKIs have been trained by the routine health system to report adverse events following public health interventions such as vaccinations in the communities using a community register or by verbal history. As part of the malaria vaccine evaluation, CKIs in the MVPE area were further trained on death picking and reporting using the paper CRF with emphasis on the rationale for mortality surveillance in the context of this study and community engagement process. The Project Manager, RVACs and DVACs were also trained on the evaluation protocol and procedures with emphasis on the rationale for mortality surveillance in the context of this study, community engagement and consenting according to Good Clinical Practices. In addition, they were trained on verbal autopsy interviews using the WHO VA questionnaire.

As part of quality control measures, all CRFs are reviewed before submitted to the database. District VA Coordinators review all paper CRFs received from CKIs before entries are made on REDCap. In addition, when these paper CRFs are submitted to the Regional offices, they are further reviewed by the Regional VA Coordinators. All Verbal Autopsies conducted by the DVACs are electronically reviewed by the RVACs on REDCap. Quality control checks also include RVACs conducting partial repeat interviews and accompanied interviews. Partial repeat interviews are conducted on a randomly selected proportion of all Verbal Autopsy interviews conducted in a month. This is to verify that the original interview took place, and to double-check the responses to key questions in those interviews. Accompanied interviews involve RVACs witnessing standard interviews to ensure the approach of the interviewer is appropriate and that responses to questions are elicited in a sensitive, acceptable and comparable way per study protocol. Performance data is reviewed monthly and quarterly. Those clusters identified as outliers, either because of increased or decreased reporting rates, are prioritised and followed-up with targeted monitoring. Where a vaccine record or maternal recall do not provide information on the vaccine status, the potential to seek information from the clinic registers for deaths, in vaccine-eligible age groups in RTSS clusters was considered where feasible.

3.3.2 Kenya

The MoH, through the Community Health Strategy (CHS), utilizes community health volunteers (CHVs) to conduct community mortality surveillance for all deaths of children under the age of 5 years. CHVs are supported and managed by Community Health Assistants (CHAs). One or more CHAs are assigned to a Community Health Unit (CHU), which is comprised of about 10 CHVs and a catchment population of approximately 5,000 (500-1000 households). One CHV (lead CHV [LCHV]) in a CHU informally coordinates the activities of the rest of the CHVs. Community mortality surveillance as conducted by the Kenya Malaria Vaccine Programme Evaluation (MVPE) Consortium leverages this existing system.

Upon confirming that a death has occurred in the community, the CHV informs the LCHV. The LCHV visits the household of the deceased at a culturally appropriate time frame (approximately two weeks after

the event) to fill a locator form (LF) and sends an Unstructured Supplementary Service Data (USSD) to the MVPE data center. The USSD reports the date and place of death, and the LCHV captures additional data, including the date of birth, date and place of death, age at death, sex, place of normal residence, RTS,S vaccination status, parent/guardian name and contact details. Within two weeks, the LCHV delivers the LF to a MVPE designated CHA who verifies the entries for completeness and accuracy, scans the LF image using ScanForm technology, which automatically digitizes the data and converts it to electronic database which is instantly available to the data manager. The data manager identifies deaths which are eligible for VA (based on age at death and location of residence) and informs, via email, the field team who organize for a VA to be conducted by a CHA at the home of the deceased child between one and three months after the date of death.

VAs have been conducted since late September 2019 for all children aged 5-48 months at the time of death. At the time of VA, the CHA administers written informed consent and conducts a VA using a questionnaire programmed on a tablet that was adapted from the 2016 version of the WHO VA questionnaire for children aged 28 days to 11 years. The data from the VA is transmitted to a cloud-server at the time the VA is complete, if network is available, or later, when internet connectivity is available. In areas where MVPE overlaps with other studies that conduct VA in western Kenya (e.g. CHAMPS and HDSS) the MVPE team coordinates with these studies to ensure that VAs are not conducted for a death more than once. Data are shared between studies using an approved data sharing agreement. Probable cause of death is assigned using the WHO-recommended approach; the analytical software InterVA-5 model.

Mortality Surveillance Staffing and training

Initially, between October through early November 2019, MVPE core staff trained the County Community Strategy focal person, the sub-County focal persons and two MVPE designated CHAs in each of the 46 sub-counties included in MVPE. This team was responsible for training an additional 611 CHAs who were then tasked, under supervision by the core MVPE staff, with the training of >14,000 CHVs in the CHUs they supported. The trainings were conducted simultaneously across the 46 sub-counties. The training content included, study overview, the importance of mortality monitoring in general, need to understand the causes of death in order to prioritize action to intervene and to assess the impact of new measures to improve survival, vaccine safety principles, content and rationale for standard operating procedures, locally appropriate ways to inquire about deaths, supervisory structure, reporting forms and mechanisms, and methods for conducting active surveillance for deaths at households. Practical training on the use of USSD was also conducted. Pre-training and post-training assessments were conducted for all CHVs and only 61% (9,007/14,766) of them could correctly fill and send a USSD. It was also noted that data management from the huge number of CHVs was challenging. Based on these findings we opted to change strategies in November 2019.

Through the new strategy, we opted to train the LCHVs (total 1478, one per CHU) to perform death notification via USSD and to fill the LFs. The rest of the CHVs would notify their respective LCHV of all eligible deaths in their villages. A total of 96 MVPE designated CHAs (two per sub-County) were trained on how to conduct VA using the standardized WHO VA interviewer curriculum. Additionally, the CHAs were trained on the study protocol and Good Clinical Practice (GCP). This strategy has been implemented since late November 2019.

Additionally, we found that the MVPE area was not fully covered by established and functioning CHUs. Working directly with the County and sub-County Ministries of Health we have managed to initiate another 99 CHUs. We continue to increase the number of CHUs as additional CHUs are formed and larger CHUs are divided.

On March 13, 2020 the Kenya Ministry of Health and Kenya Medical Research Institute (KEMRI) instituted guidelines to mitigate the transmission of COVID-19. These guidelines prohibited the conduct of

all but essential services being provided to study participants. Gatherings were banned and CHV visits to households were no longer allowed. Our CHVs continued to gather information of deaths, and alert the LCHVs who submitted USSD messages and filled LFs without visiting the households, but VAs could not be conducted. Additionally, while we planned to conduct quarterly refresher trainings for the LCHVs, this was not possible due to the restrictions. In October 2020, we received approval from the KEMRI Scientific and Ethics Review Unit to resume verbal autopsies and trainings. In December 2020, we trained 21 new CHAs who either replaced those that had been posted out of MVPE areas or to fill the gaps of newly established CHUs. At this time we also conducted refresher training (meetings at CHU level) for CHVs from poorly performing CHUs and held several virtual consultative meetings with the CHAs.

3.3.3 Malawi

Community and facility mortality surveillance

Following the commencement of RTSs vaccination in Malawi in April 2019, mortality surveillance system was initiated and progressively scaled up from 1st May to October 2019 in 46 clusters spread across approximately 11,000 villages in 9 districts According to the Malawi National Registration Act of 2010, village headmen (VHs), assisted by their clerks, are required to register community deaths using National Registration Bureau (NRB) community death registration forms (NR10-C) while clinicians or nurses are expected to register deaths occurring in health facilities using NRB facility death registration forms (NR10). For this project, mortality data collection is implemented by teams comprising of full-time research evaluation assistants (who oversee data collection in each cluster), village heads (VH), village reporters (VRs) (who operate as community key informants) and clinicians or nurses working in health facilities. For this project, There were 51 EAs to ensure that each cluster had 1-2 EAs and approximately 2784 VRs, each covering 3-4 villages. The VHs and their clerks, VRs and clinicians received formal death registration training before commencing data collection.

When an under-5 death occurs at a health facility, trained clinicians or nurses complete the NR-10 forms by interviewing the close relatives of the deceased and/or extracting relevant notes from the health facility records. When an under-5 death occurs in the community, family members or VRs report the deaths to the VHs who, with the support of their clerks, complete NR10-C forms and also record the particulars of the deceased (including their names, date of birth, age, date and place of death, and place of residence) into village death registers. Since vaccination status is not routinely captured during the death registration process, the EAs visit homes of caregivers of children who die in health facilities to administer a vaccination status questionnaire. The EAs also perform verbal autopsies with caregivers of children who die in the community, within 14-28 days of death occurrence. They use the 2016 WHO verbal autopsy instrument which includes questions on the vaccination status to the deceased child. Written consent is sought from caregivers before conducting verbal autopsies (VAs) or administering the questionnaire. VRs support the EAs in scheduling VAs with family members of the deceased children and guiding them to their homes on the day of the VA.

Each VR is expected to meet with the VH weekly to ensure accurate documentation of the deaths. The EAs are expected to conduct 4-8 weekly visits to VHs, VRs and health facility-based clinicians and nurses providing them with mentorship and extracting data from NRB death registration forms into ODK-programmed tablets. Key players involved in community mortality surveillance team (EAs, VHs and their clerk, VRs) conduct quarterly meetings to review the success and challenges associated with implementing community death registration and verbal autopsies and agree on action points to improve the efficiency and quality of the community death surveillance system. Government-employed community health workers are invited to attend these meeting to check the consistency of data collected through the community death registration with available Ministry of Health community-based data routinely collected in village health registers.

4 Vaccine introduction

Following the cluster allocation process, the introduction and ongoing vaccine delivery in the vaccination areas begun under the responsibility of the National Immunization Programme in each country. GSK Biologicals registered and obtained authorisation from national regulatory authorities to use RTS,S under the trade name Mosquirix ®. Orders for the vaccines were made by WHO based on EPI logistics estimates receipt from each country through UNICEF. The vaccine is administered to different age ranges in each country. The first dose of RTSS is administered at 5 months of age in Malawi; and at 6 to 7 and 6 to 11 months inclusive in Ghana and Kenya, respectively (figure 2)

Publicity and social mobilisation: A plan was developed by National Task Force sub-committee for advocacy, communication, and social mobilization. There was high level stakeholder engagement at the national, regional district and the community level. These include media and spokesperson training; media engagement (press briefing, workshops, media monitoring, etc.); development of information education and communication (IEC) materials; design and production of radio jingles.

Capacity building and Training: The national EPI guidelines and training modules were revised to include the RTS,S vaccine. Health workers were trained at all levels of health delivery on topics such as rationale for the pilot implementation, vaccine preparation and delivery, the RTSS vaccination schedule, recording of the vaccine in the passport or card, health messages to give caregivers and strategies to ensure children receive all four doses, and surveillance for adverse events following immunizations.

Data Management, Monitoring and Evaluation: Design and review of all data monitoring tools including the child health records, immunization tally sheets, monthly performance and reporting form to include RTS,S/AS01 vaccine administered. National, regional and district monitoring teams were formed to monitor and supervise the implementation activities.

Logistics and waste management: A cold chain needs assessment was conducted to decide on cold storage capacity at all levels to ensure a successful implementation of the vaccine. Another significant activity undertaken was the rehabilitation of existing incinerators and the construction of new ones to improve waste management system to ensure appropriate disposal.

5 Data management and statistical analysis

5.1 Data management

Data sources include household surveys data conducted prior to and approximately 18 months after the RTS,S vaccine introduction, sentinel hospital and mortality data surveillance data. The data was collected using ODK or similar tools in each country (Ghana, Kenya, and Malawi). Country teams managed the data primarily for completeness, range and consistency checks. Data was transmitted to London School of Hygiene and Tropical Medicine (LSHTM) using agreed secured platforms. Additional quality checks were preformed by at LSTMH in collaboration with investigators in each country. Table S3 below describes the processes adopted.

5.2 Statistical methods

5.2.1 Analysis approach

Hospital and community mortality surveillance was maintained for children aged 1-59 months. This includes age groups eligible to receive RTS,S/AS01 vaccine, children who were too old to receive the vaccine, and children too young to have had their first dose. We take advantage of the data in non-eligible age groups to improve estimates of the incidence rate ratio in vaccine-eligible age groups. This adjusts for imbalance between the intervention and comparison areas, an important advantage when outcomes are detected in hospital and clusters are large relative to hospital catchments as constrained randomization may not be able to achieve good balance. A further advantage in our study is that reliance on person-time denominators, which can be challenging to estimate reliably, is avoided. The approach can also mitigate some of the loss of power arising from the clustered design. This is comparable to the use of paired data, for example the use of baseline data on the incidence of the outcome collected for a period prior to intervention in all clusters, for the same purpose of improving power and controlling confounding. A limitation of our approach is that indirect effects (i.e. intervention effects on the level of transmission) are not captured. However, this is not an issue for the safety outcomes, and for malaria outcomes any indirect effects of RTS,S/AS01 implementation in young children are likely to be negligible.

In each cluster, children aged 1-59 months who are admitted to hospital, and children reported to have died, can be classified into eligible or non-eligible groups. Classification into eligible (group A) and non-eligible (group B) is based on the age at admission or death and the date of RTS,S/AS01 introduction, not on whether they actually received RTS,S, so the classification can be applied in exactly the same way in comparison and implementation clusters (see Figure S3).

The rate ratios can be estimated using the double ratio of counts estimator as follows. We denote the incidence rate in eligible age groups in implementation areas by n_{1A}/T_{1A}, where n_{1A} is the number of events and T_{1A} the person time. The rate in comparison areas is n_{0A}/T_{0A}. The corresponding rates in the non-eligible age groups are n_{1B}/T_{1B} and n_{0B}/T_{0B}. The rate ratio comparing the incidence in eligible age groups between implementation and comparison areas can be estimated by:

$$(n_{1A}/T_{1A})/(n_{0A}/T_{0A}) \text{ x } (n_{0B}/T_{0B})/(n_{1B}/T_{1B}),$$

where $(n_{0B}/T_{0B})/(n_{1B}/T_{1B})$ can be thought of as a correction term. This can be written: $(n_{1A}/n_{1B})\text{x}(n_{0B}/n_{0A})$ x $(T_{0A}/T_{0B})\text{x}(T_{1B}/T_{1A})$. For practical purposes in this study, we assume that $(T_{0A}/T_{0B}) \approx (T_{1A}/T_{0A})$. (While it

is possible to obtain approximate estimates of the population under 5yrs per cluster from census data, the proportion in each age group would be challenging to estimate separately in each area. If we apply the same national estimates of age structure to both areas, the person time term cancels out). The estimate of the rate ratio therefore becomes: $R=(n_{1A}/n_{1B}) / (n_{0A}/n_{0B})$, the ratio of the number of events in eligible to non-eligible age groups in implementation areas, divided by that in comparison areas. In this study we have used a bias-corrected version of the double ratio estimator, R'. Details of the bias correction are given in Ma *et al* (3).

Ma *et al.* (3) show that this estimator (R') of the incidence rate ratio is more efficient than the standard comparison of incidence rates per person time in the eligible age group, when there is a strong correlation between number of events in the eligible and non-eligible groups, and the coefficient of variation in the number of events per cluster is large. It is less efficient when the coefficient of variation is small but importantly it provides a means of adjusting for randomization imbalance. By comparing the relative difference between eligible and non-eligible groups between implementation and comparison areas, we achieve some control for factors such as access to hospital, differences in diagnostic performance of hospitals, and differences in underlying disease burden, which may not be well balanced between intervention and comparison areas, but which are likely to be highly correlated between eligible and non-eligible age groups. With respect to mortality outcomes, the same approach can achieve some control for differences in completeness of mortality surveillance, and underlying mortality rates, between implementation and comparison areas, which are also likely to be highly correlated between eligible and non-eligible age groups.

The value of R' was calculated for each outcome in each country. The variance of log(R') is V(R)x(R/R')2, where V(R) is given by V(R1)+V(R0), where V(R1) and V(R0) are jackknife estimates of the ratios eligible to non-eligible events in implementation and comparison areas respectively. The estimates of log(R') for each country, were combined to give a pooled estimate, $D= \sum log\ R'_i/V(\log R'_i)/\sum 1/V(\log R'_i)$, i=1..3, with variance $V(D)= 1/\sum[1/V(\log R'_i)]$. The pooled rate ratio was calculated as exp(D) and the 100(1-α)% confidence interval given by $\exp[D +/- t_{\alpha/2,C-6} \sqrt{V(D)}]$, with df equal to the total number of clusters C less 2x3=6. To test for interaction by country (Cochran's Q test), $\sum[(D - \log R'_i)^2/V(\log R'_i)]$ is referred to the χ^2 distribution with 2 degrees of freedom.

The female:male mortality ratio in eligible age groups in implementation areas was compared to the female:male mortality ratio in eligible age groups in comparison areas in a similar way. Denoting the total number of deaths in girls in group A in implementation (j=1) and comparison areas (j=0) by $g_{j,A}$, and in boys by $b_{j,A}$, and the corresponding number in group B by $g_{j,B}$ and $b_{j,B}$, the female:male mortality ratio in group A is $R_{j,A}= g_{j,A}/b_{j,A}$ and in group B, $R_{j,B}=g_{j,B}/b_{j,B}$, and the double ratio $(R_{j,A}/R_{j,B})$ is then compared between implementation and comparison areas. $W=\log(R_{1,A}/R_{1,B}) - \log(R_{0,A}/R_{0,B})$, is an estimate of the log of the ratio of the female:male mortality ratios in eligible age groups in implementation areas to that in comparison areas. This is the (log) of the 'r5' estimator of Ma et al. 2022. We have used the bias-corrected version of this estimator. For pooled analysis over the three countries, as before we will have a (bias-corrected) estimate of W for each country, W_1, W_2 and W_3, the combined estimate is then $\overline{W} = \sum W_i/V(W_i)/\sum 1/V(W_i)$, i=1..3, and the variance is $V(\overline{W})= 1/\sum[1/V(W_i)]$. The final ratio of female:male mortality ratios is given by $\exp(\overline{W})$ and the 100(1-α)% confidence interval by $\exp[\overline{W} +/- t_{\alpha/2,C-6} \sqrt{V(\overline{W})}]$, with df equal to the total number of clusters C less 2x3=6. Interaction by country is tested as described above.

We do not explicitly adjust for covariates in these analyses. Randomization was constrained to ensure balance with respect to measured cluster-level covariates, and as explained above the use of data for non-target age groups allows control for confounding with respect to measured and unmeasured cluster-level covariates. We have therefore taken steps to minimize imbalance but it is possible that some confounding remains.

Changes to data and methods since the 2021 WHO report: Preliminary results from this study were reviewed by WHO in 2021(4). The following changes to data and methods were made since the original statistical report: In Ghana the definition of eligibility in our analysis was changed to reflect that children were eligible for their first dose if aged 6 or 7 months (not just at 6 months as previously assumed); deaths occurring in April 2019 have been included (these data were incomplete at the time of the 2021 report); the definition of cerebral malaria was amended after review by an expert panel, to exclude patients with a clear alternative explanation for low conciousness (this resulted in 3 cases being excluded, all of them in non-eligible age groups, one with head injury, one with alcohol intoxication, one with seizure disorder). Two changes to the statistical methods were made based on the findings of Ma *et al.* (3), a) we use the jackknife estimator of variances of ratios, in place of the standard linearised estimator which underestimates the variance, and b) we use bias-corrected estimators for incidence rate ratios. Despite these changes the analytic findings and conclusions remain consistent with those reported in the 2021 report [4].

	**Oct 2021 results**			**Updated estimates**		
	**No. of events in eligible children**			**No. of events in eligible children**		
	**Imple-menting**	**Comp-arison**	**Rate ratio (95%CI)**	**Imple-menting**	**Comp-arison**	**Rate ratio (95%CI)**
**Children eligible for at least one dose of RTS,S (safety)**						
**Meningitis**	27	24	0.81 (0.43,1.55)	28	25	0.63 (0.22,1.79)
**Severe malaria broad**	558	847	0.76 (0.61,0.95)	568	845	0.73 (0.55,0.96)
**Cerebral malaria**	49	54	0.96 (0.61,1.52)	52	56	1.03 (0.61,1.74)
**Other forms of severe malaria**	509	793	0.73 (0.58,0.92)	516	799	0.70 (0.53,0.92)
**Interaction**			1.16 (0.77,1.77)			1.34 (0.80,2.23)
**Severe malaria strict**	450	690	0.71 (0.56,0.91)	459	698	0.69 (0.50,0.94)
**Cerebral malaria**	25	30	0.77 (0.44,1.35)	27	32	0.82 (0.39,1.72)
**Other forms of severe malaria**	425	660	0.70 (0.54,0.89)	432	666	0.66 (0.48,0.90)
**Interaction**			0.94 (0.57,1.56)			1.01 (0.50,2.07)
**Mortality (both sexes)**						
**Mortality, girls**	1060	986	0.98 (0.87,1.09)	1166	1097	0.95 (0.85,1.07)
**Mortality, boys**	1091	1143	0.91 (0.80,1.04)	1220	1265	0.91 (0.80,1.04)
**Interaction**			1.08 (0.93,1.25)			1.03 (0.88,1.21)
**Children eligible for three doses of RTS,S (impact)**						
**Severe malaria broad**	418	689	0.70 (0.54,0.92)	427	697	0.68 (0.49,0.95)
**Severe malaria strict**	342	549	0.65 (0.49,0.86)	333	557	0.63 (0.44,0.91)
**Mortality (both sexes)**	1421	1443	0.93 (0.84,1.03)	1589	1631	0.91 (0.82,1.00)
**Mortality, girls**	691	662	0.98 (0.86,1.10)	766	754	0.93 (0.82,1.05)
**Mortality, boys**	730	781	0.90 (0.78,1.04)	823	877	0.89 (0.78,1.02)
**Interaction**			1.08 (0.92,1.28)			1.02 (0.86,1.21)

Analysis of household surveys: In each country, survey-weighted coverages of key indicators (pentavalent dose 3, measles dose 1, vitamin A within the last 6 months, LLIN use, and full basic vaccination coverage)were estimated amongst children aged 12 to 23 months (inclusive) in both vaccinating and comparison areas in the baseline and midline surveys. Coverage of RTS,S dose 3 was also estimated within

both vaccinating and comparison areas in the midline surveys in all countries amongst children aged 12 to 23 months (inclusive). Vaccination status and receipt of vitamin A in the last six months was determined from home-based records where available, or through caregiver recall where the HBR was not available (recall responses were not collected in the Ghana baseline survey, and Vitamin A coverage was determined from HBR only in both surveys in Ghana, recall only in both surveys in Malawi, and by recall and HBR in both surveys in Kenya). Children were considered vaccinated if the HBR was available and dates were recorded (regardless of the validity of these dates with respect to the timing of doses in relation to the child's age and to other doses), or if the HBR was unavailable and the caregiver reported receipt of the vaccine dose. Children were considered unvaccinated if the HBR was available, but dates were not recorded, or if the HBR was unavailable and the caregiver reported that the child had not received the vaccine dose. Children were excluded from coverage estimates of specific vaccination indicators where the HBR was not available, and the recall response for ever having received a vaccination for the antigen was either missing or 'don't know', or 'Yes' but the response for the number of doses was missing. Children were excluded from coverage estimates of Vitamin A within the last 6 months where the HBR was not available, and the recall response was either missing or 'don't know'. Full vaccination coverage was defined as receiving all doses of the basic EPI vaccines by the time of the survey (BCG at birth, OPV x3 (excluding birth dose), Pentavalent x3, and one dose of measles. Children who received IPV at 14 weeks in place of OPV3 were considered fully vaccinated for Polio (IPV status was only available by recall in Malawi, and not available in Kenya). LLIN use was determined from caregiver recall, and children were excluded from coverage estimates if the response to sleeping under a net last night was either "don't know" or missing.
Vaccine recall questions were asked differently in each country. In Ghana, caregivers were asked if they had received each specific dose (dose 1? y/n; dose 2? y/n; dose 3? y/n). Where a caregiver reported that the child had received a later dose but not received an earlier dose for the same antigen, the later doses were shifted down and counted as if they were the earlier dose (e.g. a child reported to have received Pneumococcal conjugate vaccines (PCV) dose 2 but not dose 1 or 3 is considered to have only PCV dose 1). In Kenya, caregivers were asked if the child had ever been vaccinated against each antigen, and if yes, then asked how many doses they had received. In Malawi, caregivers were asked if the child had ever been vaccinated against each antigen, and then if yes, asked if they had received each specific dose (dose 1? y/n; dose 2? y/n; dose 3? y/n). Where a caregiver reported that the child had received a later dose but not received an earlier dose for the same antigen, the later doses were shifted down and counted as if they were the earlier dose (e.g. a child reported to have received PCV dose 2 but not dose 1 or 3 is considered to have only PCV dose 1).

We accounted for the survey design using survey poisson regression, with sampling weights reflecting the probability of selection into the survey, and response rates, using the survey (svy) commands in Stata. Survey poisson regression was used to estimate coverage, and the relative difference in coverage between the midline and baseline surveys in the vaccination arm adjusting for the same difference in the comparison arm. Survey poisson regression was also used to estimate the association between LLIN use, malaria prevalence, wealth category (split at tertiles of the wealth index), gender, and coverage of dose 3 of RTS,S in the midline survey.

5.2.2 Power calculations

In the phase 3 trial, 21 cases of meningitis occurred in RTS,S/AS01 recipients, a rate of 1.05/1000, and one case in control children, a rate of 0.1/1000; the rate ratio was 10.5 (95%CI 1.41,78.0). There were 43 cases of cerebral malaria in RTS,S/AS01 recipients and 10 cases in control children, a rate ratio of 2.15 (1.1,4.3). There were 67 deaths in girls who received RTS,S/AS01 and 17 in girls in the control group, a mortality ratio of 2, while in boys there were 45 deaths in RTS,S/AS01 recipients and 29 in boys in the control group, mortality ratio 0.8. The relative mortality ratio (girls:boys) was 2.61 (95%CI 1.29,5.26). For safety outcomes, the research question was whether the excess of cases of meningitis and cerebral malaria, and the excess mortality in girls, which were unexplained, were

causally related to the vaccine. We therefore estimated the number of events required for 90% power to detect rate ratios for these safety signals, if they were of the magnitude observed in vaccinated children the phase 3 trial, after allowing for dilution due to vaccine coverage being less than 100%, and allowing for effects of contamination. We also allowed for potential confounding whereby, in the case of meningitis, if RTS,S/AS01 recipients have also received Hib and pneumococcal vaccine, which protect against meningitis, this could to some extent mask a safety signal (in practice this was a small effect due to the fact that vaccine-preventable serotypes were relatively uncommon causes of meningitis). Assuming 5% vaccination coverage in comparison areas, we calculated that the meningitis signal in the phase 3 trial would equate to a rate ratio of 4 if vaccine coverage was 60% in implementation areas, or 5 if vaccine coverage was 70%. Equivalently, the cerebral malaria signal would equate to a rate ratio of 1.7 to 2, and the mortality signal in girls to a mortality ratio of 1.4 to 1.6. (These values were used in the power calculations. More accurate estimates were made later, when data on RTS,S/AS01 coverage from the household surveys was available). Power was estimated using simulations in which the number of events in cluster j and age group k, was a random value from a poisson distribution with mean equal to $r.k_{ij}y_{jkt}\theta_{jk}$, where r is the assumed underlying incidence rate, k_{ij} is an adjustment factor for the relative access to hospital *i* for cluster *j*, y_{jkt} is the person time in cluster *j* in age group *k* at time *t*, and θ_{jk} is the rate ratio for the outcome associated with vaccine introduction, for cluster *j* and age group *k*. $\theta_{jk}=\theta$ for vaccine-eligible age group in clusters in the intervention arm, $\theta_{jk}=1$ otherwise. *kij* represents the relative access to hospital *i* from cluster *j*, *kij*=1 for the cluster in which the hospital is located, 0<*kij*<1 for other clusters in the catchment area, and *kij*=0 for other clusters. *k* values were estimated for each hospital, using data on the number of admissions under 5 yrs from each cluster in the catchment area, for a period before intervention started: *kij*=(*ai*j/*nj*)/(*ai**/*n**), *aij* = admissions to hospital *i* from cluster *j*, *nj*=population in cluster *j*; *ai** is the number of admissions to hospital *i* from the cluster the hospital *i* is located in, and *n** is the population of that cluster. For each simulation, the ratio of the number of events between the two age groups was compared between arms using a ratio estimator. The simulation was repeated 10,000 times, to determine the distribution of the estimate of θ and of the 95% confidence limits. Simulations were done for null value (θ =1) for a range of assumed underlying incidence rates, and for various values of θ >1 (safety) and θ <1 (impact) to estimate power to detect or exclude effects of interest, using estimates of the cluster populations in each age group. Simulations were repeated for a range of values of the underlying incidence rate, for various time points, in order to determine the number of events that would be required to have adequate power, at each time point. We estimated that 90 cases of meningitis and 400 cases of cerebral malaria, in eligible and non-eligible age groups combined, would be required for 90% power, and that 2000 deaths in vaccine-eligible ages would allow 90% power to detect a gender interaction. For impact outcomes, we estimated that a total of 3000 severe malaria cases (eligible and non-eligible groups combined) would be required for 80% power to detect a reduction of 24%, and 4000 for 90% power. Based on event rates observed in the first year of the evaluation we anticipated that the required number of events for each outcome would have accrued by approximately the same time, at about 24 months after the first introduction of the vaccine (April 2021), if data for all three countries were combined.

5.2.3 Comparison of rate ratios for safety outcomes with safety signals from the phase 3 trial:

Data on uptake of RTS,S/AS01 from the coverage surveys undertaken about 18 months after introduction of the vaccine were used to recalculate estimates of dilution factors, these were then used to calculate modelled predictions of the rate ratios that would be expected if the safety signals observed in the phase 3 trial were to occur during the MVIP.

If the safety signals observed in the phase 3 trial occurred in the MVIP, the magnitude of the effect we would observe would be smaller than in the phase 3 trial, since not all children will have received the vaccine. Any effects would be further diluted if there was contamination due to some children in

comparison areas, or children in non-eligible age groups, receiving the vaccine. We used estimates of coverage and timing of malaria vaccine doses from the household surveys in each country to estimate the person time in vaccinated children as a proportion of total person time, and the degree of contamination. These estimates were used to derive predictions of the expected effect in each country, if the safety signals in the phase 3 trial were to occur in the MVIP. The average of these effects for each outcome is shown in column 3 mof the table below, and this is compared with the observed rate ratio from the MVIP (column 4) using a z-test. For meningitis and for the interaction of vaccine impact on mortality by sex, the estimates obtained in the MVIP were inconsistent with the signal in the phase 3 trial, i.e. the hypothesis that the signal observed in the phase 3 trial occurred in the MVIP, given the degree of dilution that was estimated, was rejected ($p<0.05$). For the two definitions of cerebral malaria the corresponding p-values were 0.19 and 0.12.

**Outcome**	**Rate ratio in the phase 3 trial[4] (95%CI)**	**Modelled prediction of the rate ratio if the signal observed in the phase 3 trial were to occur during the MVIP (95%CI)[5]**	**Rate ratio observed in the MVIP (95%CI)**	**z**	**p-value**
**Meningitis**	10.5 (1.41,78.00)	3.67 (1.21,11.1)	0.63 (0.22,1.79)	2.28	0.022
**Cerebral malaria[1]**	2.15 (1.1,4.3)	1.61 (1.05,2.4)	1.03 (0.61,1.74)	1.31	0.19
**Cerebral malaria[2]**		1.59 (1.05,2.4)	0.82 (0.39,1.72)	1.56	0.12
**Relative mortality ratio[3]**	2.61 (1.29,5.26)	1.83 (1.17,2.8)	1.03 (0.88, 1.21)	2.91	0.004

1: Cerebral malaria, using, for MVIP, a case definition including cases in which lumbar puncture had not been performed. 2: Cerebral malaria, MVIP cases in which lumbar puncture had been performed to exclude cases with probable meningitis. 3: The relative mortality ratio, in the phase 3 trial, was defined as the ratio of the mortality rate between vaccine recipients and controls, for girls, relative to that for boys. 4: Rate ratio in the phase 3 trial comparing the combined vaccine groups (R3R and R3R) with the control group, from month 0 to study end.

5: For any outcome, denote the log of the rate ratio comparing the RTS,S and comparator areas in country i by Di, with variance V(Di). The pooled estimate of the log rate ratio, combining the data from the three countries, is: $D_{pooled} = \sum w_i D_i$, i=1..3, where the countries are weighted by the inverse of the variance, weights are $wi = [1/V(D_i)] \div \sum 1/V(D_i)$ and the variance of the pooled estimate is $V(D_{pooled}) = 1/\sum[1/V(D_i)]$. These are the pooled estimates in the main text. Denote the rate ratio for the same outcome from the phase 3 trial by R. From the phase 3 trial we have the estimates of R for each outcome and a confidence interval (provided by GSK, comparing the two vaccine groups of the trial (R3C and R3R combined) with the control group, over the whole trial period month 0 to study end), and hence can calculate the standard error of log(R). In each country, we calculated a modelled prediction of the rate ratio that would be expected if the safety signal in the phase 3 trial were to occur during the MVIP. The dilution effect is different in each country because coverage of RTSS varied and the degree of contamination (RTSS in comparison areas) also differed. Let the diluted effect in country *i* be R_i' (details of how this was obtained are given below). We can calculate a weighted average of the logged R_i' values, using the same country weights, w_i, that we used in (1): $\sum w_i \log R_i$' . Call this weighted value, F. We need

the standard error of F. Noting that the factor by which the log rate ratio from the phase 3 has been diluted is equal to F/log(R), =k say, and noting that we have the s.e. of log(R) from the phase 3 data, then the s.e. of F is k x s.e. of log(R). To compare the observed pooled log rate ratio (Dpooled) with the (pooled) diluted phase 3 estimate, F, we calculated the difference (Dpooled – F). The standard error of this difference is: se(diff) = sqrt(s.e.(F)^2+ V(Dpooled)), and then we treated (Dpooled-F)/se(diff) as a z-value and obtained a p-value using the normal distribution.

To estimate the proportion of person time spent vaccinated in each country, we considered each month of the evaluation, and for the cohort of children reaching the eligible age (i.e. *s*=5 months in Malawi, *s*=6 months in the other countries) in that month, we summed the Kaplan-Meier estimates of the proportion of children vaccinated by age *a* months from , p(a), multiplied by the person time for that calendar month and age, t(a,m), from the starting age until the end of the evaluation period (up to M months in total, M=25 in Malawi and Ghana (April 2019 to April 2021), M=20 in Kenya Sep 2019 to April 2019), and then divide by the total person time:

$$\sum_{j=1}^{M}\sum_{a=s}^{s+M-j} p(a)t(a,j) \,/\, \sum_{j=1}^{M}\sum_{a=s}^{s+M-j} t(a,j).$$

In the absence of denominator data, the person time t(a,m) was set to 1 for each calendar month and age,xcept the first month which is a partial month depending on the date vaccination started (Apr 23 in Malawi, Apr 30 in Ghana, Sep 13 in Kenya). In Kenya and Ghana, children were eligible if they were aged 6 months or 7 months, and in Kenya if they were 6 months to 11 months old at the start, and the total doses administered, reported by the EPI, was higher in the initial months in these countries, reflecting this catch-up. We estimated the person time in the catch-up cohorts (children who were aged 7 months in the first month in Ghana, and children who were aged 7-11 months in the first month in Kenya) by estimating the average target population per month as the average number of doses administered per month, divided by the survey estimate of coverage, and then allocating the excess doses in the initial months, to the catch-up cohorts to estimate the proportion vaccinated. In Malawi, in practice children were vaccinated at the start only if they were strictly aged 5 months. The survey data included too few RTSS-vaccinated children to be able to estimate proportion vaccinated by month of age in comparison areas of the MVIP, and in non-eligible age groups. We assumed that the proportion of vaccinated person time would be in the same proportion to the proportion eventually vaccinated, as in implementation areas, and applied this to the survey estimates of coverage in comparison areas and in older age groups, to obtain rough estimates of the proportion of vaccinated person time in comparison areas and in older age groups in implementation areas. We assumed no vaccination in older age groups in comparison areas (a conservative assumption in that this assumption results in a greater dilution effect). Finally, we ignored possible effects of confounding that might have further diluted effect estimates in the MVIP. With respect to meningitis, we noted that when PCR analysis identified a causative pathogen, only a small percentage were of serotypes preventable by Hib or pneumococcal vaccine, so although RTSS-recipients were more likely to have received Hib and/or pneumococcal vaccine, the confounding effect was likely to be small. With respect to malaria outcomes, midline surveys suggested RTSS coverage did not differ in relation to malaria prevalence at baseline.

Having got estimates of the proportion of vaccinated person time in implementation and comparator areas, in eligible and non-eligible age groups, in each country, we estimated the expected rate ratio for each safety outcome, if the safety signal from the phase 3 occurred in the MVIP, in each country, as R'=[(Rc+1-c)/(Rd+1-d)]/[(Rf+1-f)/(Rg+1-g)], where c=proportion of vaccinated person time in implementation areas in eligible age groups, d=proportion in comparison areas in eligible age groups, and f and g are the corresponding values in non-eligible groups, for that country. The estimates used were c=0.611 in Malawi,0.690 in Ghana and 0.668 in Kenya; the corresponding proportions in comparison areas were d=0.016, 0.056, 0.087, and in non-eligible age groups in implementation areas, f=0.016 in Malawi and 0.027 in Ghana. In Kenya, older age groups were not surveyed, we assumed the same value for f as in Ghana.

6 Ethical considerations

Meetings with WHO ERC, presentation of programme, including evaluation, took on advice, including importance of social mobilization through Good Participatory Practices. Ethical review of Malaria Vaccine Programme Evaluation was undertaken by participating countries/ partner organizations and the WHO ethics committees. The master and subsequent country specific protocols were submitted to the EMA for review and to WHO and relevant national and institutional review boards (IRB) (listed below) to seek approval of the overall design of the evaluation.

List of ethics committees:

- World Health organisation
  - WHO Ethics Review Committee
- Ghana
  - Ghana Health Service Review Ethics Review Committee
  - Ghana Food and Drugs Authority
- Kenya
  - Scientific and Ethics Review Unit, Kenya Medical Research Institute (KEMRI)
  - US Centers for Disease Control and Prevention (CDC) Institutional Review Board
  - WRAIR Institutional Review Board
  - Oxford Tropical Research Ethics Committee
  - Kenya Pharmacy and Poisons Board
- Malawi
  - College of Medicine Research and Ethics Committee,
  - UNC Chapel Hill Ethics Committee
  - Malawi Pharmacy, Medicines and Poisons Board

National Regulatory Authorities (NRA) jointly reviewed the vaccine under the auspices of AVAREF and with involvement of EMA, followed by individual reviews by each authority following its own processes. All three countries authorized the vaccine for use in pilots.

6.1 Consent

Informed consent for the evaluation data collection

Informed consent was sought for all data collection following IRB approval guidelines. Signed consent was sought from a parent or legal guardian of children who participated in household surveys, for verbal autopsies, and for the use of hospital data for research. Before participation in the feasibility surveys, all potential participants parents/guardians were provided with information about the survey as contained in the informed consent form and written informed consent provided. Individual consent was obtained from parents /guardians prior to the verbal autopsy interviews where a child had died in all three countries and sentinel hospital data collection in Ghana. In Kenya and Malawi, the investigators obtaining permission (consent waiver) to collect medical information from parents /guardians of the children admitted to the sentinel hospitals. However, individual consent was obtained for storage and/or shipment of CSF samples for future molecular testing for pathogens at a reference laboratory.

6.2 Implied consent for vaccine implementation

Implementation of the vaccine was by the Ministry of Health and consent for administration of the vaccine was implied by carers bringing their children to vaccination clinics and accepting the malaria vaccine. In accordance with WHO guidance on implied consent for vaccination, community engagement procedures were designed to provide sufficient information to allow parents and caretakers to make informed decisions on whether to bring age-eligible children to clinics to receive the RTS,S/AS01 vaccine or whether to "opt-out" of RTS,S/AS01 vaccination. Individual written informed consent was not sought for the administration of RTS,S/AS01 as (a) this is not standard practice when a new licensed vaccine is introduced; (b) the scale of the programme would make quality assurance of formal informed consent difficult to assure; and (c) the process of seeking consent would detract from the real-life nature of the programme and likely undermine the evaluation of feasibility and, as a result, impact.

6.3 Role of the Sponsor

WHO developed the master protocol, contracted the country-based evaluation teams and cross-country data management, quality control and analysis team. WHO provided management support and contributed with the core team of investigators to data interpretation and writing. *The evaluation teams had full access to all the data in the study and had final responsibility for the decision to submit for publication.*

7 References

1. Wang X, Theodore MJ, Mair R, Trujillo-Lopez E, du Plessis M, Wolter N, et al. Clinical validation of multiplex real-time PCR assays for detection of bacterial meningitis pathogens. J Clin Microbiol. 2012;50(3):702-8.
2. du Plessis M, de Gouveia L, Freitas C, Abera NA, Lula BS, Raboba JL, et al. The Role of Molecular Testing in Pediatric Meningitis Surveillance in Southern and East African Countries, 2008-2017. J Infect Dis. 2021;224(12 Suppl 2):S194-S203.
3. Ma X, Milligan P, Lam KF, Cheung YB. Ratio estimators of intervention effects on event rates in cluster randomized trials. Stat Med. 2022;41(1):128-45.
4. Milligan P, Moore KM. Statistical report on the results of the RTS,S/AS01 Malaria Vaccine Pilot Evaluation 24 months after the vaccine was introduced World Health Organisation 2021 [Available from: https://terrance.who.int/mediacentre/data/sage/SAGE_eYB_Oct2021.pdf.

8 Supplementary tables

Table S1: Survey dates in each country.

Country	Baseline survey period	Vaccine introduction date	Post introduction survey period
Ghana	15th Jul 2019 – 18th Mar 2019	30th Apr 2019	2nd Nov 2020 – 26th Nov 2020
Kenya	15th Jul 2019 – 16th Dec 2019	13th Sep 2019	3rd Apr 2021 – 13th Aug 2021
Malawi	06th May 2019 – 19th Jun 2019	23rd Apr 2019	11th Sep 2020 – 3rd Apr 2021

Table S2: Case definitions

Outcome	Definition
**All-cause admission**	A stay in hospital/inpatient facility for at least one night, (or patients who were admitted but died before an overnight stay was completed).
**Malaria**	Positive for *P.falciparum* (antigenaemia detected by RDT, or parasitaemia by microscopy at any density if an RDT was not done)
**Admission positive for malaria**	A stay in hospital/inpatient facility for at least one night, (or patients who were admitted but died before an overnight stay was completed), AND positive for malaria
**Hospital Admission**	Admission to a sentinel hospital for any duration (does not require overnight stay)
**Sentinel Hospital Tracer Conditions**	Hospital admissions negative for malaria (by RDT or by slide if RDT was not done), probable or confirmed meningitis (if LP was done), and anaemia (Hb>5 or Hct>15 [if only one of Hb and Hct was measured], or Hb>5 *and* Hct>15 [if both Hb and Hct were measured]), and without a primary clinical diagnosis of malaria (ICD10 codes B50.X), anaemia (ICD10 code D64.9), or meningitis (ICD10 codes G03.X, A17.0, A20.3, A32.1, A39.0, A87.X, B00.3, B01.0, B02.1, B26.1, B37.5, B38.4, G00.X, G01.X, G02.X)
**Impaired Consciousness**	Blantyre Coma Score <3, Glasgow Coma Score <11, or 'P' or 'U' on the AVPU Scale (Alert Verbal Painful Unresponsiveness)
**Multiple or Atypical Convulsions**	>2 episodes within 24 hours or prolonged (>15 minutes), or focal
**Suspected Meningitis**	Hospital admission (any cause), with or without fever and ≥1 of the following:<br>• Neck Stiffness<br>• ≥2 seizures in last 24 hours<br>• Bulging fontanelle<br>• Convulsions (partial, complex febrile or other atypical presentations)<br>• Impaired consciousness<br>• Or, any clinical symptom indicative of meningitis or cerebral malaria that resulted in the conduct of a lumbar puncture by clinical judgement
**Probable meningitis**	Hospital admission with suspected meningitis, AND ≥1 of the following:<br>• CSF is turbid, cloudy or purulent on macroscopic examination<br>• CSF leukocyte count >10 cells/mm^3
**Confirmed meningitis**	Hospital admission with suspected or probable meningitis, AND<br>• CSF is laboratory confirmed by culture or PCR to be of bacterial, viral or other aetiology
**Severe Malaria**	Hospital Admission with Malaria, AND ≥1 of the following:<br>• Impaired consciousness AND NOT positive for probable or confirmed meningitis (those without LP can be included)<br>• Multiple of atypical convulsions AND NOT positive for probable or confirmed meningitis (those without LP can be included)<br>• Respiratory distress (manifested as chest indrawing or deep breathing)<br>• Severe malarial anaemia (Hb <5 g/dL or Hct <15%)
**Severe Malaria (subset)**	Hospital Admission with Malaria, AND ≥1 of the following:<br>• Impaired consciousness AND NOT positive for probable or confirmed meningitis (requires LP)<br>• Multiple or atypical convulsions AND NOT cases positive for probable or confirmed meningitis (requires LP)<br>• Respiratory distress (manifested as chest indrawing or deep breathing)

	• Severe malarial anaemia (Hb <5 g/dL or Hct <15%)
**Cerebral Malaria**	Hospital Admission with malaria, AND<br>• Impaired consciousness AND NOT cases with known probable or confirmed meningitis (those without LP can be included)
**Cerebral Malaria (subset)**	Hospital Admission with malaria, AND ≥1 of the following:<br>• Impaired consciousness AND NOT cases with known probable or confirmed meningitis (requires LP)

Abbreviations: RDT, rapid diagnostic test for malaria; LP, lumbar puncture; Hb, haemoglobin; Hct, haematocrit; ICD, international classifications of diseases; CSF, cerebrospinal fluid; PCR, polymerase chain reaction

Table S3: Data management processes

Task	Description
Data collection development	Electronic data collection tools were developed by each country using Scan form technology (Kenya), ODK (Kenya and Malawi) or Redcap ® (Ghana). The systems were tested, modified and approved prior to its use. Modifications to the tools were also tested and approved during the conduct of the evaluation. The system included skip and check patterns.
Data review by data collectors	Data collectors and their supervisors reviewed their data for accuracy prior to transmission to the country data management teams.
Data review by data managers	Data managers reviewed the data for range and consistency check. Data queries were referred back to the data collectors and their supervisors for resolution.
Reception of data from each country	De-identified Data was delivered through secured mechanisms to LSHTM on monthly basis. Kenya used a secure DropBox, Malawi used Filr (MyFiles) based at LSHTM, and Ghana delivered data using encrypted zip files via email). Once received, the data were processed into the csv/dta/xlsx files and jointly reviewed by data managers based in each country and at LSHTM each month
Reporting to Data Safety Monitory Board (DSMB)	Regular reports of key indicators are delivered to DSMB. These reports are generated using VBA scripts in MS Access database (following the importing of monthly data into the database from the repositiory)
Implementing updates	Each country delivered complete datasets including any changes that have been implemented during the cleaning process.
Audit data	Each country implemented its data auditing process dependent on the platforms they implement.
Agreements	Data sharing agreements are in place and data sharing methodology has been approved by all ethics committee involved in this evaluation.

Table S4 Reasons why lumbar punctures were not done, amongst children with suspected meningitis

	Ghana	Kenya	Malawi
	N=359	N=316	N=891
Insufficient or No information [1]	216 (60.2%)	58 (18.4%)	411 (46.1%)
Contraindication (protocol-specified and other) [2]	65 (18.1%)	23 (7.3%)	89 (10.0%)
LP no longer indicated by clinical judgement [including alternative explanation for symptoms] [3]	44 (12.3%)	118 (37.3%)	195 (21.9%)
Died before LP	2 (0.6%)	77 (24.4%)	19 (2.1%)
Absconded or discharged against medical advice, or parental refusal	22 (6.1%)	33 (10.4%)	104 (11.7%)
Health system challenges (no resources [equipment or staff])	10 (2.8%)	7 (2.2%)	73 (8.2%)

[1] Mostly from early in the program [Ghana: 80% in 2019; Malawi: 77% between November 2019 and March 2020*] - an automatic flag for LP indication was added to the tool which improved the LP rate and, if the LP was not done, reduced missing information on the reasons why. *Prior to November 2019, the number of children with no information or insufficient information on why the LP was not done was low because information on convulsions was not being reliably captured due to a problem with the data collection tool, such that the number of children with suspected meningitis was artificially low; this was rectified in October 2019.

[2] Protocol-specified contraindications were: lateralising signs, cranial nerve lesions, skin infection at the LP site, poor pupillary responses to light, papilloedema, needing resuscitation, and raised intracranial pressure. Other cited contraindications were: low consciousness/unconscious (Kenya and Malawi), 'unfit' for LP (Malawi), respiratory distress (Ghana and Kenya), low platelets (Ghana), unable to keep child still (Ghana), clinically unstable (Ghana), and unspecified contraindications (Ghana, Kenya, and Malawi).

[3] Alternative explanations for symptoms in Ghana were: head injury, malnutrition, febrile convulsions, known seizure disorder, accident, myelomeningocele, epilepsy, poisoning, subarachnoid and intraventricular bleed, and alcohol intoxication. Alternative explanations for symptoms in Kenya were: convulsive disorders, epilepsy, cerebral palsy, febrile convulsions, poisoning, intoxication, shock, drowning, CVA, hydrocephalus, and downs syndrome. Alternative explanations for symptoms in Malawi were: epilepsy, cerebral palsy, and febrile convulsions.

Table S5 Sensitivity analysis for meningitis

	Positivity rate amongst suspected cases who had a LP				Suspected cases without a LP				RR	Bias-adjusted RR	Pooled bias-adjusted RR
	Comparison		Implementing		Comparison		Implementing				
	Eligible	Non-eligible	Eligible	Non-eligible	Eligible	Non-eligible	Eligible	Non-eligible			
**Ghana**	8/104	7/170	9/61	12/177	41	115	43	160	0.67 (0.11, 3.93)	0.51 (0.05, 5.25)	1.03 (0.57, 1.87)
**Kenya**	13/213	10/339	14/208	19/380	57	109	50	100	0.58 (0.25, 1.36)	0.51 (0.20, 1.34)	
**Malawi**	4/205	18/354	5/91	13/268	146	330	125	290	2.23 (0.99, 5.00)	2.00 (0.81, 4.92)	

Within each arm and age-eligibility group, the positivity rate (proportion of tests positive) for probable or confirmed meningitis was determined in those children who had a lumbar puncture. This proportion was then applied to the group of children who had suspected meningitis but did not receive a lumbar puncture, to impute counts for probable or confirmed meningitis cases in this group, assuming that the positivity rate would be the same as for children who had received a lumbar puncture. The rate ratios where then re-estimated using these imputed counts. Pooled RR estimate was 1.14 (95% CI 0.68 – 1.93) and pooled bias adjusted RR was 1.03 (95%CI 0.57 – 1.87).

Table S6 Pathogen of confirmed meningitis cases

	Ghana	Kenya	Malawi
Pathogen	n/N (%)	n/N (%)	n/N (%)
H. influenzae	3/16 (19%)	4/24 (17%)	6/14 (43%)
S. pneumoniae	6/16 (38%)	6/24 (25%)	6/14 (43%)
N. Meningitis	1/16 (6%)	0/24 (0%)	0/14 (0%)
Presumed Enterovirus	6/16 (38%)	14/24 (58%)	2/14 (14%)

Estimates of incidence rate ratios in each country, tests of homogeneity, and pooled estimates, for safety and impact outcomes in age groups of children eligible for at least one dose of RTS,S and in age groups eligible for three doses.

Table S7 IRR among children eligible for at least one dose of RTS,S/AS01: safety outcomes

Outcome	Country	No. of events in implementation areas eligible/non-eligible[a]	No. of events in comparison areas eligible/non-eligible[b]	Rate ratio (RR)[c]	Bias-adjusted RR[d]	s.e. of log of bias-adjusted RR[e]	Test of homogeneity[f] χ2[2df], P-value	Pooled bias-adjusted RR[g] (95%CI)
**Tracer conditions***	Ghana	901/973	535/631	1.09	1.09	0.119	3.44, 0.18	1.04 (0.93,1.17)
	Kenya	544/680	579/640	0.88	0.87	0.110		
	Malawi	751/544	923/763	1.14	1.12	0.081		
**Meningitis**	Ghana	9/12	8/7	0.66	0.40	3.773	0.82, 0.67	0.63 (0.22,1.79)
	Kenya	14/19	13/10	0.57	0.47	0.626		
	Malawi	5/13	4/18	1.73	1.36	1.008		
**Cerebral malaria**	Ghana	12/94	22/82	0.48	0.41	0.815	1.42, 0.49	1.03 (0.61,1.74)
	Kenya	23/71	14/54	1.25	1.18	0.406		
	Malawi	17/62	20/85	1.17	1.12	0.384		
**Severe malaria excluding Cerebral malaria**[χ]	Ghana	80/211	116/209	0.68	0.52	0.819	0.63, 0.73	0.70 (0.53,0.92)
	Kenya	173/356	137/255	0.90	0.84	0.292		
	Malawi	263/506	546/694	0.66	0.66	0.165		
**Cerebral malaria (subset)#**	Ghana	6/35	13/33	0.44	0.30	0.925	1.84, 0.40	0.82 (0.39,1.72)
	Kenya	13/52	11/40	0.91	0.84	0.474		
	Malawi	8/27	8/51	1.89	1.57	0.798		
**Severe malaria excluding cerebral malaria (subset)#** [β]	Ghana	74/177	111/188	0.71	0.61	0.451	0.35, 0.84	0.66 (0.48,0.90)
	Kenya	165/339	131/226	0.84	0.78	0.316		
	Malawi	193/349	424/481	0.63	0.63	0.195		
**Deaths all causes excl. injury, girls**	Ghana	174/429	127/291	0.93	0.92	0.144	1.03, 0.60	0.95 (0.85,1.07)
	Kenya	379/633	362/539	0.89	0.89	0.103		
	Malawi	613/1053	608/1052	1.01	1.01	0.079		
	Ghana	176/458	149/349	0.90	0.89	0.169	0.20, 0.91	0.91 (0.80,1.04)
	Kenya	410/713	376/620	0.95	0.94	0.111		

**Deaths all causes excl. injury, boys**[δ]	Malawi	634/1133	740/1180	0.89	0.89	0.098

The number of events in vaccine-eligible and non-eligible age groups in implementation areas (a) and comparison areas (b), country-specific estimates of the rate ratio (c), bias-adjusted estimates of the country-specific rate ratios (d), standard error of the log of the bias-adjusted rate ratio (e), results of the test of homogeneity across countries for each outcome (f) and the pooled estimate of the rate ratio (g). *tracer conditions are conditions unlikely to be affected by the malaria vaccine, i.e. admissions of any cause excluding patients with a positive malaria test, with anaemia (Hb<5g/dL or HCT<15%), or meningitis; #susbet of cases in which impaired consciousness or convulsions were included as criteria for severe malaria only if a lumbar puncture and CSF examination had been performed (and meningitis, if found, excluded). χ: Test of interaction by cerebral malaria and severe malaria cases excluding cerebral malaria: 1.34 (95%CI 0.80, 2.22), p = 0.26. β: Test of interaction by cerebral malaria and severe malaria excluding cerebral malaria using a subset of cases define above (#): 1.01 (95%CI 0.50, 2.07); p = 0.97. δ: Test of interaction by sex: 1.03 (95%CI 0.88, 1.21); p = 0.71.

Table S8 IRR among children eligible to have received three doses of RTS,S/AS01: safety outcomes

Outcome	Country	No. of events in implementation areas eligible/non-eligible[a]	No. of events in comparison areas eligible/non-eligible[b]	Rate ratio (RR)[c]	Bias-adjusted RR[d]	s.e. of log of bias-adjusted RR[e]	Test of homogeneity[f] χ2[2df], P-value	Pooled bias-adjusted RR[g] (95%CI)
**Tracer conditions***	Ghana	635/973	402/631	1.02	1.01	0.113	6.11, 0.047	1.04 (0.92,1.17)
	Kenya	326/680	365/640	0.84	0.83	0.119		
	Malawi	611/544	703/763	1.22	1.20	0.089		
**Meningitis**	Ghana	6/12	7/7	0.50	0.39	1.402	0.29, 0.86	0.40 (0.12,1.37)
	Kenya	11/19	13/10	0.45	0.37	0.703		
	Malawi	4/13	1/18	5.54	2.27	3.263		
**Cerebral malaria**	Ghana	9/94	15/82	0.52	0.41	0.843	1.34, 0.51	1.05 (0.66,1.67)
	Kenya	17/71	11/54	1.18	1.09	0.300		
	Malawi	17/62	18/85	1.29	1.21	0.411		
**Severe malaria excluding cerebral malaria**[χ]	Ghana	51/211	90/209	0.56	0.44	0.785	0.49, 0.78	0.66 (0.47,0.92)
	Kenya	115/356	95/255	0.87	0.78	0.380		
	Malawi	218/506	468/694	0.64	0.64	0.191		
**Cerebral malaria (subset)#**	Ghana	6/35	13/33	0.44	0.30	0.924	1.75, 0.42	0.72 (0.36,1.44)
	Kenya	9/52	9/40	0.77	0.72	0.409		
	Malawi	8/27	7/51	2.16	1.66	0.904		
**Severe malaria excluding cerebral malaria (subset)#** [β]	Ghana	48/177	82/188	0.62	0.55	0.439	0.19,0.91	0.61 (0.42,0.88)
	Kenya	109/339	92/226	0.79	0.70	0.395		
	Malawi	153/349	354/481	0.60	0.60	0.233		
**Deaths all causes excl. injury, girls**	Ghana	94/429	77/291	0.83	0.82	0.187	0.85,0.65	0.93 (0.82,1.05)
	Kenya	232/633	221/539	0.89	0.89	0.129		
	Malawi	440/1053	456/1052	0.96	0.96	0.075		
	Ghana	97/458	81/349	0.91	0.90	0.198	0.02,0.99	0.89 (0.78,1.02)

**Deaths all causes excl. injury, boys**$^{\delta}$	Kenya	249/713	246/620	0.88	0.88	0.117
	Malawi	477/1133	550/1180	0.90	0.90	0.098

The number of events in vaccine-eligible and non-eligible age groups in implementation areas (a) and comparison areas (b), country-specific estimates of the rate ratio (c), bias-adjusted estimates of the country-specific rate ratios (d), standard error of the log of the bias-adjusted rate ratio (e), results of the test of homogeneity across countries for each outcome (f) and the pooled estimate of the rate ratio (g). *tracer conditions are conditions unlikely to be affected by the malaria vaccine, i.e. admissions of any cause excluding patients with a positive malaria test, with anaemia (Hb<5g/dL or HCT<15%), or meningitis; #susbet of cases in which impaired consciousness or convulsions were included as criteria for severe malaria only if a lumbar puncture and CSF examination had been performed (and meningitis, if found, excluded).). χ: Test of interaction by cerebral malaria and severe malaria cases excluding cerebral malaria: 1.50 (0.92, 2.44); p = 0.10. β: Test of interaction by cerebral malaria and severe malaria excluding cerebral malaria using a subset of cases define above (#): 1.04 (0.61, 1.79); p = 0.88. δ: Test of interaction by sex: 1.02 (0.86, 1.21); p = 0.81.

Table S9 IRR among children eligible for at least one dose of RTS,S/AS01: impact outcomes

Outcome	Country	No. of events in implementation areas eligible/non-eligible[a]	No. of events in comparison areas eligible/non-eligible[b]	Rate ratio (RR)[c]	Bias-adjusted RR[d]	s.e. of log of bias-adjusted RR[e]	Test of homogeneity[f] χ2[2df], P-value	Pooled bias-adjusted RR[g] (95%CI)
**Tracer conditions***	Ghana	901/973	535/631	1.09	1.09	0.119	3.44, 0.18	1.04 (0.93,1.17)
	Kenya	544/680	579/640	0.88	0.87	0.110		
	Malawi	751/544	923/763	1.14	1.12	0.081		
**Severe malaria**	Ghana	92/305	138/291	0.64	0.51	0.665	1.04, 0.59	0.73 (0.55,0.96)
	Kenya	196/427	151/309	0.94	0.88	0.250		
	Malawi	280/568	566/779	0.68	0.68	0.171		
**Severe malaria (subset)#**	Ghana	80/212	124/221	0.67	0.57	0.473	0.43, 0.81	0.69 (0.50,0.94)
	Kenya	178/391	142/266	0.85	0.79	0.293		
	Malawi	201/376	432/532	0.66	0.66	0.205		
**Malaria admissions**	Ghana	463/1538	530/1124	0.64	0.54	0.685	2.96, 0.23	0.83 (0.71,0.97)
	Kenya	386/827	338/710	0.98	0.97	0.120		
	Malawi	689/1480	1213/1962	0.75	0.75	0.103		
**All cause admissions**	Ghana	1577/2811	1202/1996	0.93	0.90	0.237	0.23, 0.89	0.92 (0.80,1.04)
	Kenya	1334/2271	1372/2091	0.90	0.88	0.111		
	Malawi	1834/2532	2472/3245	0.95	0.94	0.085		
**Deaths all causes excl. injury**	Ghana	350/887	276/640	0.91	0.91	0.112	0.10, 0.95	0.93 (0.84,1.02)
	Kenya	789/1346	738/1159	0.92	0.92	0.086		
	Malawi	1247/2186	1348/2232	0.94	0.94	0.069		

The number of events in vaccine-eligible and non-eligible age groups in implementation areas (a) and comparison areas (b), country-specific estimates of the rate ratio (c), bias-adjusted estimates of the country-specific rate ratios (d), standard error of the log of the bias-adjusted rate ratio (e), results of the test of homogeneity across countries for each outcome (f) and the pooled estimate of the rate ratio (g). *tracer conditions are conditions unlikely to be affected by the malaria vaccine, i.e. admissions of any cause excluding patients with a positive malaria test, with anaemia (Hb<5g/dL or HCT<15%), or meningitis; #susbet of cases in which impaired consciousness or convulsions were included as criteria for severe malaria only if a lumbar puncture and CSF examination had been performed (and meningitis, if found, excluded).

Table S10 IRR among children eligible to have received three doses of RTS,S/AS01: impact outcomes

Outcome	Country	No. of events in implementation areas eligible/non-eligible[a]	No. of events in comparison areas eligible/non-eligible[b]	Rate ratio (RR)[c]	Bias-adjusted RR[d]	s.e. of log of bias-adjusted RR[e]	Test of homogeneity[f] χ2[2df], P-value	Pooled bias-adjusted RR[g] (95%CI)
**Tracer conditions***	Ghana	635/973	402/631	1.02	1.01	0.113	6.11, 0.047	1.04 (0.92,1.17)
	Kenya	326/680	365/640	0.84	0.83	0.119		
	Malawi	611/544	703/763	1.22	1.20	0.089		
**Severe malaria**	Ghana	60/305	105/291	0.55	0.44	0.646	0.75, 0.68	0.68 (0.49,0.95)
	Kenya	132/427	106/309	0.90	0.82	0.340		
	Malawi	235/568	486/779	0.66	0.67	0.199		
**Severe malaria (subset)#**	Ghana	54/212	95/221	0.59	0.52	0.456	0.30, 0.86	0.63 (0.44,0.91)
	Kenya	118/391	101/266	0.79	0.71	0.377		
	Malawi	161/376	361/532	0.63	0.63	0.241		
**Malaria admissions**	Ghana	332/1537	373/1124	0.65	0.55	0.681	1.18, 0.55	0.78 (0.66,0.94)
	Kenya	259/827	247/710	0.90	0.89	0.167		
	Malawi	595/1480	1078/1962	0.73	0.73	0.117		
**All cause admissions**	Ghana	1092/2805	861/1996	0.90	0.87	0.220	0.64, 0.73	0.91 (0.80,1.03)
	Kenya	851/2271	905/2091	0.87	0.85	0.116		
	Malawi	1493/2532	2001/3245	0.96	0.95	0.084		
**Deaths all causes excl. injury**	Ghana	191/887	158/640	0.87	0.86	0.145	0.29, 0.86	0.91 (0.82,1.00)
	Kenya	481/1346	467/1159	0.89	0.89	0.095		
	Malawi	917/2186	1006/2232	0.93	0.93	0.069		

The number of events in vaccine-eligible and non-eligible age groups in implementation areas (a) and comparison areas (b), country-specific estimates of the rate ratio (c), bias-adjusted estimates of the country-specific rate ratios (d), standard error of the log of the bias-adjusted rate ratio (e), results of the test of homogeneity across countries for each outcome (f) and the pooled estimate of the rate ratio (g). *tracer conditions are conditions unlikely to be affected by the malaria vaccine, i.e. admissions of any cause excluding patients with a positive malaria test, with anaemia (Hb<5g/dL or HCT<15%), or meningitis; #susbet of cases in which impaired consciousness or convulsions were included as criteria for severe malaria only if a lumbar puncture and CSF examination had been performed (and meningitis, if found, excluded).

Table S11 Sensitivity analysis including cases from outside the pre-specified sentinel areas

	Events in age-eligible children		Rate Ratio (95%CI)				
**Outcome**	**Vaccinating areas**	**Comparison areas**	**Pooled**	**Ghana**	**Kenya**	**Malawi**	**Pooled - bias adjusted**
Probable or confirmed meningitis (children eligible for at least one dose of RTS,S/AS01)	29	28	0.93 (0.42, 2.07)	0.84 (0.01, 83.60)	0.75 (0.29, 1.96)	1.73 (0.33, 9.20)	0.77 (0.34, 1.70)
Cerebral malaria (children eligible for at least one dose of RTS,S/AS01)	52	64	0.96 (0.59, 1.54)	0.46 (0.14, 1.55)	1.05 (0.49, 2.24)	1.15 (0.53, 2.51)	0.90 (0.56, 1.46)
Cerebral malaria (subset) (children eligible for at least one dose of RTS,S/AS01)	27	37	0.77 (0.43, 1.36)	0.39 (0.14, 1.09)	0.84 (0.36, 1.96)	1.89 (0.47, 7.63)	0.67 (0.38, 1.19)
Severe malaria (children eligible to have received three doses of RTS,S/AS01)	434	745	0.73 (0.55, 0.97)	0.57 (0.22, 1.47)	0.87 (0.54, 1.38)	0.66 (0.43, 1.01)	0.70 (0.53, 0.94)
Severe malaria (subset) (children eligible to have received three doses of RTS,S/AS01)	338	604	0.68 (0.50, 0.92)	0.59 (0.30, 1.18)	0.78 (0.48, 1.29)	0.63 (0.38, 1.05)	0.65 (0.48, 0.88)

Clusters outside the sentinel area were only included if there was at least one case in either the age-eligible or age-ineligble groups.
In Malawi data were not collected on admissions from outside the sentinel area, so estimates are unchanged.

9 Supplementary figures

Figure S1A - C. Maps of evaluation areas

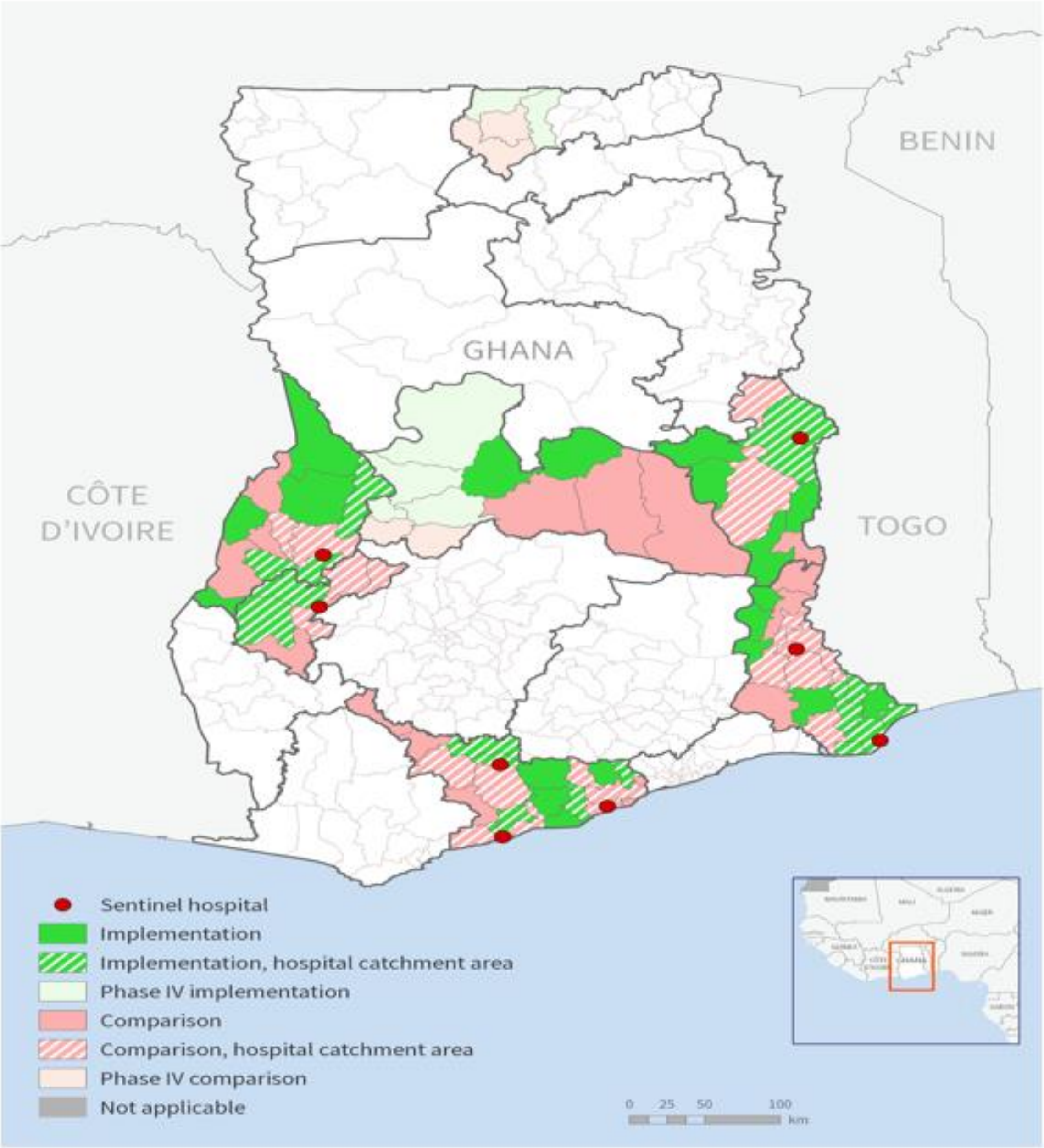

A

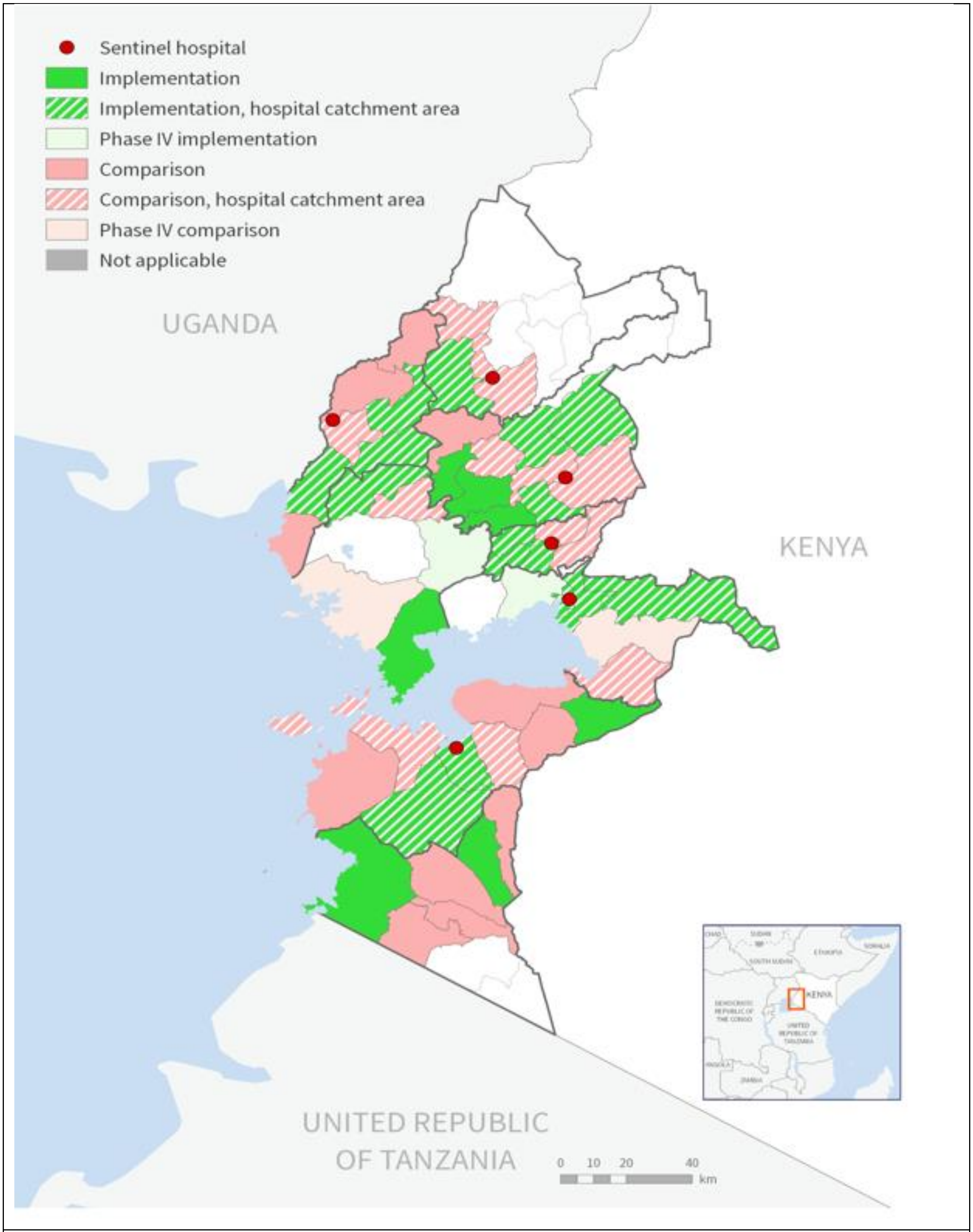
Sentinel hospital
Implementation
Implementation, hospital catchment area
Phase IV implementation
Comparison
Comparison, hospital catchment area
Phase IV comparison
Not applicable
UGANDA
KENYA
UNITED REPUBLIC
OF TANZANIA
0 10 20 40
km

B

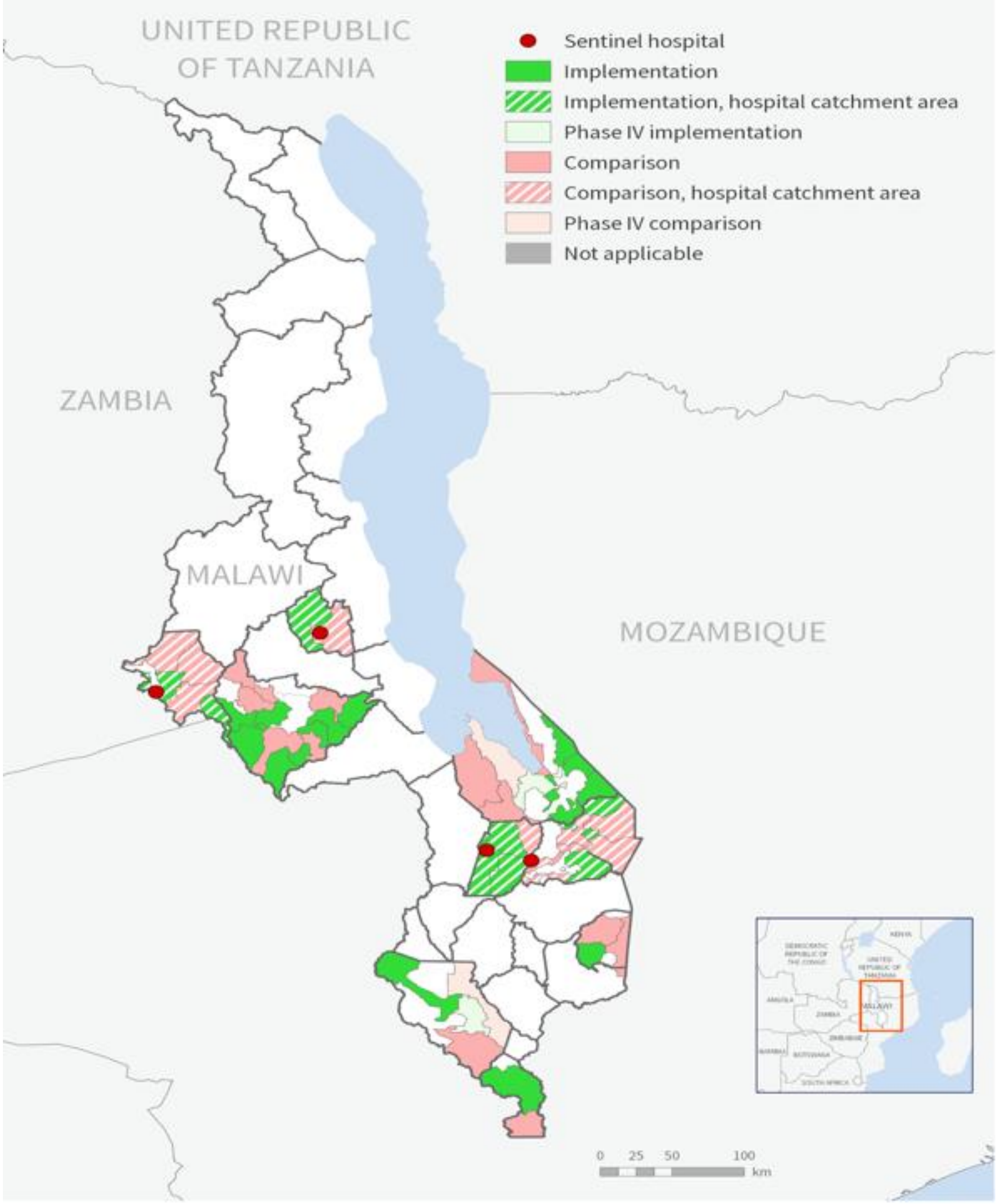
UNITED REPUBLIC OF TANZANIA
Sentinel hospital
Implementation
Implementation, hospital catchment area
Phase IV implementation
Comparison
Comparison, hospital catchment area
Phase IV comparison
Not applicable
ZAMBIA
MALAWI
MOZAMBIQUE
0 25 50 100
km

C

Maps showing areas that were allocated to deliver RTS,S vaccination starting in 2019 (pink) and comparison areas (green) where RTSS implementation would be delayed, in (A) Ghana, (B) Kenya, and (C) Malawi. Areas in Ghana are districts, subcounties in Kenya, and catchment areas of groups of immunization clinics in Malawi. Red dots indicate the location of sentinel hospitals in each country.

Figure S2 Vaccination schedule for RTSS and childhood immunization schedule

Vaccine/1 — Child Age	Birth	6 wks	10 wks	14 wks	5 mo	6 mo	7 mo	9 mo	12 mo	18 mo	22 mo	24 mo
BCG	1											
Oral polio	0	1	2	3								
DTP-HepB-Hib (penta)		1	2	3								
Pneumococcal conj.		1	2	3								
Rotavirus		1	2									
Inactivated Polio				1								
Meningococcal A conj.										1		
Measles-Rubella								1		2		
Yellow Fever								1				
RTS,S in Ghana						1	2	3				4
RTS,S in Kenya						1	2	3				4
RTS,S in Malawi					1	2	3				4	
Vitamin A						1			2	3		4
Growth Monitoring	●	●	●	●	●	●	●	●	●	●	●	●
Deworming												●

Figure S3 Parameters of age-eligible and age–ineligible populations by country.

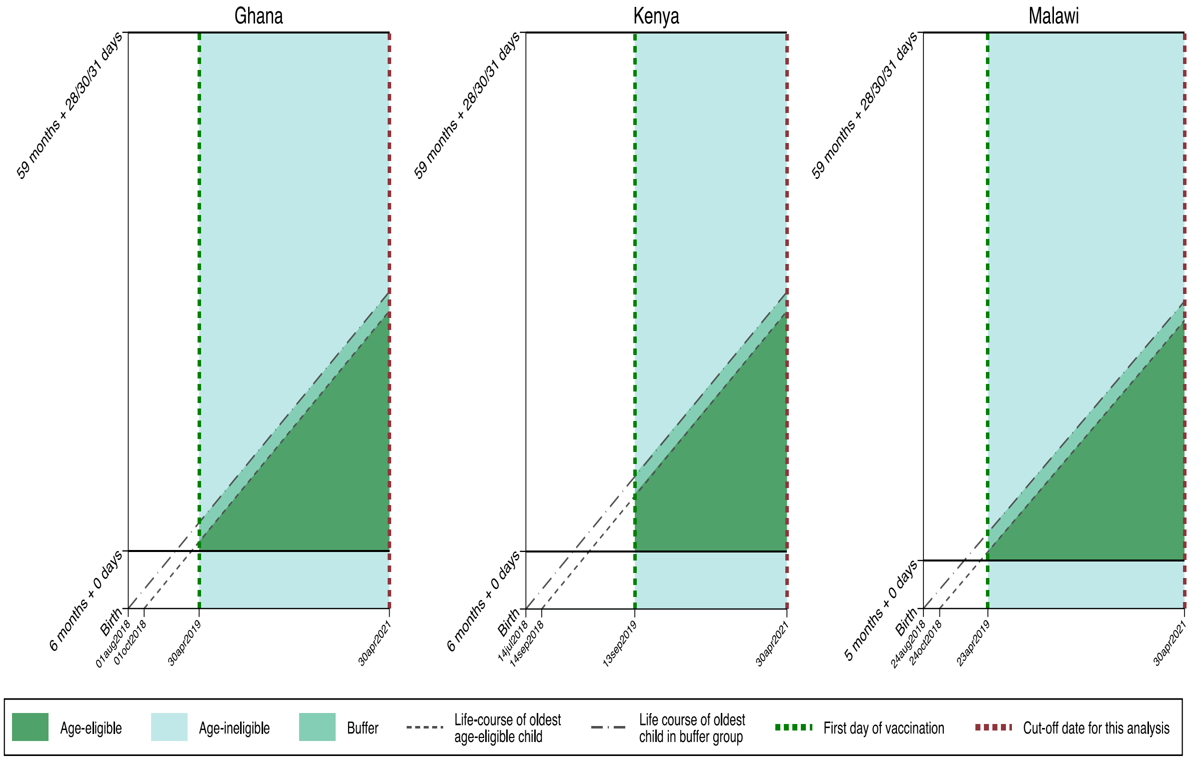

The ‘buffer’ group includes children who are 1 or 2 months too old to be considered age-eligible, and children in this group are excluded from the analysis to reduce the possibility of a vaccinated child falling in the age-ineligible group. For safety endpoints (meningitis, cerebral malaria, and the mortality gender interaction), the effect of RTS,S is determined within the population of children eligible for RTS,S (any dose). For impact endpoints (severe malaria), the effect of RTS,S is determined within the population of children eligible to have received RTS,S dose 3.

Figure S4 Vaccine Coverage and childhood interventions by country, restricted to sentinel hospital area

**Restricted to Sentinel**					
**Ghana**	N=1079	N=1176	N=1163	N = 1148	
Penta dose 3 coverage by HBR or recall[1]	90 (85-96)	95 (93-97)	90 (86-94)	91 (88-95)	1·06 (0·97-1·15); p = 0·206
Measles dose 1 coverage by HBR or recall[1]	88 (84-92)	90 (87-94)	86 (83-90)	87 (83-90)	1·04 (0·95-1·13); p = 0·379
Full basic vaccination coverage[1,2]	75 (69-81)	79 (74-84)	73 (69-76)	76 (71-81)	1·03 (0·91-1·17); p = 0·639
Vitamin A dose received within last 6 months[3]	49 (44-54)	50 (44-55)	54 (48-61)	52 (48-57)	1·04 (0·83-1·31); p = 0·739
Slept under bednet previous night	58 (51-66)	61 (56-67)	58 (49-68)	63 (57-69)	0·98 (0·76-1·25); p = 0·848
Boys	57 (50-66)	61 (55-67)	56 (46-68)	61 (53-69)	0·98 (0·73-1·31); p = 0·893
Girls	58 (50-68)	62 (55-71)	59 (50-71)	65 (58-73)	0·97 (0·73-1·30); p = 0·835
Reported fever in past two weeks	20 (16-26)	22 (18-28)	15 (12-19)	17 (14-21)	0·96 (0·62-1·48); p = 0·841
Sought advice or treatment for fever	66 (56-76)	85 (77-92)	61 (46-75)	81 (70-92)	0·95 (0·70-1·31); p = 0·771
Received blood test for treatment	27 (18-36)	48 (37-59)	26 (13-40)	56 (44-68)	0·84 (0·43-1·65); p = 0·614
**Kenya**	N = 644	N = 694	N = 1438	N = 1636	
Penta dose 3 coverage by HBR or recall	91 (88-94)	93 (88-98)	94 (92-96)	93 (91-96)	1·03 (0·96-1·11); p = 0·402
Measles dose 1 coverage by HBR or recall	84 (79-90)	93 (89-96)	90 (88-92)	90 (88-92)	1·10 (1·01-1·19); p = 0·028
Full basic vaccination coverage[2]	69 (64-76)	78 (72-85)	81 (79-84)	82 (80-85)	1·11 (0·98-1·26); p = 0·103
Vitamin A dose received within last 6 months[3]	43 (37-51)	45 (36-56)	51 (47-56)	52 (47-58)	1·03 (0·96-1·11); p = 0·402
Slept under bednet previous night	88 (84-93)	92 (87-98)	93 (91-96)	92 (90-95)	1·10 (1·01-1·19); p = 0·028
Boys	88 (84-93)	92 (86-98)	94 (91-96)	90 (84-97)	1·11 (0·98-1·26); p = 0·103
Girls	89 (83-94)	93 (87-99)	93 (90-96)	94 (92-96)	1·04 (0·94-1·14); p = 0·461
Reported fever in past two weeks	43 (37-50)	49 (40-60)	32 (29-35)	29 (24-34)	1·24 (0·90-1·70); p = 0·186
Sought advice or treatment for fever	70 (62-80)	68 (63-74)	70 (65-76)	73 (64-82)	0·94 (0·76-1·15); p = 0·532
Received blood test for treatment	58 (51-65)	63 (56-72)	46 (39-54)	51 (41-62)	1·00 (0·73-1·35); p = 0·981
**Malawi**	N = 770	N = 694	N = 1400	N = 1340	
Penta dose 3 coverage by HBR or recall	88 (84-92)	88 (83-94)	88 (84-92)	86 (84-89)	1·03 (0·94-1·12); p = 0·583
Measles dose 1 coverage by HBR or recall	85 (80-89)	81 (75-87)	83 (78-88)	84 (80-89)	0·94 (0·83-1·05); p = 0·268
Full basic vaccination coverage[2]	77 (71-84)	77 (70-85)	78 (72-84)	77 (74-81)	1·00 (0·85-1·17); p = 0·969
Vitamin A dose received within last 6 months[3]	65 (57-74)	53 (43-64)	77 (71-84)	72 (66-80)	0·86 (0·66-1·11); p = 0·248
Slept under bednet previous night	86 (80-91)	94 (92-97)	69 (62-76)	73 (66-79)	1·05 (0·90-1·22); p = 0·510
Boys	86 (79-93)	94 (90-98)	70 (63-78)	68 (61-76)	1·13 (0·95, 1·34); p = 0·177
Girls	85 (72-1·00)	95 (92-99)	68 (60-77)	76 (67-87)	1·00 (0·78-1·28); p = 0·999
Reported fever in past two weeks	33 (29-39)	37 (28-49)	23 (19-28)	35 (28-43)	0·73 (0·47-1·12); p = 0·144
Sought advice or treatment for fever	79 (72-87)	71 (62-80)	73 (64-85)	78 (70-86)	0·84 (0·67-1·06); p = 0·151
Received blood test for treatment	65 (52-83)	48 (39-59)	53 (42-68)	66 (56-77)	0·60 (0·40-0·91); p = 0·016

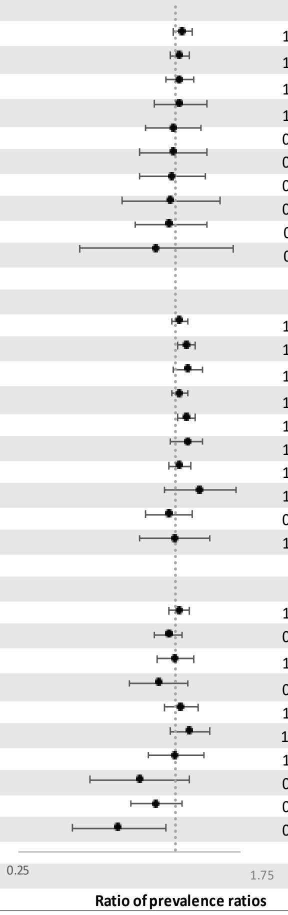

Figure S5 Hospital admissions flowchart

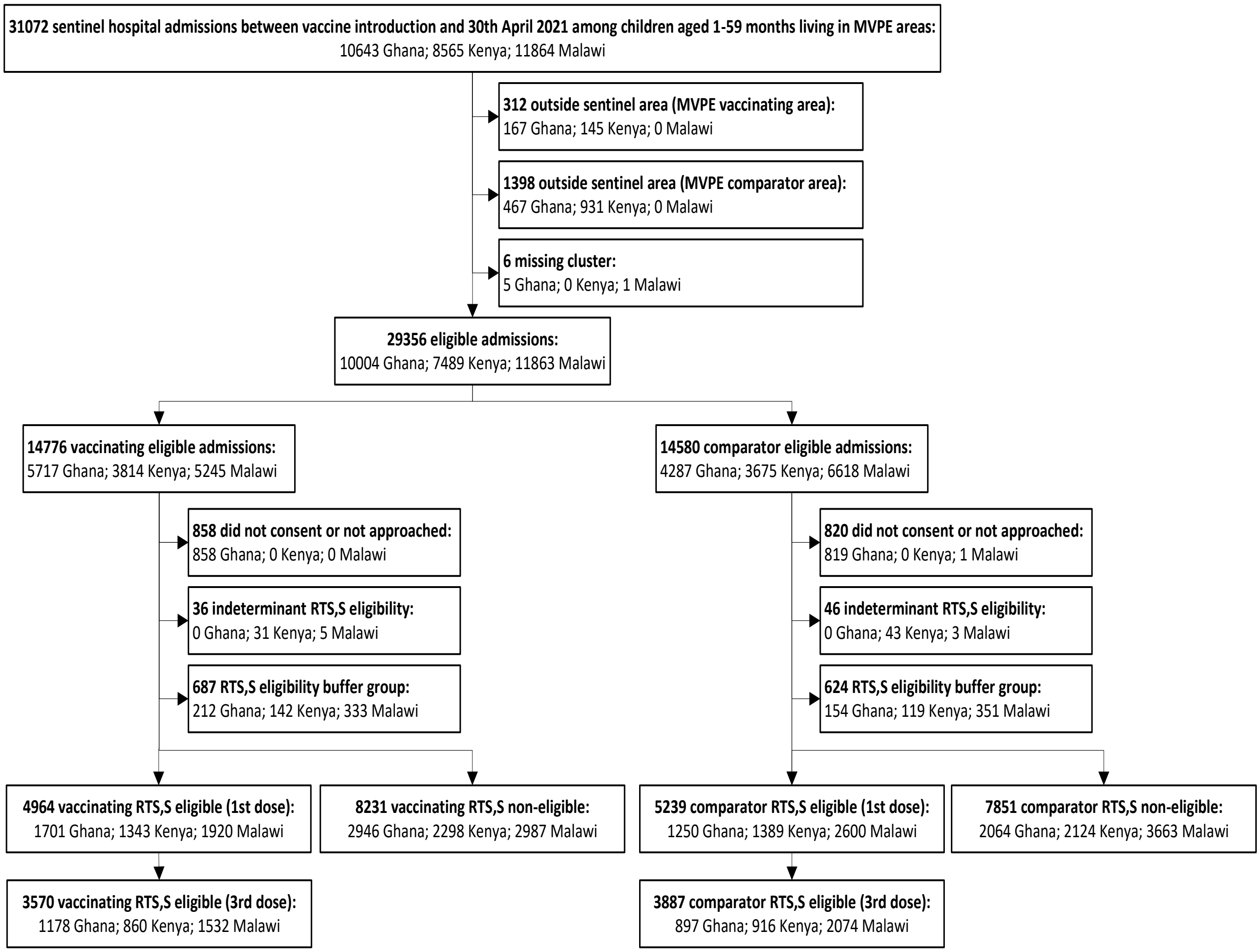

Figure S6 Hospital admissions flowchart.

Hospital admissions flowchart, showing children eligible for at least one dose of RTS,S, and the subset of these who were eligible for three doses, and children in the non-eligible group. **Sentinel areas:** Patients admitted from outside the defined catchment areas were excluded from the primary analyses, as there was no systematic surveillance for hospital outcomes outside the 77 clusters in the pre-defined catchment areas, and the randomization aimed to balance total admissions from RTS,S and comparison areas within these 77 sentinel clusters only. Secondary analysis including patients in Ghana and Kenya that were from outside the defined surveillance area is shown in Table S12. **Consent:** In the early part of the study, consent was not sought in Ghana for patients without clinically suspected malaria or meningitis; of the 858 vaccinating and 819 comparator children excluded in Ghana due to lack of consent, 680 and 486, respectively, were children without clinically suspected malaria or meningitis at admission who were not approached for consent. **Eligibility:** In the analyses, patients are classified as to whether they would have been eligible to receive RTS,S, based on their date of birth, and within these a subset is defined that would have been eligible to have received three doses of RTS,S (Figure S3). Non-eligible patients are those who were too young to have been vaccinated (less than 5 months in Malawi, less than 6 months in Ghana and Kenya), and those who were too old (by at least 2 months) when the vaccine was introduced (Figure S3). Children

who were just too old, by up to 2 months, to have been vaccinated (referred to as the ‘buffer’ group), were excluded from analyses (Figure S3). These criteria are applied in the same way in RTS,S implementation and comparison areas. Eligibility could not be determined for children with missing date of birth and whose recalled age was missing or was rounded down to whole or half years such that the range of plausible ages in months did not fall entirely within a single eligibility category.

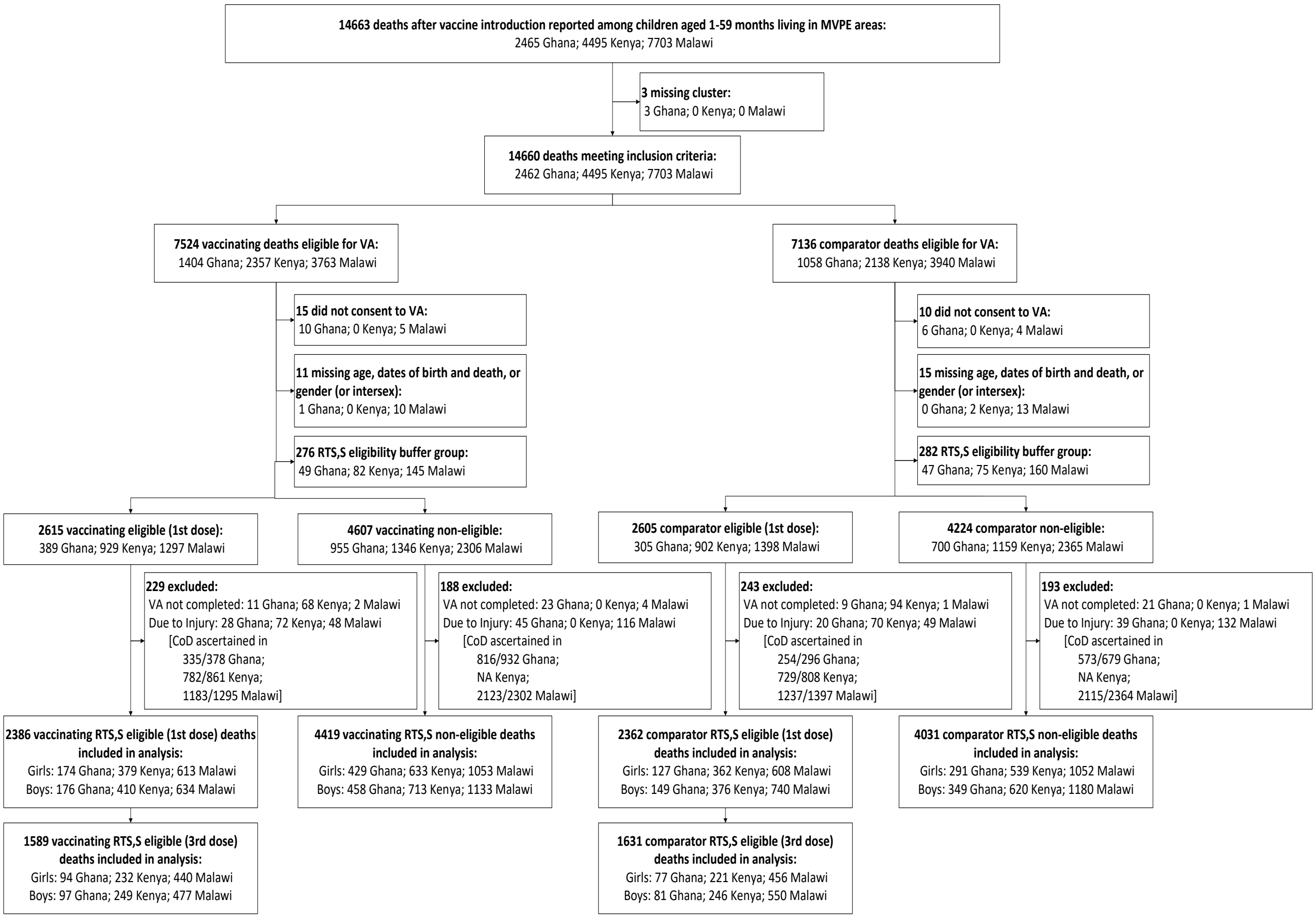

Figure S7 Flowchart showing deaths included in the analyses.

Eligibility: Children who died were classified according to their eligibility to have received RTS,S, as described for hospital admissions (Figure S5) and in Figure S3. **Verbal autopsies (VA):** In Ghana, verbal autopsies (VAs) were performed for all deaths aged 1-59 months. In Malawi, VAs were performed for all community deaths aged 1-59 months; 1308 of 7366 deaths were captured through health facilities rather than in the community, for which the cause of death could be determined from the health facility death notification, and only a "mini" VA was conducted, for the purpose of capturing vaccination status. In Kenya, VAs were performed for all deaths aged 5-48 months, but not for deaths under 5 months of age and for deaths above 48 months of age. Analyses were therefore based on deaths excluding injuries, in eligible and non-eligible groups, in Ghana and Malawi, and in deaths excluding injury (in eligible groups) and all deaths (in non-eligible groups) in Kenya. **Cause of death:** Cause of death (CoD) ascertainment from Inter-VA analysis (or death notification for Malawi facility deaths); children with unknown CoD were assumed not to have died from injury and were retained in analyses.

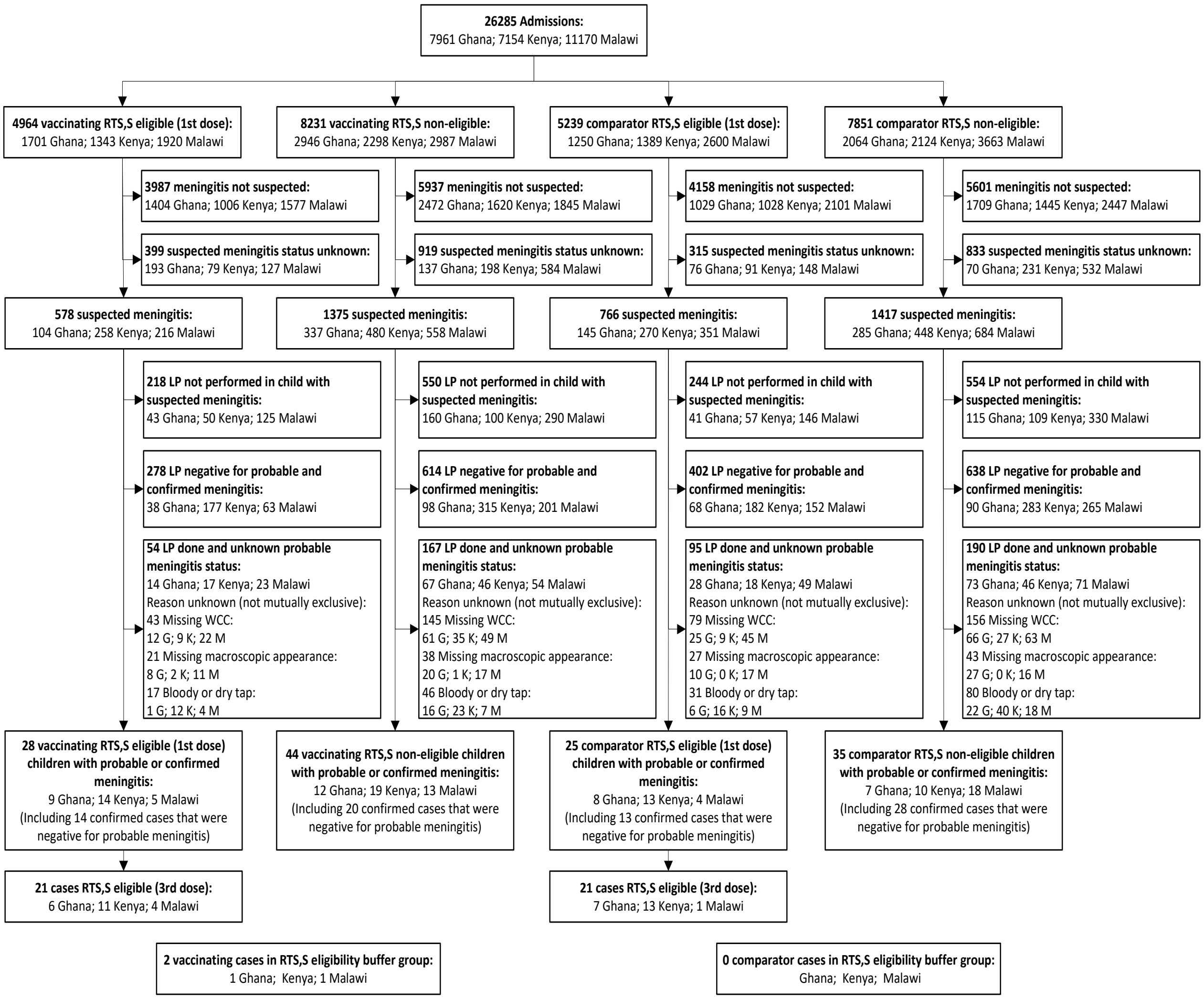

Figure S8 Probable or Confirmed Meningitis Analytic Population from Hospital Admissions

Malaria Positive by RDT or by slide if RDT was not done. **LP:** lumbar puncture. **WCC:** White Cell Count. **Eligibility:** Children were classified according to their eligibility to have received RTS,S, as described for hospital admissions (Figure S5) and in Figure S3. **Missing data:** Unknown suspected meningitis status is due to missing data on one or more indicators for suspected meningitis, where the child does not otherwise meet the criteria for suspected meningitis. Unknown probable meningitis status in children who had a lumbar puncture is due to either missing white cell count (WCC), missing CSF (cerebrospinal fluid) macroscopic appearance, or a bloody or dry tab, in a child who does not otherwise meet the criteria for probable or confirmed meningitis. **Probable vs. confirmed cases:** there were cases in all countries who were negative for probable meningitis (WCC $\leq$10 cells/mm^3 and clear CSF), but positive for confirmed meningitis by PCR. Descriptive statistics on the proportion of CSF samples that had PCR testing done, a breakdown of confirmed meningitis cases by probable meningitis status, and a breakdown of the specific causative pathogens of confirmed meningitis cases are in Supplement T6. **Definitions:** Refer to Supplement T2 for case definitions.

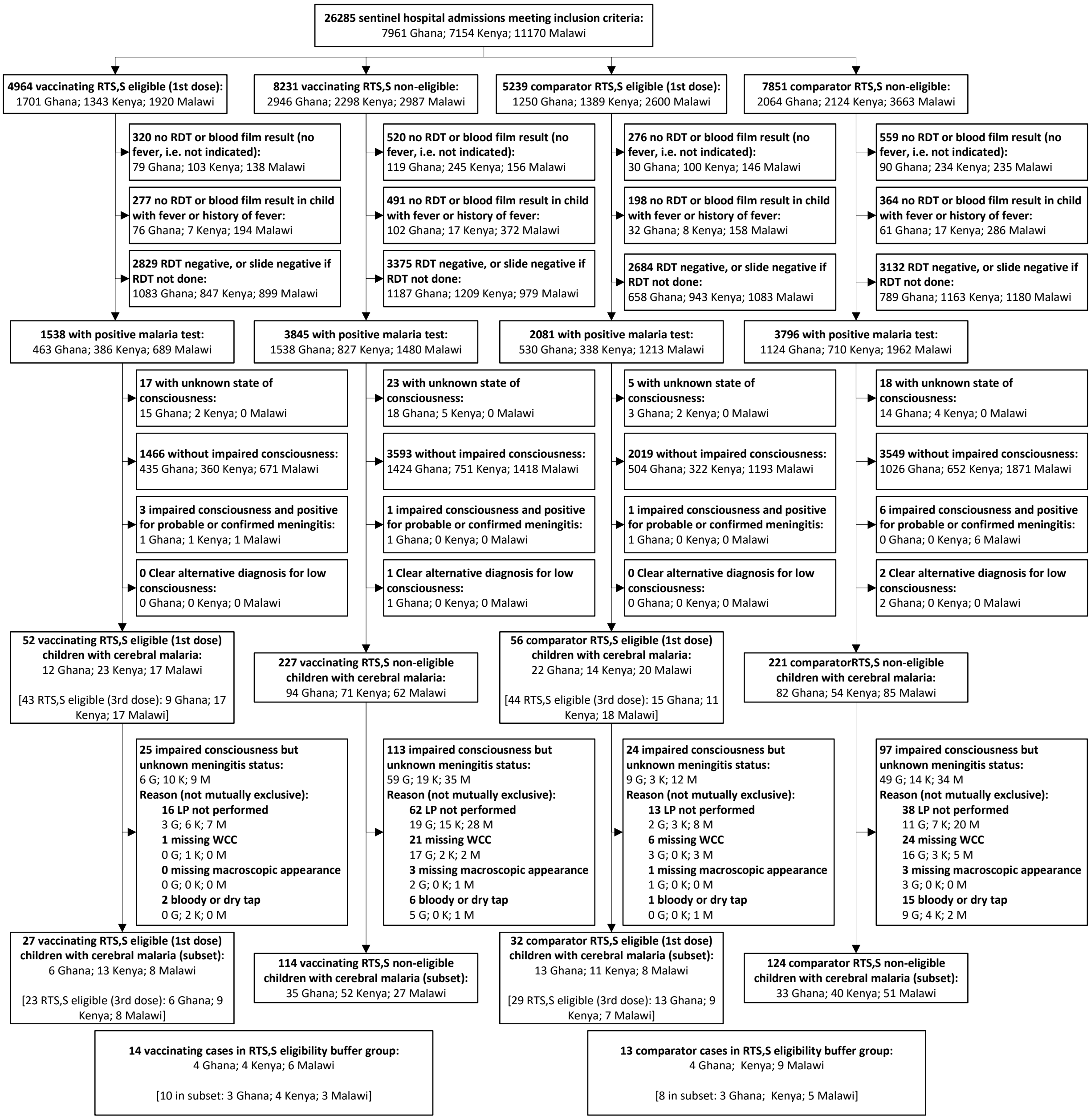

Figure S9 Cerebral Malaria (CM) Analytic Population from Hospital Admissions

Malaria: positive by RDT or by slide if RDT was not done. **LP:** lumbar puncture. **WCC:** White Cell Count. **Eligibility:** Children were classified according to their eligibility to have received RTS,S, as described for hospital admissions (Figure S5) and in Figure S3. **Missing data:** Unknown state of consciousness is due to missing data for Glasgow Coma Score (GCS), Blantyre Coma Score (BCS) and/or AVPU score [Alert, Voice, Pain, Unresponsive]) or where the child does not otherwise meet the criteria for impaired consciousness. Unknown probable meningitis status in children who had a lumbar puncture is due to either missing white cell count (WCC), missing CSF (cerebrospinal fluid) macroscopic appearance, or a bloody or dry tab, in a child who does not otherwise meet the criteria for probable or confirmed meningitis. **Subset:** Analyses were performed including cases with unknown

meningitis status, and also within the subset of cases excluding those with unknown meningitis status. **Definitions:** Refer to Supplement T2 for case definitions.

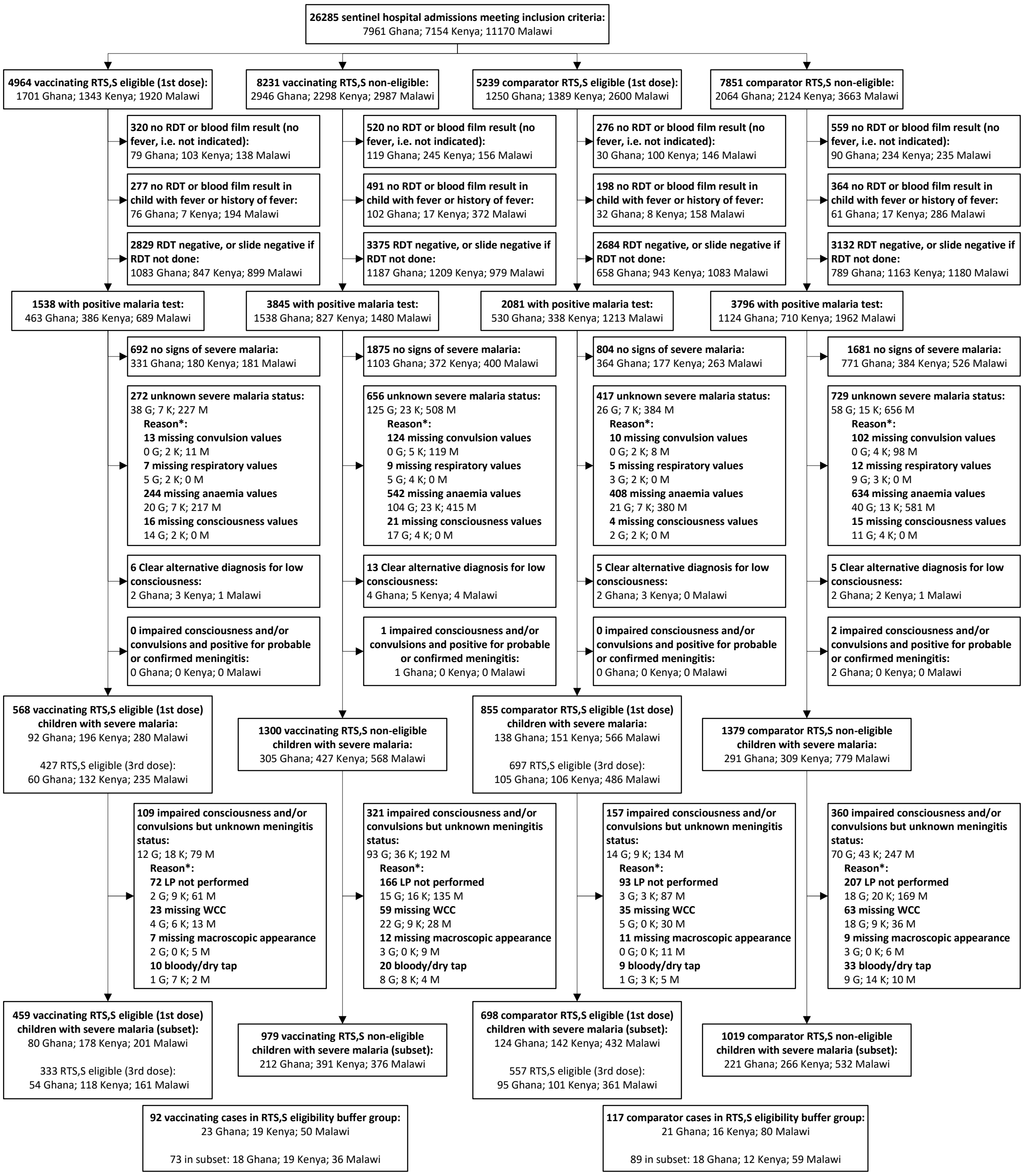

Figure S10 Severe Malaria Analytic Population from Hospital Admissions

*not mutually exclusive. **Malaria:** positive by RDT or by slide if RDT was not done. **LP:** lumbar puncture. **WCC:** White Cell Count. **Eligibility:** Children were classified according to their eligibility to have received RTS,S, as described for hospital admissions (Figure S5) and in Figure S3. **Missing data:** Convulsion values include the presence of convulsions, and in children with convulsions, the number of convulsions in the past 24 hours, and the presence of atypical convulsions. Respiratory values

include chest indrawing and deep breathing. Consciousness values include on or more of Glasgow Coma Score (GCS), Blantyre Coma Score (BCS) and/or AVPU score [Alert, Voice, Pain, Unresponsive]) . Anaemia values includes haemoglobin and/or haematocrit. Children are not excluded due to missing data on these values if they otherwise meet the criteria for severe malaria. Unknown probable meningitis status in children who had a lumbar puncture is due to either missing white cell count (WCC), missing CSF (cerebrospinal fluid) macroscopic appearance, or a bloody or dry tab, in a child who does not otherwise meet the criteria for probable or confirmed meningitis. **Subset:** Analyses were performed including cases with unknown meningitis status, and also within the subset of cases excluding those with unknown meningitis status. **Definitions:** Refer to Supplement T2 for case definitions.
